# ﻿A compendium of macrofungi of Pakistan by ecoregions

**DOI:** 10.3897/mycokeys.89.81148

**Published:** 2022-05-09

**Authors:** Nourin Aman, Abdul Nasir Khalid, Jean-Marc Moncalvo

**Affiliations:** 1 Department of Botany, University of the Punjab, Quaid-e-Azam Campus, Lahore, 54590, Pakistan University of the Punjab Lahore Pakistan; 2 Department of Natural History, Royal Ontario Museum, 100 Queen’s Park, Toronto, Ontario M5S 2C6, Canada Department of Natural History, Royal Ontario Museum Toronto Canada; 3 Department of Ecology and Evolutionary Biology, University of Toronto, 25 Willcocks Street, Ontario M5S 3B2, Canada University of Toronto Toronto Canada

**Keywords:** Biodiversity, conservation, ecoregions, fungi, taxonomic list

## Abstract

Macrofungi form fruiting bodies that can be detected with the naked eye in the field and handled by hand. They mostly consist of basidiomycetes, but also include some ascomycetes. Mycology in Pakistan is still in its infancy, but there have been many historical reports and checklists of macrofungi occurrence from its 15 ecoregions, which range from Himalayan alpine grasslands and subtropical pine forests to deserts and xeric shrublands. In this work, we searched and reviewed the historical literature and the GenBank database for compiling a comprehensive list of macrofungi reported from Pakistan to date. We recorded 1,293 species belonging to 411 genera, 115 families and 24 orders. These occurrences were updated taxonomically following the classification system currently proposed in the Index Fungorum website. The highest represented order by taxon number is Agaricales (47%) with 31 families, 146 genera and 602 species, followed by Polyporales (11%), Russulales (9%) and Pezizales (8%). Genera occurrence reported therein are presented for each ecoregion to the best of our ability given the data. We also discussed the currently known macrofungi diversity between different ecoregions in Pakistan. Overall, this work should serve as a solid foundation for the inclusion of Pakistan macrofungi in global biodiversity and conservation studies.

## ﻿Introduction

Fungi are amongst the most diverse groups of organisms on earth. There have been numerous estimates regarding the total number of fungi worldwide. Bisby and Ainsworth (1943) recorded the total number to be about 100,000 and later, Hawksworth (1991) hypothesised the total number of fungal species to be 1.5 million. Later, Blackwell (2011) estimated the total number of fungi to be around 3.5 – 5.1 million. More recently, Hawksworth and Lucking (2017) predicted the total number to be in the range of 2.8 to 3.8 million. To date, 149,974 species have been recognised (Index Fungorum 2021). The current rate of fungal species discovery per year averages at 2,000 as compared to 1,000 to 2,000 a decade ago (Cheek et al. 2020).

Macrofungi form fruiting bodies that can be detected with the naked eye in the field and handled by hand. They mostly consist of basidiomycetes, but also include some ascomycetes. They play many essential roles in ecosystems as mutualists, pathogens, decomposers or saprotrophs (Volk 2013). Some are edible, medicinal or toxic to humans. About 20,000 macrofungal species have been recognised worldwide (Hawksworth 2001), but many belong to cryptic species complexes and many more await discovery, particularly from poorly explored regions of the world.

A major hindrance of traditional systematics in fungal discovery and identification is the presence of limited taxonomic features (Wu et al. 2019). The traditional identification techniques utilised morphological features, ecological characters, physiology and biochemistry of tissues (Wang et al. 2016). The boom in molecular methods in the 1980s and a remarkable paper by White et al. (1990) describing rRNA primers in fungi spurred the beginning of molecular data utilisation in fungal classification and species identification. Phylogenetic studies have shown that morphologically similar taxa might belong to different lineages (e.g. Hibbett et al. 1997; Moncalvo et al. 2002). DNA sequences can also be helpful for detecting and distinguishing amongst cryptic taxa sharing similar morphological traits (e.g. Moncalvo and Buchanan 2008; Schoch et al. 2012; Wu et al. 2019).

Before the partition of British India, mycoflora of the region (presently India and Pakistan) was listed by Butler and Bisby (1931) and Mundkur (1938). These checklists recorded only 198 species of this region expanding on 30,000 square miles (77700 km^2^). Later, Ahmad et al. (1997) recorded about 4,500 fungal taxa in a list that included all groups of fungi, i.e. macrofungi as well as microfungi and lichens. In the last two decades, many new records and description of new species have been added, based on morphological characters alone or in combination with molecular data (e.g. Sarwar et al. 2011; Saba et al. 2019a; Bashir et al. 2020a; Khalid in press), but none of these was comprehensively addressing macrofungal diversity in Pakistan and the ecoregions of their occurrence.

From a biodiversity conservation perspective, ecologists have been concerned about the factors that affect the delimitation of ecological units and how it affects our knowledge of ecological processes (Weins et al. 1985; Gosz 1991). Numerous efforts have been made to categorise geographical zones with analogous features. In a remarkable paper, Olson et al. (2001) defined ecoregions as broad areas of land or water that consist of geographically distinct assemblages of taxa, natural communities and environmental conditions. They presented an ecoregion map for its utilisation at global as well as regional scales. They based their map on biogeographic information and this was built with the collaboration of more than 1,000 experts in biogeography, taxonomy, conservation biology and ecology from all over the world. Ecoregions were classified by taking into account biogeographic features like endemism, species richness and special evolutionary perspectives. The unique feature of this global biodiversity map is that it focuses on species allocation and communities more precisely than the earlier models, based on biophysical characters, for instance, rainfall and temperature (Holdridge 1967; Walter and Box 1976; Schultz 1995; Bailey 1998) or vegetation structure (UNESCO 1969; de Laubenfels 1975; Schmidthüsen 1976). In Olson et al. (2001), the terrestrial world is divided into 14 biomes, eight biogeographic realms and 867 ecoregions; out of which, nine biomes, two realms and 15 ecoregions are found in Pakistan. This country covers a wide altitudinal range from sea level (Arabian Sea) to the second highest peak of the world, K2. The variety of ecoregions from Himalayan alpine grasslands and subtropical pine forests to deserts and xeric shrublands promotes a great deal of fungal diversity that still remains largely unaccounted for.

In this study we compiled a compendium of macrofungi reported from Pakistan to date from searches in the historical literature as well as in the GenBank database. We have included fungi with prominent fruiting bodies visible to the naked eye in this taxonomic list. We have excluded taxa in Ascomycota which are immersed or half immersed structures, galls or non-prominent fruiting structures on animal dung. We also categorised the reported macromycetes into ecoregions, based on available data.

## ﻿Materials and methods

### ﻿Compendium of Macromycetes of Pakistan

For compiling a comprehensive compendium of macromycetes of Pakistan, data were gathered from extensive literature searches of checklists and published papers, as well as in the GenBank sequence database. Sequence data in GenBank (2020, 2021) was retrieved using a Python script written by Santiago Sanchez-Ramirez (available upon request) on 09-10-2020 for Basidiomycota and on 22-06-2021 for Ascomycota. The list was arranged following the current classification system in Index Fungorum (2021) with great care about eliminating synonymy.

### ﻿Division of macromycetes of Pakistan into Ecoregions

In order to attribute ecoregion occurrence of the taxa we retrieved, we used their locality-based information to consult various repositories, such as Ecoregion 2017 (Dinerstein et al. 2017), DOPA explorer (Dubois et al. 2018) and the ArcGis search tool (2021). Ecoregion allocation of genera was graphically represented on an MS excel spreadsheet for analyses. Genera were listed in rows and ecoregions in columns. The presence or absence of a genus in an ecoregion was scored “1” or “0”, respectively. The sum and percentage of each genus in each ecoregion were then calculated.

## ﻿Results

**Figure 1.**
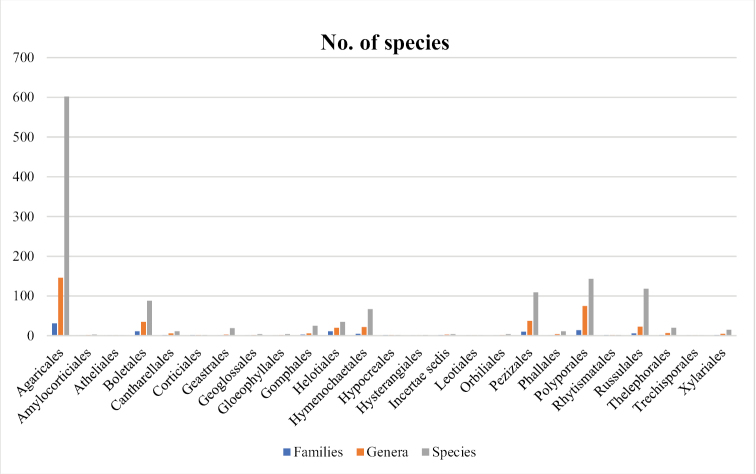
The Bar graph showing number of families, genera and species per order.

Table 1 provides a comprehensive record of the macrofungal biota of Pakistan known to date, to the best of our knowledge. It lists 1,293 species belonging to 411 genera, 115 families and 24 orders. Out of which, 1,117 species, 338 genera, 83 families and 16 orders belong to Basidiomycota and 176 species, 73 genera, 32 families and eight orders are from Ascomycota. The source reference in Table 1 indicates that most entries are from the extensive checklist by Ahmad et al. (1997; 874 entries). The highest order recorded is Agaricales (27%) with 31 families, 146 genera and 602 species, followed by Polyporales (11%), Russulales (9%) and Pezizales (8%). The orders of least occurrences are Atheliales, Leotiales and Trechisporales representing one taxon in a single genus and family. The proportion of respective families, genera as well as species are shown in Fig. 1.

**Figure 2.**
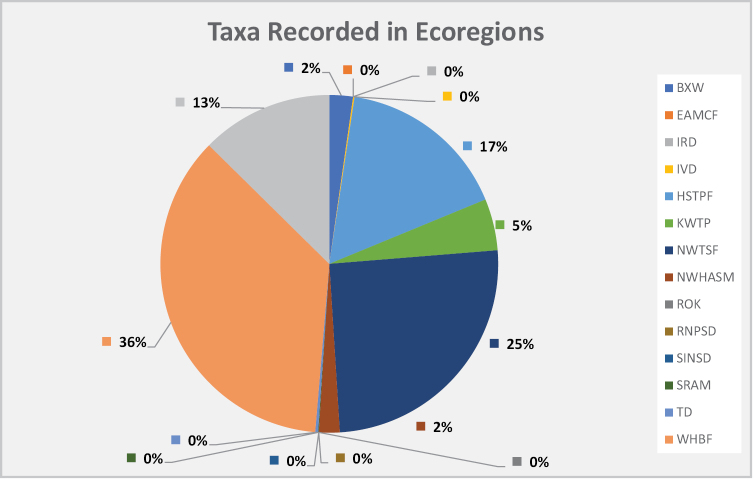
Percentage of macrofungi taxa in different ecoregions of Pakistan. Where, ***BXW*** = Baluchistan Xeric woodlands, ***EAMCF*** = East Afghan montane conifer forests, ***IRM*** = Indus River Delta Arabian Sea mangroves, ***IVD*** = Indus Valley Desert, ***HSTPF*** = Himalayan subtropical pine forests, ***KWTP*** = Karakorem West Tibetan Plateau alpine steppe, ***NWTSF*** = North-western thorn scrub forest, ***NWHASM*** = North-western Himalayan alpine scrub & meadows, ***RNPSD*** = Registan north Pakistan sandy desert, ***ROK*** = Rann of Kutch seasonal marsh, ***SINSD*** = South Iran Nubo-Sindian desert & semi-desert, ***SRAM*** = Sulaiman Range Alpine meadows, ***WHBF*** = Western Himalayan broadleaf forests, ***WHSACF*** = Western Himalayan subalpine conifer forests, ***TD*** = Thar Desert.

Table 2 indicates the ecoregions from which each genus was recorded. Fig. 2 shows that the highest macrofungal diversity is found in the western Himalayan broadleaf forests (36%) followed by north-western thorn scrub forests (25%). In addition, Himalayan subtropical pine forests have rich macrofungal diversity with 17% taxa representation, followed by western Himalayan subalpine conifer forests with 13% distribution. The Karakorem West Tibetan Plateau alpine steppe and Baluchistan xeric woodlands show 5% or lesser distribution. On the other hand, the Indus River Delta, Arabian Sea mangroves, Thar Desert, Sulaiman range alpine meadows, East Afghan montane conifer forests, Registan north Pakistan sandy desert, south Iran Nubo-Sindian desert and semi-desert, Rann of Kutch seasonal marsh, as well as the north-western Himalayan alpine scrub and meadows have 0–2% macromycetes record.

Biomes and macrofungi occurrence details therein are presented in the supplementary document labelled “Macrofungi_list_by_biomes_and_ecoregions_of_Pakistan, whereas supplementary table entitled “Detailed_compendium_of_macrofungi_of_Pakistan” contains thorough information about synonymy, locality and taxa level ecoregion allocation.

**Table 1.** A compendium of macrofungi of Pakistan. Note:FOP refers to checklist ‘Fungi of Pakistan’ (Ahmad et al. 1997).

Phylum/Order	Family	Genus	Species	Authority	Source
Basidiomycota/Agaricales	Agaricaceae	*Agaricus*	*arvensis*	Schaeff.	FOP
			*bisporiticus*	Nawaz, Callac, Thongklang & Khalid	GenBank (KJ575608); Thongklang et al. (2014)
			*bisporus*	(J.E. Lange) Imbach	GenBank (KU170542); Sultana et al. (2011)
			*bitorquis*	(Quel.) Sacc.	GenBank (KU170541); FOP
			*bolorhizus*	Berk. & Broome	FOP
			*callipelus*	Berk. & Broome	FOP
			*campestris*	L.	Razaq et al. (2014)
			*dulcidulus*	Schulzer	Razaq et al. (2014)
			*endoxanthus*	Berk. & Broome	GenBank (MK101039); FOP
			*glabriusculus*	S. Hussain	GenBank (MK751855); Hussain and Sher (2019)
			*goossensiae*	Heinem.	GenBank (KU170540)
			*gregariomyces*	J.L. Zhou & R.L. Zhao	GenBank (MK101032)
			*hemilasius*	Berk. & Broome	FOP
			*heterocystis*	Heinem. & Gooss.-Font.	GenBank (KU170543)
			*inoxydabilis*	Heinem.	GenBank (KU170539)
			*latiumbonatus*	S. Hussain	GenBank (MK751859) ; Hussain and Sher (2019)
			*lateriticolor*	Heinem.	FOP
			*latipes*	Berk.	FOP
			*pakistanicus*	H. Bashir, A.N. Khalid, L.A. Parra & Callac	GenBank (MG669256); Bashir et al. (2018)
			*placomyces*	Peck.	FOP
			*pseudopratensis*	(Bohus) Bohus	GenBank (MK123324)
			*punjabensis*	Qasim, A. Ashraf & Khalid	GenBank (KT985908); Chen et al. (2016)
			*rufoalbus*	Berk.	FOP
			*semotus*	Fr.	FOP
			*sinoplacomyces*	P. Callac & R.L. Zhao	GenBank (KY741891)
			*squalidus*	Massee	FOP
			*sparsisquamosus*	H. Bashir, S. Hussain, A.N. Khalid & H. Ahmed	GenBank (MG669253); Sultana et al. (2011); Bashir et al. (2018)
			*sylvaticus*	Schaeff.	Sultana et al. (2011)
			*trisulphuratus*	Berk.	GenBank (KU170545); FOP; Sultana et al. (2011)
			*woodrowii*	Massee	FOP
			*xanthodermus*	Genev.	GenBank (KU170544)
		*Baeospora*	*myosura*	(Fr.) Singer	FOP
		*Battarrea*	*phalloides*	(Dicks.) Pers.	FOP; Yousaf et al. (2013a)
		*Chamaemyces*	*fracidus*	(Fr.) Donk.	FOP
		*Chlorophyllum*	*hortense*	(Murrill) Vellinga	GenBank (KM350689)
			*molybdites*	(G. Mey.) Massee	GenBank (MN577080); Razaq and Shahzad (2012)
			*palaeotropicum*	Z.W. Ge & A. Jacobs	GenBank (MN577079)
			*rachodes*	(Vittad.) Vellinga	FOP; Sultana et al. (2011)
		*Coprinus*	*comatus*	(Muell. Ex Fr.) S.F. Gray	GenBank (HE819398); FOP; Razaq et al. (2014c)
			*hookeri*	Berk.	FOP
Agaricales	Agaricaceae	*Cystoderma*	*amianthinum*	(Scop.) Fayod	FOP
		*Cystodermella*	*cinnabarina*	(Alb & Schwein.) Harmaja	Razaq et al. (2013c)
			*granulosa*	(Batsch) Harmaja	FOP
		*Cystolepiota*	*pseudogranulosa*	(Berk. & Broome) Pegler	FOP
		*Disciseda*	*cervina*	(Berk) Hollos	FOP
		*Echinoderma*	*asperum*	(Pers.) Bon	FOP, Razaq et al. (2013a)
		*Hymenagaricus*	*alphitochrous*	(Berk. & Broome) Heinem.	FOP
		*Lepiota*	*albogranulosa*	Qasim & Khalid	Qasim et al. (2015b)
			*anthomyces*	(Berk. & Broome) Sacc.	FOP
			*brunneoincarnata*	Chodat & C. Martín	Razaq et al. (2013b)
			*ceramogenes*	(Berk. & Broome) Sacc.	FOP
			*cholistanensis*	H. Bashir, Usman & Khalid	Bashir et al. (2020a)
			*cingulum*	Kelderman	GenBank (MN240457)
			*clypeolaria*	(Bull.) P. Kumm.	GenBank (KJ906506)
			*cristata*	(Bolton) P. Kumm.	FOP; Razaq et al. (2013a)
			*eriphaea*	(Berk. & Br.) Sacc.	FOP
			*erythrogramma*	(Berk. & Br.) Sacc.	FOP
			*himalayensis*	Khalid & Razaq	Razaq et al. (2012a)
			*ignivolvata*	Bousset & Joss. ex Joss.	Sultana et al. (2011)
			*lahorensis*	Qasim & Khalid	GenBank (KT186609); Qasim et al. (2016)
			*lepidophora*	(Berk. & Broome) Sacc.	FOP
			*leprica*	(Berk. & Broome) Sacc.	FOP
			*magnispora*	Murrill	Sultana et al. (2011)
			*metulispora*	(Berk. & Broome) Sacc.	FOP
			*micropholis*	(Berk. & Broome) Sacc.	FOP
			*ochraceofulva*	P.D. Orton	Sultana et al. (2011)
			*pardalota*	Sacc.	FOP
			*revelata*	(Berk. & Broome) Sacc.	FOP
			*subincarnata*	J.E. Lange	FOP; Razaq et al. (2013b)
			*vellingana*	Nawaz & Khalid	Nawaz et al. (2013)
		*Leucoagaricus*	*asiaticus*	Qasim, Nawaz, & Khalid	Ge et al. (2015)
			*badhamii*	(Berk. & Broome) Singer	Sultana et al. (2011)
			*badius*	S. Hussain, Pfister, Afshan & Khalid	Hussain et al. (2018b)
			*brunneus*	Z. Ullah, Jabeen & Khalid	Ullah et al. (2019)
			*lahorensiformis*	S. Hussain, H. Ahmad, Afshan & Khalid	Hussain et al. (2018b)
			*lahorensis*	Qasim, T. Amir & Nawaz	Qasim et al. (2015a)
			*leucothites*	(Vittad.) Wasser	FOP
			*nivalis*	(W.F. Chiu) Z.W. Ge & Zhu L. Yang	GenBank (MK106148); Jabeen et al. (2020a)
			*meleagris*	(Gray) Singer	FOP
			*pabbiensis*	S. Jabeen & A.N. Khalid	GenBank (MG973423); Usman and Khalid (2018)
			*pakistaniensis*	S. Jabeen & A.N. Khalid	GenBank (KU647726); Hussain et al. (2018b)
			*serenus*	(Fr.) Bon & Boiffard	FOP; Sultana et al. (2011)
			*sultanii*	S. Hussain, H. Ahmad & Khalid	Hussain et al. (2018b)
			*umbonatus*	S. Hussain, H. Ahmad & Afshan	GenBank (KU647737); Hussain et al. (2018b)
			*viriditinctus*	(Berk. & Broome) J.F. Liang, Zhu L. Yang & J. Xu	FOP
		*Leucocoprinus*	*birnbaumii*	(Corda.) Singer	GenBank (KJ717764); FOP
Agaricales	Agaricaceae	*Leucocoprinus*	*cepaestipes*	(Sw. ex Fr.) Pat.	FOP
			*zeylanicus*	(Berk.) Boedijn	FOP
		*Macrolepiota*	*dolichaula*	(Berk & Broome) Pegler & R.W. Rayner	GenBank (KJ643334); Fiaz et al. (2014)
			*excoriata*	(Schaeff.) Wasser	GenBank (KJ643333); Fiaz et al. (2014)
			*procera*	(Scop.) Singer	FOP
			*venenata*	Bon	Sultana et al. (2011)
		*Micropsalliota*	*arginea*	(Berk. & Broome) Pegler & R.W. Rayner	FOP
			*brunneosperma*	(Berk. & Broome) Höhn.	FOP
			*plumaria*	(Berk. & Broome) Höhn.	FOP
		*Montagnea*	*arenaria*	(DC) Zeller	FOP
		*Mycenastrum*	*corium*	(Guers.) Desv.	FOP
		*Podaxis*	*pistillaris*	(L.) Fr.	FOP
		*Phellorinia*	*herculeana*	(Pers.) Kreisel	Sultana et al. (2011); Yousaf et al. (2012b)
		*Schizostoma*	*laceratum*	Ehrenb. ex Fr.) Lév.	FOP
			*mundkuri*	(S. Ahmad) Long & Stouffer	FOP
		*Tulostoma*	*ahmadii*	H. Hussain & Khalid	GenBank (KP738711); Hussain et al. (2015b)
			*amnicola*	Long & S. Ahmad	FOP
			*australianum*	Lloyd	FOP
			*balanoides*	Long & S. Ahmad	FOP
			*brumale*	Pers.	FOP
			*cineraceum*	Long	FOP
			*crassipes*	Long & S. Ahmad	FOP
			*egranulosum*	Lloyd	FOP
			*evanescens*	Long & S. Ahmad	FOP
			*exitum*	Long & S. Ahmad	FOP
			*hygrophilum*	Long & S. Ahmad	FOP
			*inonotum*	Long & S. Ahmad	FOP
			*ladhaerens*	Lloyd	FOP
			*macalpineanum*	Lloyd	FOP
			*mussooriense*	Henn.	FOP
			*occidentale*	Lloyd	FOP
			*operculatum*	Long & S. Ahmad	FOP
			*parvissimum*	Long & S. Ahmad	FOP
			*perplexum*	Long & S. Ahmad	FOP
			*pluriosteum*	Long & S. Ahmad	FOP
			*puncticulosum*	Long & S. Ahmad	FOP
			*squamosum*	(J.F. Gmel.) Pers.	GenBank (KT285883); Hussain et al. (2015b)
			*volvulatum*	I.G. Borshch.	FOP
			*vulgare*	Long & S. Ahmad	FOP
			*xerophilum*	Long	FOP
		*Xanthagaricus*	*flavidorufus*	(Berk. & Broome) Little Flower, Hosag. & T.K. Abraham	FOP
			*pakistanicus*	S. Hussain, Afshan & H. Ahmad	GenBank (KY621555); Hussain et al. (2018c)
			*subaeruginosus*	(Berk. & Broome) S. Hussain	FOP; Hussain et al. (2018c)
	Amanitaceae	*Amanita*	*ahmadii*	Jabeen, I. Ahmad, M. Kiran, J. Khan & Khalid	GenBank (MF070490); Jabeen et al. (2019)
			*battarrae*	(Boud.) Bon	Tulloss et al. (2001); Sultana et al. (2011)
Agaricales	Amanitaceae	*Amanita*	*caesarea*	(Scop.) Pers.	FOP
			*ceciliae*	(Berk. & Broome) Bas	FOP
			*cinnamomescens*	Tulloss, S.H. Iqbal, A.N. Khalid & Bhandary	Tulloss et al. (2005)
			*cinis*	S. Ullah, A.W. Wilson, Tulloss & Khalid	Ullah et al. (2019b)
			*emodotrygon*	Mehmood, Tulloss, K. Das, Hosen & R.P. Bhatt	Ullah et al. (2019b)
			*flavipes*	S. Imai.	FOP; Tulloss et al. (2001)
			*glarea*	Jabeen, M. Kiran & Sadiqullah	GenBank (KY817310); Jabeen et al. (2017c)
			*griseofusca*	J. Khan & M. Kiran	GenBank (MH241055); Kiran et al. (2018)
			*hemibapha*	(Berk. & Broome) Sacc.	FOP
			*longistriata*	S. Imai	FOP
			*mansehraensis*	M. Saba, Haelew. & A.N. Khalid	Saba et al. (2019b)
			*muscaria*	(L.) Lam.	GenBank (MK719200), FOP
			*olivovaginata*	S. Ullah, Tulloss & Khalid	Ullah et al. (2019a)
			*orsonii*	Ash. Kumar & T.N. Lakh.	GenBank (KU248132); Tulloss et al. (2001)
			*pantherina*	(DC.) Krombh	FOP; Sultana et al. (2011)
			*pakistanica*	Tulloss, S.H. Iqbal & Khalid	GenBank (KX061523); Tulloss et al. (2001)
			*pallidorosea*	P. Zhang & Zhu L. Yang	GenBank (KY621476); Kiran et al. (2017)
			*phalloides*	(Vaill. ex Fr.) Link	FOP
			*porphyria*	Alb. & Schwein.	FOP
			*pseudovaginata*	Hongo	GenBank (MT277138); Naseer and Khalid (2020a)
			*rubescens*	Pers.	FOP; Niazi et al. (2009)
			*subjunquillea*	S. Imai	GenBank (MH998627); Ishaq et al. (2019a)
			*vaginata*	(Bull.) Lam.	FOP
			*verna*	(Bull. Ex Fr.) Roques	FOP
			*virosa*	Bertill.	FOP
			*watlingii*	Kumar, BhattAsh. Kumar & T.N. Lakh.& Lakhanpal.	FOP
		*Saproamanita*	*nana*	(Singer) Redhead, Vizzini, Drehmel & Contu	FOP
		*Limacella*	*delicata*	(Fr.) Earle ex Konrad & Maubl.	FOP
		*Limacellopsis*	*guttata*	(Pers.) Zhu L. Yang, Q. Cai & Y.Y. Cui	FOP
		*Zhuliangomyces*	*pakistanicus*	Usman & Khalid	GenBank (MN240881); Usman and Khalid (2020a)
			*illinitus*	(Fr.) Redhead	FOP
	Bolbitaceae	*Bolbitius*	*titubans*	(Bull.) Fr.	FOP
		*Conocybe*	*khasiensis*	(Berk.) Watling	FOP
			*macrocephala*	Kühner & Watling	FOP
			*mesospora*	Kühner ex Watling	FOP
			*pubescens*	(Gillet) Kühner	FOP
			*punjabensis*	A. Izhar, H. Bashir & Khalid	GenBank (MK637515); Izhar et al. (2019)
			*rickenii*	(Jul. Schäff.) Kühner	FOP
			*semiglobata*	Kühner & Watling	FOP
			*semiglobatavar.campanulata*	Hauskn.	GenBank (MT994769)
			*tenera*	(Schaeff.) Kühner	FOP
Agaricales	Bolbitaceae	*Descolea*	*flavoannulata*	(Lj.N. Vassiljeva) E. Horak	Niazi et al. (2007)
			*quercina*	J. Khan & Naseer	GenBank (MF966634); Khan et al. (2017a)
	Callistosporiaceae	*Callistosporium*	*luteo-olivaceum*	(Berk. & M.A. Curtis) Singer	GenBank (KJ101607); Saba and Khalid (2014a)
		*Macrocybe*	*gigantea*	(Massee) Pegler & Lodge	GenBank (LK932287); Razaq et al. 2016b
		*Pseudolaccaria*	*pachyphylla*	(Fr.) Vizzini & Contu	GenBank (KJ906503)
	Clavariaceae	*Clavaria*	*rosea*	Fr.	FOP
			*vermicularis*	Batsch	FOP
		*Clavulinopsis*	*corniculata*	(Schaeff.) Corner	FOP
	Cortinariaceae	*Cortinarius*	*acetosus*	(Velen.) Melot	Razaq et al. (2014)
			*brunneocarpus*	Razaq & Khalid	GenBank (MN738695); Song et al. (2019)
			*bulliardii*	(Pers.) Fr.	FOP
			*cinnamomeus*	(L.) Gray	FOP
			*claricolor*	(Fr.) Fr.	Sultana et al. (2011)
			*delibutus*	Fr.	Sultana et al. (2011)
			*elegantissimus*	Rob. Henry	Sultana et al. (2011)
			*gentilis*	(Fr.) Fr.	Sultana et al. (2011)
			*hinnuleus*	Fr.	FOP
			*longistipitatus*	M. Saba, S. Jabeen, Khalid & Dima	GenBank (MF872641); Saba et al. (2017)
			*leucopus*	(Bull.) Fr.	GenBank (JN133921)
			*melanotus*	Kalchbr.	Sultana et al. (2011)
			*olivaceofuscus*	Kühner	Sultana et al. (2011)
			*pakistanicus*	A. Naseer & A. N. Khalid	Naseer et al. (2020b)
			*percomis*	Fr.	Sultana et al. (2011)
			*pseudotorvus*	A. Naseer, J. Khan & A.N. Khalid	GenBank (MN864286); Naseer et al. (2020b)
			*purpureus*	(Bull.) Bidaud, Moënne-Locc. & Reumaux	FOP
			*rufo-olivaceus*	(Pers.) Fr.	Sultana et al. (2011)
			*sanguineus*	(Wulfen) Gray	Sultana et al. (2011)
			*subturbinatus*	Rob. Henry	Sultana et al. (2011)
			*violaceus*	(L.) Gray	Sultana et al. (2011)
	Crepidotaceae	*Crepidotus*	*applanatus*	(Pers.) P. Kumm.	FOP
			*caspari*	Velen.	FOP
			*epibryus*	(Fr.) Quel.	FOP
			*mollis*	(Schaeff.) Staude	FOP
		*Simocybe*	*centunculus*	(Fr.) P. Karst.	Razaq and Shahzad (2017)
	Cyphellaceae	*Chondrostereum*	*purpureum*	(Pers.) Pouzar	FOP
	Entolomataceae	*Clitocella*	*mundula*	(Lasch) Kluting, T.J. Baroni & Bergemann	FOP
			*popinalis*	(Fr.) Kluting, T.J. Baroni & Bergemann	Sultana et al. (2011)
		*Clitopilus*	*apalus*	(Berk. & Broome) Petch	FOP
			*hobsonii*	(Berk.) P.D. Orton	FOP
			*peri*	(Berk. & Broome) Petch	FOP
			*pinsitus*	(Fr.) Joss.	FOP
			*scyphoides*	(Fr.) Singer	Sultana et al. (2011)
		*Entoloma*	*cetratum*	(Fr.) M.M. Moser	Sultana et al. (2011)
			*gnophodes*	Berk. & Broome) E. Horak	FOP
			*gnaphalodes*	(Berk. & Broome) E. Horak	FOP
			*incanum*	(Fr.) Hesler	FOP
			*iodnephes*	(Berk. & Broome) Pegler	FOP
			*mougeotii*	(Fr.) Hesler	Sultana et al. (2011)
Agaricales	Entolomataceae	*Entoloma*	*papillatum*	(Bres.) Dennis	Sultana et al. (2011)
			*polycolor*	Blanco-Dios	FOP
			*shandongense*	T. Bau & J.R. Wang	GenBank (MT255022); Haelewaters et al. (2020)
		*Leptonia*	*gnaphodes*	(Berk. & Broome) Sacc.	FOP
		*Rhodocybe*	*truncata*	(Schaeff.) Singer	Sultana et al. (2011)
			*subgilva*	(Berk. & Broome) Pegler	FOP
	Hygrophoraceae	*Arrhenia*	*epichysium*	(Pers.) Redhead, Lutzoni, Moncalvo & Vilgalys	FOP
		*Hygrocybe*	*acutoconica*	(Clem.) Singer	Sultana et al. (2011)
			*bresadolae*	Quel.	FOP
			*chlorophana*	(Fr.) Wunsch	FOP
			*conica*	(Schaeff.) P. Kumm.	FOP; Sultana et al. (2011)
			*nigrescens*	(Quél.) Kühner	Sultana et al. (2011)
			*ovina*	(Bull.) Kühner	Sultana et al. (2011)
			*spadicea*	(Scop.) P. Karst. [as 'Hydrocybe']	Sultana et al. (2011)
		*Hygrophorus*	*alboflavescens*	A. Naseer & A.N Khalid	GenBank (MK066232); Naseer et al. (2019b)
			*agathomus*	Fr. (Fr.)	FOP
			*chrysodon*	(Batsch) Fr.	Razaq et al. (2014b)
			*marzuolus*	(Fr.) Bres.	Razaq and Shahzad (2005a)
			*pudorinus*	(Fr.) Fr.	GenBank (MK066233); Naseer et al. (2019b)
			*scabrellus*	A. Naseer & A.N Khalid	Genbak (MK066234); Naseer et al. (2019b)
	Hydnangiaceae	*Laccaria*	*amethystina*	Cooke	FOP
			*bicolor*	(Maire) P.D. Orton	Sultana et al. (2011)
			*glioderma*	(Fr.) Maire	FOP
			*laccata*	(Scop.) Cooke	Sultana et al. (2011)
			*ohiensis*	(Mont.) Singer	Sultana et al. (2011)
			*tortilis*	(Bolton) Cooke	Sultana et al. (2011)
	Hymenogastraceae	*Galerina*	*marginata*	(Batsch) Kühner	FOP
		*Gymnopilus*	*aeruginosus*	(Peck.) Singer	FOP
			*chrysimyces*	(Berk.) Manjula	FOP
			*chrysomyces*	(Berk.) Pegler.	FOP
			*chrysites*	(Berk.) Singer	FOP
			*dunensis*	H. Bashir, Jabeen & Khalid	GenBank (MK088247); Bashir et al. (2020b)
			*holocrocinus*	(Berk.) Singer	FOP
			*hybridus*	(Gillet) Maire	FOP
			*junonius*	(Fr.) P.D. Orton	FOP
			*lepidotus*	Hesler	GenBank (MK584298); Bashir et al. (2018)
			*penetrans*	(Fr.) Murrill.	GenBank (MF136815); Khan et al. (2017b)
			*sapineus*	(Fr.) Murrill	FOP
			*swaticus*	J. Khan, Sher & Khalid	GenBank (MF149864); Khan et al. (2017b)
		*Hebeloma*	*anthracophilum*	Maire	Sultana et al. (2011)
			*atrocoerulea*	(Fr.) Singer.	FOP
			*aff. Lutense*		GenBank
			*mesophaeum*	(Pers.) Quél.	FOP; Razaq et al. (2017)
			*pusillum*	J.E. Lange	FOP
			*sinapizans*	(Paulet) Gillet	Sultana et al. (2011)
			*theobrominum*	Quadr.	Razaq et al. (2017)
		*Naucoria*	*bohemica*	Velen.	Sultana et al. (2011)
			*conicopapillata*	(Henn.) Sacc. & P. Syd.	FOP
Agaricales	Hymenogastraceae	*Naucoria*	*salicis*	P.D. Orton	FOP
		*Phaeocollybia*	*pakistanica*	J. Khan, Sher & Khalid	GenBank (KY007615); Khan et al. (2016a)
		*Psilocybe*	*coronilla*	(Bull.) Noordel.	FOP
			*semilanceata*	(Fr.) P. Kumm.	Sultana et al. (2011)
	Inocybaceae	*Inocybe*	*aff. amblyspora*	Kühner	GenBank (HG796912)
			*aff. cryptocystis*	D.E. Stuntz	GenBank (HG796963)
			*aff. glabripes*	Ricken	GenBank (HG796964)
			*aff. hirtella*	Bres.	GenBank (HG796965)
			*aff. nitidiuscula*	(Britzelm.) Lapl.	GenBank (HG796966)
			*ahmadii*	Farooqi, Niazi & Khalid	GenBank (KX254462); Farooqi et al. (2017)
			*amblyospora*	Kühner	GenBank (KX254462)
			*amicta*	Kokkonen & Vauras	GenBank (KJ686344); Saba et al. (2015)
			*argillacea*	(Pers.) Singer	FOP
			*asterospora*	Quel.	FOP; Sultana et al. (2011)
			*caroticolor*	T. Bau & Y. G. Fan	GenBank (MH473144); Naseer et al. (2019c)
			*cryptocystis*	D.E. Stuntz	GenBank (KF679812)
			*dulcamara*	(Pers.) P. Kumm.	FOP
			*fibrosa*	(Sowerby) Gillet	Sultana et al. (2011)
			*flocculosa*	Sacc.	FOP
			*fuscidula*	Velen.	Sultana et al. (2011)
			*glabripes*	Ricken	FOP; Sultana et al. (2011)
			*geophylla*	P. Kumm.	FOP; Sultana et al. (2011); Razaq and Shahzad (2017)
			*hirtella*	Bres.	Sultana et al. (2011)
			*inocybium*	NA	FOP
			*kohistanensis*	Jabeen, I. Ahmad & Khalid	GenBank (KP316243); Jabeen et al. (2016a)
			*leptocystis*	G.F. Atk	GenBank (KX254461); Farooqi et al. (2017)
			*napipes*	J.E. Lange	Sultana et al. (2011); Razaq and Shahzad (2017)
			*nitidiuscula*	(Britzelm.) Lapl.	GenBank (HE862959); Ilyas et al. (2013a)
			*oblectabilis*	(Britz.) Sacc.	FOP
			*posterula*	(Britzelm.) Sacc.	FOP
			*praetervisa*	Quél.	Sultana et al. (2011)
			*pyriodora*	(Pers.) P. Kumm.	FOP
			*shawarensis*	A. Naseer & A.N. Khalid	GenBank (KY616964); Naseer et al. (2017b)
			*vaccina*	Kühner	Sultana et al. (2011)
		*Inosperma*	*adaequatum*	(Britzelm.) Matheny & Esteve-Raventos	Sultana et al. (2011)
			*bongardii*	(Weinm.) Matheny & Esteve-Rav.	FOP
			*erubescens*	(A. Blytt) Matheny & Esteve-Rav.	FOP; Sultana et al. (2011)
		*Mallocybe*	*agardhii*	(N. Lund) Matheny & Esteve-Rav.	Razaq and Shahzad (2017)
			*velutina*	Saba & Khalid	Saba and Khalid (2020)
		*Pseudosperma*	*brunneoumbonatum*	Saba & Khalid	GenBank (MG742419); Saba et al. (2020b)
			*flavorimosum*	Jabeen & Khalid	GenBank (MG495391); Jabeen and Khalid (2020)
			*himalayense*	(Razaq, Khalid & Kobayashi) Matheny & Esteve-Rav.	GenBank (MH745140); Liu et al. (2018)
Agaricales	Inocybaceae	*Pseudosperma*	*mimicum*	(Massee) Matheny & Esteve-Rav	GenBank (KJ546158); Saba et al. (2015)
			*pakistanense*	(Z. Ullah, S. Jabeen, H. Ahmad & A.N. Khalid) Matheny & Esteve-Rav	GenBank (MF588965); FOP; Ullah et al. (2018)
			*rimosum*	(Bull.) Matheny & Esteve-Rav.	FOP; Sultana et al. (2011)
			*squamatum*	(J.E. Lange) Matheny & Esteve-Rav.	FOP
		*Langermannia*	*wahlbergii*	(Fr.) Dring	FOP
	Lycoperdaceae	*Apioperdon*	*pyriforme*	(Schaeff.) Vizzini	FOP
		*Bovista*	*bovistoides*	(Cooke & Massee) S. Ahmad	FOP
			*concinna*	S. Ahmad	FOP
			*himalaica*	Yousaf, Krieisel & Khalid	GenBank (JN411938); Yousaf et al. (2012a)
			*longispora*	Kreisel	FOP
			*lycoperdoides*	(Cooke) S. Ahmad	FOP
			*plumbea*	Pers.	GenBank (JX183694); Yousaf et al. (2014)
			*polymorpha*	Kreisel	FOP
			*pusilla*	(Batsch) Pers.	FOP
			*trachyspora*	(Lloyd) Kreisel	FOP
		*Bovistella*	*japonica*	Lloyd	Yousaf et al. (2012b)
		*Bryoperdon*	*acuminatum*	(Bosc) Vizzini	FOP
		*Calvatia*	*ahmadii*	Khalid & S.H. Iqbal	Khalid and Iqbal (2004)
			*craniiformis*	(Schwein.) Fr.	FOP
			*cyathiformis*	(Bose) Morgan	FOP
			*fragilis*	(Quél.) Morgan	GenBank (AJ486958)
			*lilacina*	(Mont. & Berk.) Henn.	Genbank (MN544913); Haelewaters et al. (2020)
		*Lycoperdon*	*atropurpureum*	Vittad.	FOP
			*curtisii*	Berk.	GenBank (MK414502)
			*echinella*	(Pat.) S. Ahmad	FOP
			*excipuliforme*	(Scop.) Pers.	FOP; Yousaf et al. (2012b)
			*glabrescens*	Berk.	FOP
			*lahorense*	N. Yousaf & A.N. Khalid	GenBank (MK414506); Yuan et al. (2020)
			*molle*	Pers.	Razaq and Shahzad (2005b)
			*perlatum*	Pers.	FOP
			*pratense*	Pers.	GenBank (MK414499); FOP
			*pseudocurtisii*	N. Yousaf & A.N. Khalid	GenBank (MK414505); Yuan et al. 2020
			*rimulatum*	Peck	FOP
			*setiferum*	Demoulin	FOP
			*subterrania*	Ahmad	FOP
			*umbrinum*	Pers.	FOP
	Lyophyllaceae	*Hypsizygus*	*marmoreus*	(Peck) H.E. Bigelow	FOP
		*Lyophyllum*	*decastes*	(Fr.) Singer	FOP
			*nigrescens*	Hongo	FOP
		*Sagaranella*	*tesquorum*	(Fr.) V. Hofst., Clémençon, Moncalvo & Redhead	FOP
		*Tephrocybe*	*anthracophila*	(Lasch) P.D. Orton	FOP
			*aff. platypus*	(Kühner) M.M. Moser	GenBank (KY947353)
		*Termitomyces*	*acriumbonatus*	Usman & Khalid	GenBank (MT179690); Usman and Khalid (2020b)
			*clypeatus*	R. Heim,	FOP
Agaricales	Lyophyllaceae	*Termitomyces*	*eurrhizus*	(Berk.) R. Heim	FOP
			*furfuracea*	(Fr.) Gillet	FOP
			*le-testui*	(Pat.) R. Heim	FOP
			*microcarpus*	(Berk. & Broome) R. Heim	FOP; Sultana et al. (2011); Sultana et al. (2014)
			*rabuorii*	Otieno	Sultana et al. (2011)
			*sheikhupurensis*	Izhar, Khalid & H. Bashir	Izhar et al. (2020)
			*striatus*	(Beeli) Heim	FOP
			*umkowaan*	(Cooke & Massee) D.A. Reid	GenBank (KJ703245); Hussain et al. (2015c)
	Macrocystidiaceae	*Macrocystidia*	*cucumis*	(Pers.) Joss.	FOP
	Marasmiaceae	*Chaetocalathus*	*niduliformis*	(Murrill) Singer	FOP
		*Collybiopsis*	*biformis*	(Peck) R.H. Petersen	GenBank (MT162681)
			*diminuta*	(Berk. & Broome) R.H. Petersen	FOP
			*peronata*	(Bolton) R.H. Petersen	Sultana et al. (2011)
		*Crinipellis*	*rubiginosa*	Pat.	FOP; Sultana et al. (2011)
			*scabella*	(Alb. & Schwein.) Murrill	FOP; Sultana et al. (2011)
		*Marasmius*	*atrorubens*	(Berk.) Mont.	FOP
			*corrugatiformis*	Singer	FOP
			*ferrugineus*	Berk. & Broome	FOP
			*graminum*	(Lib.) Berk.	FOP
			*griseoviolaceus*	Petch	FOP
			*haematocephalus*	(Mont.) Fr.	FOP
			*ochropus*	Singer	FOP
			*oreades*	(Bolton) Fr.	GenBank (HF546217); Razaq et al. (2013d)
			*palmivorus*	Sharples	GenBank (MN559682)
			*pulcherripes*	Peck	FOP
			*rotula*	(Scop.) Fr.	FOP
			*ruforotula*	Singer	FOP
			*tubulatus*	Petch	FOP
	Mycenaceae	*Mycena*	*epipterygia*	(Scop.) Gray	FOP
			*galericulata*	(Scop.) Gray	FOP
			*haematopus*	(Pers.) P. Kumm.	FOP
			*inclinata*	(Fr.) Quél.	Sultana et al. (2011)
			*leptocephala*	(Pers.) Gillet	Sultana et al. (2011)
			*metata*	(Fr.) P. Kumm.	FOP
			*pura*	(Pers.) P. Kumm.	FOP; Razaq et al. (2014)
		*Panellus*	*stipticus*	(Bull.) P. Karst	FOP
		*Xeromphalina*	*tenuipes*	(Schwein.) A.H. Sm.	FOP
	Mythicomycetaceae	*Mythicomyces*	*corneipes*	(Fr.) Redhead & A.H. Sm.	GenBank (KY648897)
	Niaceae	*Merismodes*	*anomala*	(Pers.) Singer	FOP
	Omphalotaceae	*Anthracophyllum*	*nigritum*	(Lév.) Kalchbr.	FOP
		*Gymnopus*	*androsaceus*	(L.) Della Magg. & Trassin.	Sultana et al. (2011)
			*barbipes*	R.H. Petersen & K.W. Hughes	GenBank (MK450334); Saba et al. (2020a)
			*dryophilus*	(Bull.) Murrill	FOP; Sultana et al. (2011)
			*dysodes*	(Halling) Halling	GenBank (MT114698); Saba and Khalid (2020c)
			*erythropus*	(Pers.) Antonín, Halling & Noordel.	Sultana et al. (2011)
			*fusipes*	(Bull.) Gray	FOP; Sultana et al. (2011)
			*hirtellus*	(Berk. & Broome) Desjardin & B.A. Perry	FOP
			*ocior*	(Pers.) Antonín & Noordel.	GenBank (MK122769)
			*subnudus*	Ellis ex Peck) Halling	GenBank (MK307636)
Agaricales	Omphalotaceae	*Omphalotus*	*olearius*	(DC.) Singer	FOP; Razaq and Shahzad (2017)
		*Marasmiellus*	*biformis*	(Peck) J.S. Oliveira	Oliveira et al. (2019)
			*candidus*	(Fr.) Singer	GenBank (KJ906507); FOP
			*confluens*	(Pers.) J.S. Oliveira	FOP; Sultana et al. (2011)
			*inoderma*	(Berk.) Singer ex Furneaux	FOP
			*longistipes*	Muh. Ali, Niazi & Khalid	Haelewaters et al. (2020)
			*luxurians*	(Peck) J.S. Oliveira	GenBank (KF803761); Saba and Khalid (2014c)
			*menehune*	(Desjardin, Halling & Hemmes) J.S. Oliveira.	GenBank (KF803762); Saba and Khalid (2014c)
			*ramealis*	(Bull.) Singer	FOP; Sultana et al. (2011)
			*subnudus*	(Ellis ex Peck) J.S. Oliveira.	Oliveira et al. (2019)
		*Mycetinis*	*alliaceus*	(Jacq.) Earle ex A.W. Wilson & Desjardin	Sultana et al. (2011)
			*scorodonius*	(Fr.) A.W. Wilson & Desjardin	Sultana et al. (2011)
		*Rhodocollybia*	*butyracea*	(Bull.) Lennox	FOP
			*maculata*	(Alb. & Schwein.) Singer	FOP; Sultana et al. (2011)
			*prolixa*	(Fr.) Antonín & Noordel	Sultana et al. (2011)
			*utrorensis*	A. Sattar, M. Kiran & Khalid	GenBank (MH220536); Sattar et al. (2018)
	Physalacriaceae	*Armillaria*	*mellea*	(Vahl) P. Kumm	Sultana and Rizwana (2007)
			*omnituens*	(Berk.) Sacc.	FOP
		*Armillariella*	*mellea*	(Vahl) P. Karst	FOP; Sultana et al. (2011)
			*vara*	(Berk.) Sacc.	FOP
		*Desarmillaria*	*tabescens*	(Scop.) R.A. Koch & Aime	Sultana et al. (2011)
		*Flammulina*	*phlegmatica*	(Berk.) Sacc.	FOP
			*velutipes*	(Curtis) Singer	FOP
			*yunnanensis*	Z.W. Ge & Zhu L. Yang	GenBank (MN388767)
		*Hymenopellis*	*radicata*	(Relhan) R.H. Petersen	FOP; Sultana et al. (2011)
		*Strobilurus*	*esculentus*	(Wulfen) Singer	Sultana et al. (2011)
			*tenacellus*	(Pers.) Singer	GenBank (KY070339)
		*Xerula*	*pudens*	(Pers.) Singer	FOP; Sultana et al. (2011)
			*strigosa*	Zhu L. Yang, L. Wang & G.M. Muell.	GenBank (LK932286)
	Pluteaceae	*Pluteus*	*ephebeus*	(Fr.) Gillet	FOP
			*escharites*	(Berk. & Broome) Sacc.	FOP
			*fusconigricans*	(Berk. & Broome) Sacc.	FOP
			*laeticeps*		FOP
			*leoninus*	(Schaeff.) P. Kumm.	FOP
			*palumbinus*	(Berk.) Sacc.	FOP
			*pellitus*	(Pers.) P. Kumm.	FOP
			*petasatus*	(Fr.) Gillet	FOP
			*pulverulentus*	Murrill	FOP
			*squamosa*	(Pers. ex Fr.) Kummer	FOP
			*variabilicolor*	Babos	GenBank
		*Volvariella*	*bingensis*	(Beeli) Shaffer	Sultana et al. (2014)
			*castanea*	(Massee) G.C. Rath	FOP
			*media*	(Schumach.) Singer	FOP
			*pusilla*	(Pers.) Singer	FOP
			*taylorii*	(Berk. & Broome) Singer	FOP
			*woodrowiana*	(Massee) Manjula	FOP
		*Volvopluteus*	*earlei*	(Murrill) Vizzini, Contu & Justo	GenBank (MT353644)
			*gloiocephalus*	(DC.) Vizzini, Contu & Justo	FOP; Sultana et al. (2011)
	Pleurotaceae	*Acanthocystis*	*gemmellari*	Inzenga) Konrad & Maubl	FOP
Agaricales	Pleurotaceae	*Hohenbuehelia*	*atrocaerulea*	(Fr.) Singer	FOP
			*petaloides*	(Bull.) Schulzer	FOP
			*reniformis*	(G. Mey.) Singer	FOP
			*testudo*	(Berk.) Pegler	FOP
		*Nothopanus*	*candidissimus*	(Sacc.) Kühner	FOP
		*Pleurotus*	*atricapillus*	(Batsch.) Singer	FOP
			*cystidiosus*	O.K. Mill.	GenBank (KR149589); Hussain et al. (2015a)
			*djamor*	(Rumph. ex Fr.) Boedijn	GenBank (KX056435)
			*dryinus*	(Pers.) P. Kumm.	Sultana et al. (2011)
			*flabellatus*	Sacc.	FOP
			*membranaceus*	Massee	FOP
			*nebrodensis*	(Inzenga) Quél.	FOP
			*ostreatus*	(Jacq.) P. Kumm.	FOP
		*Resupinatus*	*applicatus*	(Batsch) Gray	FOP
			*poriaeformis*	(Pers.) Thorn, Moncalvo & Redhead	FOP
	Porotheleaceae	*Phloeomana*	*speirea*	(Fr.) Redhead	Sultana et al. (2011)
	Psathyrellaceae	*Britzelmayria*	*multipedata*	(Peck) D. Wächt. & A. Melzer	FOP
		*Coprinellus*	*campanulatus*	S. Hussain & H. Ahmad	Hussain et al. (2018a)
			*disseminatisimilis*	S. Hussain	Hussain et al. (2018a)
			*disseminatus*	(Pers.) J.E. Lange	FOP; Razaq et al. (2014)
			*marculentus*	(Britzelm.) Redhead, Vilgalys & Moncalvo	FOP
			*micaceus*	(Bull.) Vilgalys, Hopple & Jacq. Johnson	FOP
			*ovatus*	M. Kamran & S. Jabeen	Kamran and Jabeen (2020)
			*radians*	(Desm.) Vilgalys, Hopple & Jacq. Johnson	FOP; Sultana et al (2014)
			*tenuis*	S. Hussain	Hussain et al. (2018a)
		*Coprinopsis*	*atramentaria*	(Bull.) Redhead, Vilgalys & Moncalvo	GenBank (KM977767); FOP; Sultana et al. (2011)
			*cinerea*	(Schaeff.) Redhead, Vilgalys & Moncalvo	Razaq et al. (2014)
			*lagopus*	(Fr.) Redhead, Vilgalys & Moncalvo	FOP; Sultana et al. (2011)
			*lagopides*	(P. Karst.) Redhead, Vilgalys & Moncalvo	FOP
			*macropus*	(Berk. & Broome) Redhead, Vilgalys & Moncalvo	FOP
			*patouillardii*	(Quél.) Gminder	FOP; Sultana et al. (2011)
		*Homophron*	*spadiceum*	(P. Kumm.) Örstadius & E. Larss.	FOP
		*Parasola*	*auricoma*	(Pat.) Redhead Vilgalys & Hopple.	GenBank (KY461721); FOP; Hussain et al. (2018d)
			*conopilea*	(Fr.) A. Pearson & Dennis	Sultana et al. (2011)
			*glabra*	S. Hussain, Afshan, H. Ahmad & Khalid	GenBank (KY621805); Hussain et al. (2018d)
			*lilatincta*	(Bender & Uljé), Redhead, & Hopple	GenBank (KP886462); Hussain et al. (2016)
			*malakandensis*	S. Hussain, Afshan & H. Ahmad	GenBank (KP738713); Hussain et al. (2017)
			*plicatilis*	(Curtis) Redhead, Vilgalys & Hopple	FOP
			*pseudolactea*	Sadiqullah, S. Hussain & Khalid	GenBank (KY621799); Hussain et al. (2018d)
			*schroeteri*	(P. Karst.) Redhead, Vilgalys & Hopple	GenBank (KY461722)
Agaricales	Psathyrellaceae	*Parasola*	*setulosa*	(Berk. & Broome) Redhead, Vilgalys & Hopple	FOP; Sultana et al. (2011)
		*Psathyrella*	*atomata*	(Fr.) Quél.	Sultana et al. (2011)
			*ammophila*	(Durieu & Lév.) P.D. Orton	Sultana et al. (2011)
			*artemisiae*	(Pass.) Konrad & Maubl.	Sultana et al. (2011)
			*bipellis*	Quél.) A.H. Sm.	Sultana et al. (2011)
			*candolleana*	(Fr.) Maire	GenBank (KJ917666); FOP; Sultana et al. (2011)
			*corrugis*	(Pers.) Konrad & Maubl.	FOP; Sultana et al. (2011)
			*efflorescens*	(Sacc.) Pegler	FOP
			*flavogrisea*	(Berk.) Pegler	FOP
			*hirta*	Peck	Sultana et al. (2011)
			*nana*	(Massee) Manjula	FOP
			*piluliformis*	(Bull.) P.D. Orton	FOP
			*spadiceogrisea*	(Schaeff.) Maire	FOP
			*spintrigera*	(Fr.) Konr & Maubl.	FOP
		*Punjabia*	*pakistanica*	(Usman & Khalid) D. Wächt. & A. Melzer	GenBank (MH366737); Hussain et al. (2018a)
	Schizophyllaceae	*Schizophyllum*	*commune*	Fr.	GenBank (MN178555); FOP
			*radiatum*	Fr.	FOP
		*Stramatoscypha*	*fimbriata*	(Fr.) Donk.	FOP
	Strophariaceae	*Agrocybe*	*arvalis*	(Fr.) Singer	Sultana et al. (2011)
			*broadwayi*	(Murrill) Dennis	FOP
			*manihotis*	Pegler	FOP
			*pediades*	(Fr.) Fayod	GenBank (MK791714), FOP
			*stercoraria*	Pegler	FOP
			*vervacti*	(Fr.) Singer	Sultana et al. (2011)
		*Deconica*	*coprophila*	(Bull.) P. Karst.	FOP
			*merdaria*	(Fr.) Noordel.	FOP
			*montana*	(Pers.) P.D. Orton	FOP
			*pseudobullacea*	(Petch) Ram.-Cruz & Guzmán	FOP
		*Hypholoma*	*elongatum*	(Pers.) Ricken	Sultana et al. (2011)
			*fasciculare*	(Huds.) P. Kumm.	FOP; Sultana et al. (2011)
			*marginatum*	J.Schröt.	Sultana et al. (2011)
			*radicosum*	J.E. Lange	Sultana et al. (2011)
		*Kuehneromyces*	*mutabilis*	(Schaeff.) Singer & A.H. Sm.	FOP
		*Melanotus*	*proteus*	(Sacc.) Singer	FOP
		*Pholiota*	*aurivella*	(Batsch) P. Kumm.	FOP
			*gummosa*	(Lasch) Singer	GenBank (MT995199)
			*lubrica*	(Pers.) Singer	FOP
			*lucifera*	(Lasch.) Quel.	FOP
			*populnea*	(Pers.) Kuyper & Tjall.-Beuk.	FOP
			*spumosa*	(Fr.) Singer	FOP
			*squarrosa*	(Vahl) P. Kumm.	FOP
			*tuberculosa*	(Schaeff.) P. Kumm.	Sultana et al. (2011)
		*Protostropharia*	*semiglobata*	(Batsch) Redhead, Moncalvo & Vilgalys	FOP
		*Stropharia*	*aeruginosa*	(Curtis) Quél.	FOP
			*ambigua*	(Peck) Zeller	GenBank (MN957717)
			*atroferruginea*	M.B. Khan, Fiaz & A. N. Khalid	GenBank (MK141060); Khan et al. (2019)
	Tricholomataceae	*Leucopaxillus*	*paradoxus*	Costantin & L.M. Dufour Boursier [as 'paradoxa']	Sultana et al. (2011)
Agaricales	Tricholomataceae	*Leucopaxillus*	*gentianeus*	(Quel.) Kotl.	FOP, Sultana et al. (2011)
		*Tricholoma*	*aurantium*	(Schaeff.) Ricken	FOP
			*elegans*	G. Stev.	FOP
			*lascivum*	(Fr.) Gillet	FOP
			*matsutake*	(S. Ito & S. Imai) Singer	GenBank (MT448907)
			*myomyces*	(Pers.) J.E. Lange	GenBank (HF546219)
			*terreum*	(Schaeff.) P. Kumm.	FOP
			*ustale*	(Fr.) P. Kumm.	Razaq et al. (2014)
			*vaccinum*	(Schaeff.) P. Kumm.	FOP; Sultana et al. (2011)
		*Tricholomopsis*	*decora*	(Fr.) Singer	FOP
			*flammula*	Métrod ex Holec	GenBank (FR822742); Razaq et al. (2012c)
			*inamoenum*	(FR.) Gill	Sultana et al. (2011)
			*rutilans*	(Schaeff.) Singer	FOP
			*sulphurescens*	Bres.	Sultana et al. (2011)
	Tubariaceae	*Cyclocybe*	*erebia*	(Fr.) Vizzini & Matheny	GenBank (MT994783), FOP
		*Flammulaster*	*carpophilus*	(Fr.) Earle ex Vellinga	FOP
			*fulvoalbus*	(Berk. & Broome) Pegler	FOP
		*Tubaria*	*conspersa*	(Pers.) Fayod	FOP
			*furfuracea*	(Pers.) Gillet	FOP
	Typhulaceae	*Pistillaria*	*filiformis*	Corner	FOP
		*Clitocybe*	*dealbata*	(Sowerby) P. Kumm.	FOP
			*fragrans*	(With.) P. Kumm.	Sultana et al. (2014)
			*infundibuliformis*	(Schaeff.) Quél.	FOP; Sultana et al. (2011)
			*metachroa*	(Fr.) P. Kumm	Sultana et al. (2011)
			*nebularis*	(Batsch) P. Kumm.	Sultana et al. (2011)
			*squamulosa*	(Pers.) P. Kumm.	Sultana et al. (2011)
			*vibecina*	(Fr.) Quél.	FOP
		*Clitocybula*	*lacerata*	(Scop.) Metrod	FOP
		*Crucibulum*	*laeve*	(Huds.) Kambly	FOP
		*Collybia*	*macra*	Sacc.	FOP
			*reineckeana*	Henn.	FOP
			*triplicata*	(Berk.) Sacc.	FOP
		*Cyathus*	*limbatus*	Tul. & C. Tul.	FOP
			*olla*	(Batsch) Pers.	GenBank (MH593250)
			*stercoreus*	(Schwein.) De Toni	FOP
		*Fistulina*	*hepatica*	(Schaeff.) With.	FOP
		*Infundibulicybe*	*gibba*	(Pers.) Harmaja	GenBank (MT994778); FOP
			*kotanensis*	M Ishaq, Fiaz & A.N. Khalid	GenBank (MN017278); Ishaq et al. (2019b)
			*macrospora*	M. Ali, J. Khan, Niazi & Khalid	GenBank (MT548910); Ali et al. (2020)
		*Lactocollybia*	*epia*	(Berk. & Broome) Pegler	FOP
			*variicystis*	D.A. Reid & Eicker	GenBank (MN250288)
		*Lepista*	*caffrorum*	(Kalchbr. & McOwen) Singer	FOP
			*irina*	(Fr.) H.E. Bigelow	GenBank (KJ194172); FOP
			*nuda*	(Bull.) Cooke	Sultana et al. (2011)
			*sordida*	(Schumach.) Singer	FOP
		*Megacollybia*	*platyphylla*	(Pers.) Kotl. & Pouzar	FOP; Sultana et al. (2011)
		*Melanoleuca*	*cinereifolia*	(Bon) Bon	GenBank (KJ182965); Saba and Khalid (2014b)
			*dirensis*	F. Nawaz, Jabeen & Khalid	GenBank (KU556797); Nawaz et al. (2017)
			*excissa*	(Fr.) Singer	FOP
			*graminicola*	Kühner & Maire	Genbank (KX908113); Nawaz et al. (2017)
Agaricales	Typhulaceae	*Melanoleuca*	*kashmirensis*	R. Khurshed, Z. Ullah, Jabeen, H. Ahmad & Khalid	GenBank (MK541789); Ullah et al. (2020a)
		*Paralepista*	*flaccida*	(Sowerby) Vizzini	FOP; Razaq et al. (2014)
		*Secotium*	*acuminatum*	Mont.	FOP
		*Phaeolepiota*	*aurea*	(Matt.) Maire	FOP; Razaq et al. (2014)
		*Trogia*	*infundibuliformis*	Berk. & Broome	FOP
	Incertae sedis	*Leucocybe*	*connata*	(Schumach.) Vizzini, P. Alvarado, G. Moreno & Consiglio	GenBank (HE819396); FOP; Razaq et al. (2012b)
		*Henningsomyces*	*candidus*	(Pers.) Kuntze	FOP
		*Panaeolus*	*acuminatus*	(P. Kumm.) Quél.	Sultana et al. (2011)
			*cyanescens*	Sacc.	FOP
			*fimicola*	(Pers.) Gillet	FOP; Sultana et al. (2011)
			*papilionaceus*	(Bull.) Quél.	GenBank (HE819397); Razaq et al. (2012b)
			*rickenii*	Hora.	FOP; Sultana et al. (2011)
			*semiovatus*	(Sowerby) S. Lundell & Nannf.	FOP
Amylocorticiales	Amylocorticiaceae	*Anomoloma*	*myceliosum*	(Peck) Niemelä & K.H. Larss.	FOP
		*Athelia*	*rolfsii*	(Curzi) C.C. Tu & Kimbr.	GenBank
	Incertae sedis	*Plicatura*	*crispa*	(Pers.) D.A. Reid	FOP
Atheliales	Atheliaceae	*Amphinema*	*byssoides*	(Pers.) J. Erikss	FOP
Boletales	Boletaceae	*Aureoboletus*	*gentilis*	(Quél.) Pouzar	Razaq and Shahzad (2013)
		*Boletus*	*barrowsii*	Thiers & A.H. Sm.	Niazi (2008)
			*edulis*	Bull.	FOP; Razaq and Shahzad (2013)
			*erythropus*	Krombh.	FOP
			*himalayensis*	Jabeen, Sarwar & Khalid	Sarwar et al. (2018a)
			*pakistanicus*	S. Sarwar & Khalid	GenBank (JQ178324); Sarwar and Khalid (2014)
			*reticulatus*	Schaeff.	Niazi (2008); Razaq and Shahzad (2013)
			*reticuloceps*	(M. Zang, M.S. Yuan & M.Q. Gong) Q.B. Wang & Y.J. Yao	GenBank (KJ131224)
			*subvelutipes*	Peck	FOP
		*Butyriboletus*	*appendiculatus*	(Schaeff.) D. Arora & J.L. Frank	FOP
		*Caloboletus*	*calopus*	(Pers.) Vizzini	FOP
		*Chalciporus*	*piperatus*	(Bull.) Bataille	Sultana et al. (2011); Razaq and Shahzad (2013)
		*Cyanoboletus*	*pulverulentus*	(Opat.) Gelardi, Vizzini & Simonini	FOP
		*Hortiboletus*	*kohistanensis*	A. Naseer, S. Sarwar & A.N. Khalid	GenBank (MK002767); Naseer et al. (2019a)
			*rubellus*	(Krombh.) Simonini, Vizzini & Gelardi	GenBank (KJ802928); Sarwar et al. (2016)
		*Leccinum*	*aurantiacum*	(Bull.) Gray	Razaq and Shahzad (2017); Sultana et al. (2011)
			*scabrum*	(Bull.) Gray	Sultana et al. (2011); Razaq and Shahzad (2017)
			*ustale*	(Berk.) E. Horak	FOP
			*versipelle*	(Fr. & Hök) Snell	Razaq and Shahzad (2017)
		*Leccinellum*	*crocipodium*	(Letell.) Della Magg. & Trassin.	Razaq and Shahzad (2017)
			*pseudoscabrum*	(Kallenb.) Mikšík	Razaq and Shahzad (2017)
		*Neoboletus*	*luridiformis*	(Rostk.) Gelardi, Simonini & Vizzini	GenBank (KJ802930); Sarwar et al. (2016)
Boletales	Boletaceae	*Phylloporus*	*bibulosa*	(Lloyd) Ryv.	FOP
			*brunneiceps*	N.K. Zeng, Zhu L. Yang & L.P. Tang	GenBank (KY679591); Naseer et al. (2017a)
			*rhodoxanthus*	(Schwein.) Bres.	Sultana et al. (2011)
		*Porphyrellus*	*porphyrosporus*	(Fr. & Hök) E.-J. Gilbert	FOP; Razaq and Shahzad (2017)
		*Pseudoboletus*	*parasiticus*	(Bull.) Šutara	FOP
		*Rubroboletus*	*lupinus*	(Fr.) Costanzo, Gelardi, Simonini & Vizzini	Sultana et al. (2011)
		*Strobilomyces*	*longistipitatus*	D. Chakr. K. Das & S. Adhikari	GenBank (MK518064); Ullah et al. (2019a)
			*strobilaceus*	(Scop.) Berk.	FOP
		*Tylopilus*	*felleus*	(Bull.) P. Karst	Razaq and Shahzad (2017)
			*pseudoscaber*	Secr. ex A.H. Sm. & Thiers	GenBank (KJ775785); Sarwar et al. (2014a)
			*sultanii*	S. Sarwar, Khalid & Niazi,	GenBank (KJ775786); Sarwar et al. (2014a)
		*Xanthoconium*	*separans*	(Peck) Halling & Both	Gardezi (2003); Razaq et al. (2014)
			*dryophilus*	(Thiers) N. Siegel, C.F. Schwarz & J.L. Frank	Gardezi (2003)
			*fulvus*	Sarwar, I. Ahmad & Khalid	Hernández-Restrepo et al. (2016)
		*Xerocomus*	*ferrugineus*	(Schaeff.) Alessio	FOP
			*indicus*	Singer	FOP
			*rubellus*	Quél.	Niazi (2008)
			*subtomentosus*	(L.) Quél	Sultana et al. (2011); Razaq and Shahzad (2013)
	Coniophoraceae	*Coniophora*	*arida*	(Fr.) P. Karst.	FOP
			*fusispora*	(Cooke & Ellis) Cooke	FOP
		*Gyrodontium*	*sacchari*	(Spreng.) Hjortstam	FOP
	Diplocystidiaceae	*Gomphidius*	*glutinosus*	(Schaeff.) Fr.	FOP
		*Gyrodon*	*lividus*	(Bull.) Sacc.	Razaq and Shahzad (2017)
	Gastrosporiaceae	*Astraeus*	*hygrometricus*	(Pers.) Morgan	FOP; Yousaf et al. (2014)
		*Gastrosporium*	*simplex*	Mattir.	FOP
	Gomphidiaceae	*Chroogomphus*	*helveticus*	(Singer) M.M. Moser	FOP
			*pakistanicus*	M. Kiran & A.N. Khalid	GenBank (MK509771); Kiran et al. (2020)
			*pruinosus*	M. Kiran & A.N. Khalid	GenBank (MK509769); Kiran et al. (2020)
			*roseolus*	Y.C. Li & Zhu L. Yang	GenBank (LT576117); Razaq et al. (2016a)
			*rutilus*	(Schaeff.) O.K. Mill.	FOP
	Hygrophoropsidaceae	*Leucogyrophana*	*mollusca*	(Fr.) Pouzar	FOP
			*pinastri*	(Fr.) Ginns & Weresub	FOP
		*Melanogaster*	*durissimus*	Cooke	FOP
	Rhizopogonaceae	*Rhizopogon*	*flavus*	Petch	FOP
	Sclerodermataceae	*Pisolithus*	*albus*	(Cooke & Massee) Priest	GenBank (MN295477)
			*tinctorius*	(Mont.) E. Fisch.	GenBank (KF802173); Razaq and Shahzad (2004)
		*Scleroderma*	*aff. cepa*	Pers.	GenBank (HG796946)
			*areolatum*	Ehrenb.	Yousaf et al. (2012c)
			*aurantium*	(L.) Pers.	GenBank (KF802172)
			*bovista*	Fr.	GenBank (KF802171); FOP
			*cepa*	Pers.	FOP
			*chevalieri*	Guzmán	Yousaf et al. (2012c)
			*dictyosporum*	Pat.	Yousaf et al. (2012c)
			*flavidum*	Ellis & Everh.	FOP
Boletales	Sclerodermataceae	*Scleroderma*	*sinnamariense*	Mont.	FOP
			*verrucosum*	(Bull.) Pers.	FOP
	Serpulaceae	*Serpula*	*lacrymans*	(Wulfen) J. Schröt.	GenBank (AJ557312), FOP
	Suillaceae	*Suillus*	*americanus*	(Peck) Snell	GenBank (KX213755); FOP; Sarwar et al. (2011)
			*bovinus*	(L.) Roussel	Sultana et al. (2011); Razaq and Shahzad (2016)
			*brevipes*	(Peck) Kuntze	Sarwar et al. (2011); Sarwar and Khalid (2014b)
			*collinitus*	(Fr.) Kuntze	Sultana et al. (2011); Sarwar and Khalid (2014b)
			*flavidus*	(Fr.) J. Presl	Sarwar et al. (2012); Sarwar and Khalid (2014b)
			*granulatus*	(L.) Roussel	FOP; Sarwar and Khalid (2014b)
			*grevillei*	(Klotzsch) Singer	FOP
			*himalayensis*	B. Verma & M.S. Ready	GenBank (KR056819); Sarwar et al. (2018b)
			*luteus*	(L.) Roussel	Razaq and Shahzad (2016); Sultana et al. (2011)
			*marginielevatus*	S. Sarwar, Khalid & Dentinger	GenBank (KJ361512); Sarwar et al. (2015)
			*placidus*	(Bonord.) Singer	FOP; Sultana et al. 2011
			*tomentosus*	Singer	FOP; Niazi (2008); Sarwar and Khalid (2014b)
			*triacicularis*	B. Verma & M.S. Reddy	GenBank (KM677929); Sarwar et al. (2015)
			*viscidus*	(L.) Roussel	Razaq et al. (2019)
		*Suillellus*	*luridus*	(Schaeff.) Murrill	FOP
			*queletii*	(Schulzer) Vizzini, Simonini & Gelardi	FOP
	Tapinellaceae	*Pseudomerulius*	*aureus*	(Fr.) Jülich	FOP
		*Tapinella*	*atrotomentosa*	(Batsch) Šutara	FOP
			*panuoides*	(Fr.) E.-J. Gilbert	FOP
Cantharellales	Aphelariaceae	*Aphelaria*	*ceracea*	Corner	FOP
	Hydnaceae	*Cantharellus*	*cibarius*	Fr.	FOP
		*Clavulina*	*coralloides*	(L.) J. Schröt.	FOP
			*cinerea*	(Bull.) J. Schröt.	FOP
			*cinereavar.gracilis*	Rea, Trans	FOP
			*rugosa*	(Bull.) J. Schröt	FOP
		*Craterellus*	*cinereus*	(Pers.) Pers.	GenBank (MF374488); Naseer and Khalid (2018)
		*Hydnum*	*repandum*	L.	FOP
			*rufescens*	Fr.	FOP
		*Multiclavula*	*mucida*	(Pers.) R.H. Petersen	FOP
Corticiales	Punctulariaceae	*Dendrocorticium*	*polygonioides*	(P. Karst.) Donk	FOP
	Vuilleminiaceae	*Cytidia*	*salicina*	(Fr.) Burt.	FOP
Geastrales	Geastraceae	*Geastrum*	*argenteum*	Cooke	FOP
			*clelandii*	Lloyd	FOP
			*corollinum*	(Batsch) Hollós	FOP
			*coronatum*	Schaeff. ex J. Schröt.	FOP
			*drummondii*	Berk.	FOP
			*fimbriatum*	Fr.	Razaq and Shahzad (2007)
			*lageniforme*	Vittad.	FOP
			*lageniformevar.ahmadii*	Stanck.	FOP
			*minimum*	Schwein.	FOP
			*nanumvar.nanum*	Pers.	FOP
			*panjabense*	S. Ahmad	FOP
Geastrales	Geastraceae	*Geastrum*	*rufescens*	Pers.	FOP
			*saccatum*	Fr.	FOP
			*striatum*	DC.	FOP
			*velutinum*	Morgan	FOP
			*triplex*	Jungh.	FOP
		*Myriostoma*	*coliforme*	(Dicks.) Corda	Yousaf et al. (2013b)
		*Sphaerobolus*	*ingoldii*	Geml, D.D. Davis & Geiser	GenBank (MN957515)
			*stellatus*	Tode	FOP
Gloeophyllales	*Gloeophyllaceae*	*Gloeophyllum*	*sepiarium*	(Wulfen) P. Karst.	FOP
			*striatum*	(Fr.) Murrill	FOP
			*subferrugineum*	(Berk.) Bondartsev & Singer	FOP
		*Neolentinus*	*lepideus*	(Fr.) Redhead & Ginns	FOP; Razaq et al. (2018)
Gomphales	Clavariadelphaceae	*Clavariadelphus*	*elongatus*	J. Khan, Sher & Khalid	GenBank (MG768846); Sher et al. (2018)
			*pakistanicus*	Hanif & Khalid	GenBank (HQ379937); Hanif et al. (2014)
			*pistillaris*	(L.) Donk	FOP
			*subfastigiatus*	V.L. Wells & Kempton	GenBank (JX275756); Hanif and Khalid (2013)
			*truncatus*	Donk	FOP
	Gomphaceae	*Gomphus*	*clavatus*	(Pers.) Gray	FOP
			*megasporus*	Corner	FOP
		*Phaeoclavulina*	*abietina*	(Pers.) Giachini	Nasim et al. (2008)
			*flaccida*	(Fr.) Giachini	FOP
		*Ramaria*	*aurea*	(Schaeff.) Quél.	Razaq and Shahzad (2005c)
			*botrytis*	(Pers.) Bourdot	FOP
			*flava*	(Schaeff.) Quél.	FOP
			*flavavar.flava*	(Schaeff.) Quél	FOP
			*flavescentoides*	Hanif & Khalid	GenBank (KC357769); Hanif et al. (2019)
			*formosa*	(Pers.) Quél.	Nasim et al. (2008)
			*fragillima*	(Sacc. & P. Syd.) Corner	FOP
			*moelleriana*	(Bres. & Roum.) Corner	FOP
			*pallida*	(Schaeff.) Ricken	FOP
			*soluta*	(P. Karst.) Corner	FOP
			*stricta*	(Pers.) Quél.	FOP
			*subaurantiaca*	Corner	FOP
		*Turbinellus*	*floccosus*	(Schwein.) Earle ex Giachini & Castellano	FOP
	Lentariaceae	*Lentaria*	*acuminata*	Berk.	FOP
			*micheneri*	(Berk. & M.A. Curtis) Corner	FOP
			*surculus*	(Berk.) Corner	FOP
Hymenochaetales	Hymenochaetaceae	*Aurificaria*	*indica*	(Massee) D.A. Reid	FOP
		*Coltricia*	*bambusicola*	(Henn.) D.A. Reid	FOP
			*cinnamomea*	(Jacq.) Murrill	FOP
			*perennis*	(L.) Murrill	GenBank (MN892531); FOP
		*Fomitiporia*	*punctata*	(P. Karst.) Murrill	FOP
			*robusta*	(P. Karst.) Fiasson & Niemelä	FOP
		*Fuscoporia*	*callimorpha*	(Lév.) Groposo, Log.-Leite & Góes-Neto	FOP
			*ferruginosa*	(Schrad.) Murrill	FOP
			*senex*	(Nees & Mont.) Ghob.-Nejh.	FOP
			*torulosa*	(Pers.) T. Wagner & M. Fisch.	FOP
		*Hymenochaete*	*cinnamomea*	(Pers.) Bres.	FOP
Hymenochaetales	Hymenochaetaceae	*Hymenochaete*	*cruenta*	(Pers.) Donk	FOP
			*leonina*	Berk. & M.A. Curtis	FOP
			*patelliformis*	G. Cunn.	FOP
			*rheicolor*	(Mont.) Lév.	FOP
			*rubiginosa*	(Dicks.) Lév.	FOP
			*semistupposa*	Petch	FOP
			*villosa*	(Lev) Bres.	FOP
			*xerantica*	(Berk.) S.H. He & Y.C. Dai	FOP
		*Hydnoporia*	*tabacina*	(Sowerby) Spirin, Miettinen & K.H. Larss.	FOP
		*Inocutis*	*dryophila*	(Berk.) Murrill	FOP
			*tamaricis*	(Pat.) Fiasson & Niemelä	FOP
		*Inonotus*	*cuticularis*	(Bull.) P. Karst.	FOP
			*hispidus*	(Bull.) P. Karst.	FOP
			*pachyphloeus*	(Pat.) T. Wagner & M. Fisch.	FOP
			*peristrophidis*	S. Ahmad	FOP
			*tabacinus*	(Mont.) G. Cunn.	FOP
			*tinctorius*	(Quél.) S. Ahmad	FOP
			*triqueter*	P. Karst.	FOP
		*Phellinopsis*	*conchata*	(Pers.) Y.C. Dai	FOP
		*Phellinus*	*allardii*	(Bres.) S. Ahmad	FOP
			*badius*	(Cooke) G. Cunn.	FOP
			*caryophylli*	(Racib.) G. Cunn.	FOP
			*contiguus*	(Pers. ex Fr.) Bours. & Galz.	FOP
			*fastuosus*	Lév.) S. Ahmad	FOP
			*gilvus*	(Schwein.) Pat.	FOP
			*gilvusvar.scruposus*	(Fr.) Ahmad	FOP
			*igniarius*	(L.) Quél.	FOP
			*laevigatus*	(P. Karst.) Bourdot & Galzin	FOP
			*nilgheriensis*	(Mont.) G. Cunn.	FOP
			*pectinatus*	(Klotzsch) Quél.	FOP
			*pini*	(Brot.) Pilát	FOP
			*pinivar.abietisf.microspora*	Pilat.	FOP
			*purpureagilvus*	(Petch.) Ryvarden	FOP
			*ribis*	(Schumach.) Quél.	FOP
			*ribisf.gymnosporiae*		FOP
			*ribisf.lycil*		FOP
			*rimosus*	(Berk.) Pilat.	FOP
		*Phylloporia*	*ribis*	(Schumach.) Ryvarden	FOP
			*tiliae*	L.W. Zhou	GenBank (MN080232)
			*chrysites*	(Berk.) Ryvarden	FOP
		*Porodaedalea*	*himalayensis*	(Y.C. Dai) Y.C. Dai	GenBank (MK995632)
			*indica*	Spirin, Y.C. Dai & Vlasák	GenBank (MN829552)
		*Pseudoinonotus*	*dryadeus*	(Pers.) T. Wagner & M. Fisch.	FOP
		*Onnia*	*tibetica*	Y.C. Dai & S.H. He	GenBank (MT050549)
			*tomentosa*	(Fr.) P. Karst.	FOP
		*Tropicoporus*	*linteus*	(Berk. & M.A. Curtis) L.W. Zhou & Y.C. Dai	FOP
		*Xanthoporia*	*radiata*	(Sowerby) Ţura, Zmitr., Wasser, Raats & Nevo	Razaq and Shahzad (2017)
	Hyphodontiaceae	*Hyphodontia*	*arguta*	(Fr.) J. Erikss.	FOP
			*pallidula*	(Bres.) J. Erikss.	FOP
	Oxyporaceae	*Oxyporus*	*latemarginatus*	(Durr. & Mont.)	FOP
			*populinus*	(Schumach.) Donk	FOP
Hymenochaetales	Rickenellaceae	*Peniophorella*	*pubera*	(Fr.) P. Karst.	FOP
	Schizoporaceae	*Schizopora*	*paradoxa*	(Schrad.) Donk	FOP
		*Xylodon*	*raduloides*	Riebesehl & Langer	FOP
	Incertae sedis	*Trichaptum*	*abietinum*	(Pers. ex J.F. Gmel.) Ryvarden	FOP
			*biforme*	(Fr.) Ryvarden	FOP
Hysterangiales	Phallogastraceae	*Protubera*	*clathroidea*	Dring	FOP
			*maracuja*	Möller	FOP
Phallales	Phallaceae	*Colus*	*hirudinosus*	Cavalier & Séchier	FOP
		*Itajahya*	*rosea*	(Delile) E. Fisch.	GenBank (KF481955); FOP; Moreno et al. (2013)
		*Lysurus*	*borealis*	(Burt) Henn.	FOP
			*pakistanicus*	S.H. Iqbal, Kasuya, Khalid & Niazi	Iqbal et al. (2006)
			*periphragmoides*	(Klotzsch) Dring	FOP
		*Phallus*	*calongei*	G. Moreno & Khalid	GenBank (KF481955); Moreno et al. (2009)
			*celebicus*	Henn.	FOP
			*hadriani*	Vent.	GenBank (KF481956); Moreno et al. (2013)
			*impudicus*	L.	FOP
			*indusiatus*	Vent.	FOP
			*rubicundus*	(Bose.) Fr.	FOP
Polyporales	Fomitopsidaceae	*Antrodia*	*subtrametes*	(Pilat.)	FOP
		*Brunneoporus*	*juniperinus*	(Murrill) Zmitr.	GenBank (KR610980)
		*Cellulariella*	*warnieri*	(Durieu & Mont.) Zmitr. & Malysheva	GenBank (MT491098)
		*Phaeodaedalea*	*incerta*	(Curr.) Ţura, Zmitr., Wasser & Spirin	FOP
		*Pilatoporus*	*ostreiformis*	(Berk.) Zmitr.	FOP
		*Ranadivia*	*stereoides*	(Fr.) Zmitr.	FOP
		*Resinoporia*	*crassa*	(P. Karst.) Audet	FOP
		*Rhodofomes*	*roseus*	(Alb. & Schwein.) Kotl. & Pouzar	FOP
	Dacryobolaceae	*Jahnoporus*	*oreinus*	Spirin, Vlasák & Miettinen	GenBank (MN178648)
		*Fomitopsis*	*annosavar.indicus*	(Wakef.) S. Ahmad	FOP
			*pinicola*	(Sw.) P. Karst.	FOP
			*rufolaccata*	(Bose) Dhanda	FOP
		*Postia*	*tephroleuca*	(Fr.) Jülich	FOP
	Panaceae	*Panus*	*rudis*	Fr.	FOP
	Irpicaceae	*Byssomerulius*	*corium*	(Pers.) Parmasto	FOP
		*Ceriporia*	*ferrugineocincta*	(Murrill) Ryvarden	FOP
			*leptoderma*	(Berk. & Broome) Ryvarden	FOP
			*xylostromatoides*	(Berk.) Ryvarden	FOP
		*Gloeoporus*	*thelephoroides*	(Hook.) G. Cunn.	FOP
		*Flavodon*	*flavus*	(Klotzsch) Ryvarden	GenBank (MN888947)
		*Irpex*	*flavus*	Klotzsch	FOP
			*lacteus*	(Fr.) Fr.	GenBank (KM977778)
		*Leptoporus*	*mollis*	(Pers.) Quél.	FOP
		*Trametopsis*	*cervina*	(Schwein.) Tomšovský	FOP
	Laetiporaceae	*Laetiporus*	*sulphureus*	(Bull.) Murrill	FOP; Razaq and Shahzad (2016)
		*Phaeolus*	*schweinitzii*	(Fr.) Pat.	GenBank (MN109971); FOP; Razaq and Shahzad (2016)
			*weberiana*	(Bres. & Henn. ex Sacc.) Ryv.	FOP
	Meruliaceae	*Climacodon*	*pulcherrimus*	(Berk. & M.A. Curtis) Nikol.	FOP
		*Hydnophlebia*	*chrysorhiza*	(Eaton) Parmasto	FOP
Polyporales	Meruliaceae	*Irpiciporus*	*pachyodon*	(Pers.) Kotl. & Pouzar	FOP
		*Phlebia*	*rufa*	(Pers.) M.P. Christ.	FOP
			*sedimenticola*	(S. Ahmad) S. Ahmad	FOP
			*tremellosa*	(Schrad.) Nakasone & Burds.	FOP
		*Sarcodontia*	*spumea*	(Sowerby) Spirin	FOP
		*Scopuloides*	*hydnoides*	(Cooke & Massee) Hjortstam & Ryvarden	FOP
			*leprosa*	(Bourdot & Galzin) Boidin, Lanq. & Gilles	FOP
	Meripilaceae	*Rigidoporus*	*lineatus*	(Pers.) Ryvarden	FOP
			*microporus*	(Sw.) Overeem	FOP
			*ulmarius*	(Sowerby) Imazeki	FOP; Razaq and Shahzad (2016)
			*vinctus*	(Berk.) Ryvarden	FOP
			*zonalis*	(Berk.) Imazeki	FOP
	Phanerochaetaceae	*Bjerkandera*	*adusta*	(Willd.) P. Karst.	FOP; Razaq and Shahzad (2016)
		*Aporium*	*carayae*	(Schw.) Teixiera and Rogers	FOP
		*Hyphodermella*	*corrugata*	(Fr.) J. Erikss. & Ryvarden	FOP
		*Phaeophlebiopsis*	*ravenelii*	(Cooke) Zmitr.	FOP
		*Phlebiopsis*	*gigantea*	(Fr.) Jülich	FOP
			*papyrina*	(Mont.) Boid.	FOP
		*Porostereum*	*spadiceum*	(Pers.) Hjortstam & Ryvarden	FOP
		*Rhizochaete*	*filamentosa*	(Berk. & M.A. Curtis) J. Erikss	FOP
	Podoscyphaceae	*Abortiporus*	*biennis*	(Bull.) Singer	Razaq and Shahzad (2016); Khan et al. (2016b)
		*Podoscypha*	*elegans*	(G. Mey.) Pat.	GenBank (MH858811)
			*parvula*	(Lloyd) D.A. Reid	FOP
			*petalodes*	(Berk.) Boidin	FOP; GenBank (DQ917655)
			*pusilla*	(Berk.) Ryvarden	FOP
			*warneckeana*	(Henn.) Ryvarden	FOP
	Polyporaceae	*Cerioporus*	*leptocephalus*	(Jacq.) Zmitr.	FOP
			*squamosus*	(Huds.) Quél.	GenBank (MN888950); FOP; Razaq and Shahzad (2016)
			*varius*	(Pers.) Zmitr. & Kovalenko	FOP
		*Coriolus*	*hirtellus*	(Fr.) Murrill	FOP
		*Coriolopsis*	*occidentalis*	(Klotzsch) Murrill	FOP
		*Cystostiptoporus*	*indicus*	Dhanda & Ryvarden	FOP
		*Daedalea*	*dickinsii*	Yasuda	GenBank (KR019739); FOP
			*pusillus*	(Lev.) Singer	FOP
			*quercina*	(L.) Pers.	FOP
		*Daedaleopsis*	*confragosa*	(Bolton) J. Schröt.	FOP
		*Earliella*	*scabrosa*	(Pers.) Gilb. & Ryvarden	GenBank (MN888942)
		*Epithele*	*typhae*	(Pers.) Pat.	FOP
		*Favolus*	*grammocephalus*	(Berk.) Imazeki	FOP
			*tenuiculus*	P. Beauv.	FOP
		*Fomes*	*ajazii*	S.M. Hussain	FOP
			*borneoensis*	(Lloyd) S. Ahmad	FOP
			*fomentarius*	(L.) Fr.	FOP; Razaq and Shahzad (2016)
			*semitostus*	(Berk.) Cooke	FOP
		*Funalia*	*floccosa*	(Jungh.) Zmitr. & Malysheva	FOP
			*hispida*	(Bagl.) M.M. Chen	FOP
Polyporales	Polyporaceae	*Funalia*	*leonina*	(Klotzsch) Pat.	FOP
		*Ganoderma*	*ahmadii*	Staeyart.	FOP
			*applanatum*	(Fr.) Pat.	FOP; Razaq and Shahzad (2017)
			*australe*	(Fr.) Pat.	FOP
			*flexipes*	Pat.	FOP
			*leucocontextum*	T.H. Li, W.Q. Deng, Sheng H. Wu, Dong M. Wang & H.P. Hu	GenBank (MK713839)
			*lucidum*	(Curtis) P. Karst.	GenBank (KX610998)
			*resinaceum*	Boud.	FOP
			*tornatum*	(Pers.) Bres.	FOP
		*Grammothele*	*fuligo*	(Berk. & Broome) Ryvarden	FOP
		*Hexagonia*	*discopoda*	Pat. & Har.	FOP
		*Lopharia*	*cinerascens*	(Schwein.) G. Cunn.	FOP
			*papyracea*	(Bres.) D.A. Reid	FOP
		*Lentinus*	*arcularius*	(Batsch) Zmitr.	FOP
			*brumalis*	(Pers.) Zmitr.	FOP
			*crinitus*	(L.) Fr.	FOP
			*multicolor*	Berk.	FOP
			*prolifer*	(Pat. & Har.) D.A. Reid	FOP
			*squarrosulus*	Mont.	FOP
			*tigrinus*	(Bull.) Fr.	GenBank (EU543989)
		*Lenzites*	*betulinus*	(L.) Fr.	GenBank (MN888944); FOP
			*platyphyllus*	Lev.	FOP
		*Perenniporia*	*medulla-panis*	(Jacq.) Donk	FOP
		*Picipes*	*badius*	(Pers.) Zmitr. & Kovalenko	Razaq and Shahzad (2016)
			*submelanopus*	(H.J. Xue & L.W. Zhou) J.L. Zhou & B.K. Cui	GenBank (MN888945)
		*Polyporellus*	*picipes*	(Fr.) P. Karst.	FOP
		*Polyporus*	*biennis*	(Bull.) Fr.	FOP
			*calcuttensis*	Bose	FOP
			*umbellatus*	(Pers.) Fr.	FOP; Razaq et al. (2014)
		*Poria*	*latemarginata*	(Durieu & Mont.) Cooke	FOP
			*paradoxa*	Schard. ex Fr.	FOP
		*Pyrofomes*	*demidoffi*	(Lév.) Kotl. & Pouzar	FOP
			*juniperinus*	(H. Schrenk) Vlasák & Spirin	FOP
		*Tomophagus*	*colossus*	(Fr.) Murrill	FOP
		*Trametes*	*cingulata*	Berk.	FOP
			*corrugata*	(Pers.) Bres.	FOP
			*elegans*	(Spreng.) Fr.	GenBank (MN888943); FOP
			*flavida*	(Lév.) Zmitr., Wasser & Ezhov	FOP
			*ijubarskii*	Pilat.	FOP
			*incana*	Berk.	FOP
			*lactinea*	(Berk.) Sacc.	FOP
			*ochracea*	(Pers.) Gilb. & Ryvarden	FOP
			*polyzona*	(Pers.) Justo	FOP
			*pubescens*	(Schumach.) Pilát	FOP
			*roseola*	Pat. & Har.	FOP
			*suaveolens*	(L.) Fr.	FOP
			*tephroleuca*	Berk.	FOP
			*trogii*	Berk.	FOP
			*versicolor*	(L.) Lloyd	GenBank (KU697312); Razaq et al. (2014)
Polyporales	Polyporaceae	*Truncospora*	*livida*	(Kalchbr. ex Cooke) Zmitr.	FOP
			*tephropora*	(Mont.) Zmitr.	FOP
	Incrustoporiaceae	*Tyromyces*	*chioneus*	(Fr.) P. Karst.	FOP
			*gollanii*	(Massee) S. Ahmad	FOP
	Pycnoporellaceae	*Pycnoporellus*	*fulgens*	(Fr.) Donk.	FOP
		*Pycnoporus*	*cinnabarinus*	(Jacq.) P. Karst.	FOP
		*Pycnoporus*	*sanguineus*	(L.) Murrill	FOP
	Sparassidaceae	*Sparassis*	*crispa*	Wulf. ex Fr.	FOP
			*laminosa*	Fr.	FOP
			*latifolia*	Y.C. Dai & Zheng Wang	GenBank (KF866226)
	Steccherinaceae	*Cabalodontia*	*queletii*	(Bourdot & Galzin) Piątek	FOP
		*Odontia*	*bicolor*	(Alb. & Schwein. ex Fr.) Quel.	FOP
			*calcicola*	(Bourdot & Galzin) Kõljalg	FOP
		*Antrodiella*	*oleaginea*	Overh. ex Ryvarden	FOP
		*Mycorrhaphium*	*stereoides*	(Cooke) Maas Geest.	FOP
		*Steccherinum*	*ochraceum*	(Pers. ex J.F. Gmel.) Gray	FOP
	Incertae sedis	*Amaropostia*	*stiptica*	(Pers.) B.K. Cui, L.L. Shen & Y.C. Dai	FOP
		*Hypochnicium*	*punctulatum*	(Cooke) J. Erikss.	FOP
		*Phanerodontia*	*chrysosporium*	(Burds.) Hjortstam & Ryvarden	GenBank (EU543990)
Russulales	Auriscalpiaceae	*Auriscalpium*	*vulgare*	Gray	FOP
		*Lentinellus*	*micheneri*	(Berk. & M.A. Curtis) Pegler	FOP
			*ursinus*	(Fr.) Kuhner	FOP
	Bondarzewiaceae	*Albatrellus*	*roseus*	J. Khan, Sher & Khalid	GenBank (MF110285); Khan et al. (2018)
		*Amylosporus*	*campbellii*	(Berk.) Ryvarden	FOP
			*succulentus*	Jia J. Chen & L.L. Shen	GenBank (MK929297)
		*Bondarzewia*	*dickinsii*	(Berk.) Jia J. Chen, B.K. Cui & Y.C. Dai	FOP
		*Heterobasidion*	*amyloideopsis*	Saba, C.L. Zhao, Khalid & Pfister	Genbank (KT598384); Zhao et al. (2017)
			*insulare*	(Murrill) Ryvarden	FOP
			*linzhiense*	Y.C. Dai & Korhonen	GenBank (MH233930); Saba et al. (2018)
			*orientale*	Tokuda, T. Hatt. & Y.C. Dai.	GenBank (MH233931); Saba et al. (2018)
	Hericiaceae	*Hericium*	*cirrhatum*	(Pers.) Nikol.	GenBank (MN513042); Khan et al. (2020)
			*clathroides*	(Pall.) Pers.	FOP
			*coralloides*	(Scop.) Pers.	FOP
			*erinaceus*	(Bull.) Pers.	FOP
		*Laxitextum*	*bicolor*	(Pers.) Lentz	FOP
	Peniophoraceae	*Asterostroma*	*laxum*	Bres.	FOP
		*Dichostereum*	*pallescens*	(Schwein.) Boidin & Lanq.	FOP
			*rhodosporum*	(Wakef.) Boidin & Lanq.	FOP
		*Duportella*	*velutina*	Pat.	FOP
			*tristicula*	(Berk. & Broome) Reinking	GenBank (MH858266)
		*Lachnocladium*	*fulvum*	Corner	FOP
		*Peniophora*	*cinerea*	(Pers.) Cooke	FOP
			*versiformis*	(Berk. & M.A. Curtis) Bourdot & Galzin	FOP
		*Scytinostroma*	*cystidiatum*	Boidin	FOP
			*portentosum*	(Berk.& Curt.) Donk	FOP
	Russulaceae	*Russula*	*abbottabadensis*	Saba & Adamčík	GenBank (MZ364137); Adamčík et al. (2019)
			*adusta*	(Pers.) Fr.	Sultana et al. (2011)
Russulales	Russulaceae	*Russula*	*ahmadii*	Jabeen, Razaq, Niazi, I. Ahmad & Khalid	Genbank (KU535608); Jabeen et al. (2017b)
			*amethystina*	Quél.	GenBank (KT953612)
			*amoenicolor*	Romagn.	Sultana et al. (2011)
			*anthracina*	Romagn.	GenBank (KR011879), Jabeen et al. (2016b)
			*aurea*	Pers.	FOP
			*aurantioflava*	Kiran & Khalid	GenBank (MN130074); Adamčík et al. (2019)
			*azurea*	Bres.	FOP
			*badia*	Quel.	FOP
			*brevipes*	Peck	Niazi et al. (2006)
			*brunneopurpurea*	Jabeen & Khalid	GenBank (KT953613); Jabeen et al. (2017a); Ahmad et al. (2019)
			*caerulea*	Fr.	Sultana et al. (2011)
			*cessans*	A. Pearson	GenBank (KF679816)
			*chloroides*	(Krombh.) Bres.	FOP
			*cinnabarina*	Berk.	FOP
			*consobrina*	(Fr.) Fr.	Sultana et al. (2011)
			*cyanoxantha*	(Schaeff.) Fr.	FOP; Razaq et al. (2019)
			*decipiens*	(Singer) Bon	Sultana et al. (2011)
			*delica*	Fr.	FOP
			*densifolia*	Secr. ex Gillet	FOP
			*emetica*	(Schaeff.) Pers.	FOP
			*fellea*	(Fr.) Fr.	FOP
			*foetentoides*	Razaq, Khalid & Niazi	GenBank (HE647707); Razaq et al. (2014a)
			*foetida*	C. Martin	FOP
			*grata*	Britzelm.	FOP; Razaq et al. (2019)
			*integra*	(L.) Fr.	FOP
			*livescens*	(Batsch). Bataille	GenBank (KM596858); Jabeen et al. (2015b)
			*maculata*	Quél.	Sultana et al. (2011)
			*mansehraensis*	Saba, Caboň & Adamčík	GenBank (KU886598)
			*nitida*	(Pers.) Fr.	Razaq et al. (2019)
			*olivacea*	(Schaeff.) Fr.	Razaq et al. (2019)
			*paludosa*	Britzelm.	Razaq et al. (2019); Sultana et al. (2011)
			*pelargonia*	Niolle	Razaq et al. (2019); Sultana et al. (2011)
			*pectinatoides*	Peck	FOP
			*queletii*	Fr.	FOP
			*quercus-floribundae*	M. Kiran & Adamčík	GenBank (MN053391); Crous et al. (2019)
			*rosea*	Pers.	FOP; Sultana et al. (2011); Razaq et al. (2019)
			*rhodopodus*	Zvára	FOP
			*risigallina*	(Batsch) Sacc.	GenBank (KF679818)
			*romellii*	Maire	Razaq et al. (2019); Sultana et al. (2011)
			*rubricolor*	Jabeen, Naseer & Khalid	Jabeen et al. (2020b)
			*sanguinea*	Fr.	FOP
			*shanglaensis*	S. Ullah, Khalid & Fiaz	GenBank (MK579183); Ullah et al. (2020b)
			*sichuanensis*	G.J. Li & H.A. Wen	GenBank (KM596859); Saba and Khalid (2015)
			*swatica*	Sarwar and Hanif	Genbank (MK389374); Sarwar et al. (2019)
			*torulosa*	Bres.	Sultana et al. (2011)
Russulales	Russulaceae	*Russula*	*tuberculosa*	R. Heim	FOP
			*velenovskyi*	Melzer & Zvára	FOP
			*vinosa*	Lindblad	Sultana et al. (2011)
			*violacea*	Quél.	Sultana et al. (2011)
			*xerampelina*	(Schaeff.) Fr.	FOP
		*Thelephora*	*atlanticus*	Bon.	Sultana et al. (2011)
			*badiosanguineus*	Kühner & Romagn.	FOP
			*controversus*	Pers.	Sultana et al. (2011)
			*deliciosus*	(L.) Gray	FOP; Sultana et al. 2011
			*deterrimus*	Gröger	Sultana et al. (2011)
			*hatsudake*	Nobuj. Tanaka	FOP
			*helvus*	(Fr.) Fr.	Razaq and Shahzad (2012)
			*lacunarum*	Romagn. ex Hora	Sultana et al. (2011)
			*mediterraneensis*	Llistos. & Bellù	GenBank (MK607609)
			*obscuratus*	(Lasch) Fr.	Sultana et al. (2011); Razaq and Shahzad (2012)
			*pubescens*	Fr.	Razaq and Shahzad (2012)
			*quietus*	(Fr.) Fr.	Sultana et al. (2011)
			*romagnesii*	Bon	Sultana et al. (2011)
			*sanguifluus*	(Paulet) Fr.	GenBank (HE615155); FOP; Sultana et al. 2011; Ilyas et al. (2013b)
			*scrobiculatus*	(Scop.) Fr.	FOP; Sultana et al. (2011)
			*semisanguifluus*	R. Heim & Leclair	GenBank (HF559377); Sultana et al. (2011)
			*torminosus*	(Schaeff.) Pers.	FOP; Sultana et al. (2011)
			*vietus*	(Fr.) Fr.	Sultana et al. (2011)
			*violascens*	(J. Otto) Fr.	Sultana et al. (2011)
			*scrobiculatus*	(Scop.) Fr.	FOP
		*Lactifluus*	*brunneoviolascens*	(Bon) Verbeken	Sultana et al. (2011)
			*glaucescens*	(Crossl.) Verbeken	Razaq et al. (2014)
			*pergamenus*	(Sw.) Kuntze	Sultana et al. (2011)
			*piperatus*	(L.) Roussel	FOP; Sultana et al. (2011); Razaq and Shahzad (2012)
			*rugatus*	(Kühner & Romagn.) Verbeken	Sultana et al. (2011)
			*vellereus*	(Fr.) Kuntze	Sultana et al. (2011)
			*volemus*	(Fr.) Kuntze	Khan and Sher (2016c)
	Stereaceae	*Acanthofungus*	*ahmadii*	(Boidin) Sheng H. Wu, Boidin & C.Y. Chien	FOP
		*Aleurodiscus*	*jacksonii*	S. Ahmad	FOP
		*Amylostereum*	*chailletii*	(Pers.) Boidin	FOP
		*Gloeocystidiellum*	*porosum*	(Berk. & M.A. Curtis) Donk	FOP
		*Stereum*	*elegans*	(G. Mey.) Fr.	FOP
			*frustulosum*	(Pers.) Fr.	FOP
			*gausapatum*	(Fr.) Fr.	FOP
			*hirsutum*	(Willd.) Pers.	FOP
			*ostrea*	(Blume & T. Nees) Fr.	FOP
			*princeps*	(Jungh.) Lév.	FOP
			*rugosum*	Pers.	FOP
			*sanguinolentum*	(Alb. & Schwein.) Fr.	FOP
		*Xylobolus*	*subpileatus*	(Berk. & M.A. Curtis) Boidin	FOP
Incertae sedis	Incertae sedis	*Neoalbatrellus*	*caeruleoporus*	(Peck) Audet	Sultana et al. (2011)
Trechisporales	Hydnodontaceae	*Brevicellicium*	*olivascens*	(Bres.) K.H. Larss. & Hjortstam	FOP
	Bankeraceae	*Boletopsis*	*leucomelaena*	(Pers.) Fayod	FOP
		*Hydnellum*	*caeruleum*	(Hornem.) P. Karst.	FOP
Thelephorales	Bankeraceae	*Hydnellum*	*concrescens*	(Pers.) Banker	FOP
			*earlianum*	Banker	FOP
		*Sarcodon*	*imbricatus*	(L.) P. Karst.	FOP
	Thelephoraceae	*Phellodon*	*niger*	(Fr.) P. Karst.	FOP
		*Thelephora*	*anthocephala*	(Bull.) Fr.	FOP
			*arbuscula*	Corner	FOP
			*atra*	Weinm.	FOP
			*caryophyllea*	(Schaeff.) Pers.	FOP
			*fucoides*	Corner	FOP
			*iqbalii*	Nasir & Hanif	GenBank (JX241471); Khalid and Hanif (2017)
			*palmata*	(Scop.) Fr.	FOP
			*penicillata*	(Pers.) Fr.	FOP
			*terrestris*	Ehrh. ex Fr.	FOP
		*Tomentella*	*bryophila*	(Pers.) M.J. Larsen	FOP
			*coriaria*	(Peck) Bourdot & Galzin	FOP
			*griseo-cinnamomea*	Wakef.	FOP
			*punicea*	(Alb. & Schwein.) J. Schröt.	FOP
	Incertae sedis	*Dendrothele*	*acerina*	(Pers.) P.A. Lemke	FOP
Ascomycota/Geoglossales	Geoglossaceae	*Geoglossum*	*umbratile*	Sacc.	FOP
		*Trichoglossum*	*hirsutum*	(Pers.) Boud.	FOP
			*octopartitum*	Mains	FOP
			*velutipes*	(Peck) E.J. Durand	FOP
Helotiales	Calloriaceae	*Calloria*	*urticae*	(Pers.) J. Schröt. ex Rehm	GenBank (MN957392)
		*Diplonaevia*	*mollisioides*	(Sacc. & Briard) B. Hein	FOP
	Cenangiaceae	*Chlorencoelia*	*torta*	(Schwein.) J.R. Dixon	GenBank (MN957580)
		*Velutarina*	*rufo-olivacea*	(Alb. & Schwein.) Korf	FOP
	Helotiaceae	*Cyathicula*	*coronata*	(Bull.) Rehm	FOP
			*cyathoidea*	(Bull.) Thüm.	FOP
			*dolosella*	(P. Karst.) Dennis	FOP
			*egenula*	(Rehm) E. Müll.	FOP
		*Hymenoscyphus*	*calyculus*	(Fr.) W. Phillips	FOP
			*scutula*	(Pers.) W. Phillips	FOP
			*scutulavar.scutula*	(Pers.) W. Phillips	FOP
			*subferrugineus*	(Nyl.) Dennis	FOP
			*vitigenus*	(De Not.) Dennis	FOP
		*Tatraea*	*macrospora*	(Peck) Baral	FOP
	Lachnaceae	*Incrucipulum*	*ciliare*	(Schrad.) Baral	FOP
		*Lachnellula*	*arida*	(W. Phillips) Dennis	FOP
			*calyciformis*	(Batsch) Dharne	FOP
		*Lachnum*	*bicolor*	(Bull.) P. Karst	FOP
			*corticale*	(Pers.) Nannf.	FOP
			*indicus*	(E.K. Cash) J.H. Haines & Dumont	FOP
			*mollissiumum*	(Fuckel) P. Karst.	FOP
			*pudibundum*	(Quél.) J. Schröt.	FOP
		*Perrotia*	*himalayensis*	E. Müll. & Dennis	FOP
	Mollisiaceae	*Tapesia*	*fusca*	(Pers.) Fuckel	FOP
			*rosae*	(Pers.) Fuckel	FOP
	Pezizellaceae	*Allophylaria*	*subhyalina*	(Rehm) Baral	FOP
		*Calycina*	*citrina*	(Hedw.) Gray	FOP
			*chionea*	(Fr.) Kuntze	FOP
	Ploettnerulaceae	*Pyrenopeziza*	*lavaterae*	E. Müll. & S. Ahmad	FOP
	Rutstroemiaceae	*Rutrstroemia*	*bolaris*	(Batsch) Rehm	FOP
			*firma*	(Pers.) P. Karst.	FOP
	Sclerotiniaceae	*Moellerodiscus*	*berberidis*	Dumont	FOP
		*Lasiobelonium*	*fuscum*	(E. Müll. & Dennis) Raitv.	FOP
	Thelebolaceae	*Thelebolus*	*crustaceus*	(Fuckel) Kimbr.	FOP
	Incertae sedis	*Cistella*	*geelmuydenii*	Nannf.	FOP
Rhytismatales	Cudoniaceae	*Cudonia*	*circinans*	(Pers.) Fr.	FOP
	Hyaloscyphaceae	*Hyaloscypha*	*luteola*	S. Ahmad	FOP
Leotiales	Leotiaceae	*Leotia*	*lubrica*	(Scop.) Pers.	FOP
Orbiliales	Orbiliaceae	*Hyalorbilia*	*erythrostigma*	(W. Phillips) Baral & G. Marson	GenBank (MN957494)
		*Orbilia*	*auricolor*	(A. Bloxam) Sacc.	FOP
			*curvatispora*	Boud.	FOP
			*leucostigma*	(Fr.) Fr	FOP
Hypocreales	Bionectriaceae	*Hydropisphaera*	*erubescens*	(Roberge ex Desm.) Rossman & Samuels	GenBank (MN957491)
	Hypocreaceae	*Trichoderma*	*alutaceum*	Jaklitsch	FOP
Xylariales	Hypoxylaceae	*Daldinia*	*bakeri*	Lloyd	FOP
			*concentrica*	(Bolton) Ces. & De Not.	FOP
			*vernicosa*	Ces. & De Not.	FOP
	Xylariaceae	*Podosordaria*	*kurziana*	(Curr.) P.M.D. Martin	FOP
			*leporina*	(Ellis & Everh.) Dennis	FOP
			*nigripes*	(Klotzsch) P.M.D. Martin	FOP
			*pyramidata*	(Berk. & Broome) P.M.D. Martin	FOP
		*Poronia*	*indica*	S. Ahmad	FOP
			*polyporoides*	Henn.	FOP
		*Xylosphaera*	*ehrenbergii*	(Henn.) Dennis	FOP
		*Xylaria*	*hirtella*	Wakef.	FOP
			*hypoxylon*	(L.) Grev.	FOP
			*mali*	Fromme	FOP
			*mellissii*	(Berk.) Cooke	FOP
			*polymorpha*	(Pers.) Grev.	FOP
Pezizales	Ascobolaceae	*Ascobolus*	*americanus*	(Cooke & Ellis) Seaver	FOP
			*denudatus*	Fr.	FOP
			*elegans*	J. Klein	FOP
			*furfuraceus*	Pers.	FOP
			*immersus*	Pers.	FOP
			*leveillei*	Boud.	FOP
			*michaudii*	Boud.	FOP
			*minutus*	Boud.	FOP
			*perplexans*	Massee & E.S. Salmon	FOP
			*quezelii*	Faurel & Schotter	FOP
			*scatigenus*	(Berk. & M.A. Curtis) Brumm.	FOP
			*subglobosus*	Seaver	FOP
		*Saccobolus*	*citrinus*	Boud. & Torrend	FOP
			*depauperatus*	(Berk. & Broome) E.C. Hansen	FOP
			*glaber*	(Pers.) Lambotte	FOP
			*succineus*	Brumm.	FOP
			*truncatus*	Velen.	FOP
			*versicolor*	(P. Karst.) P. Karst.	FOP
	Ascodesmidaceae	*Ascodesmis*	*macrospora*	W. Obrist	FOP
			*microscopica*	(P. Crouan & H. Crouan) Le Gal	FOP
			*sphaerospora*	W. Obrist	FOP
			*nigricans*	Tiegh.	FOP
		*Lasiobolus*	*papillatus*	(Pers.) Sacc.	FOP
			*trichoboloides*	S.R. Khan & J.L. Bezerra	FOP
	Discinaceae	*Gyromitra*	*esculenta*	Pers. ex Fr.	FOP
			*infula*	(Schaeff.) Quél.	FOP
			*khanspurensis*	Jabeen & Khalid	GenBank (MF116159); Krisai-Greilhuber et al. (2017)
Pezizales	Discinaceae	*Discina*	*ancilis*	(Pers.) Sacc.	FOP
	Helvellaceae	*Helvella*	*acetabulum*	(L.) Quél.	FOP
			*albella*	Quél.	GenBank (MN814023)
			*atra*	J. König	GenBank (KF679807); FOP
			*bachu*	Q. Zhao, Zhu L. Yang & K.D. Hyde	GenBank (MN959917)
			*crispa*	(Scop.) Fr.	FOP
			*cupiliformis*	Razaq et al., (2014)	Sultana et al. (2011)
			*elastica*	Bull.	FOP
			*involuta*	Q. Zhao, Zhu L. Yang & K.D. Hyde	GenBank (MW447509)
			*lacunosa*	Afzel.	FOP
			*leucopus*	Pers.	Razaq et al. (2014)
			*monachella*	(Scop.) Fr.	Razaq et al. (2014)
			*pezizoides*	Afzel.	FOP
			*villosa*	Schaeff.	FOP
		*Paxina*	*queletii*	(Bres.) Stangl	FOP
	Morchellaceae	*Morchella*	*crassipes*	(Vent.) Pers.	GenBank (KP670934)
			*deliciosa*	Fr.	GenBank (MW558089)
			*esculenta*	(L.) Pers.	FOP; GenBank (MT957957)
			*elata*	Fr.	GenBank (MT977069)
			*pakistanica*	Jabeen & Khalid	GenBank (KX306760); Hernández-Restrepo et al. (2016)
			*pulchella*	Clowez & Franç. Petit	GenBank (MF400857); Badshah et al. (2018)
			*tridentina*	Bres.	GenBank (MT584841)
		*Verpa*	*bohemica*	(Krombh.) J. Schröt.	FOP
	Pezizaceae	*Ahmadea*	*dalanensis*	Aman & Khalid	GenBank (MT645090); Aman et al. (2020)
		*Iodophanus*	*carneus*	(Pers.) Korf	FOP
		*Ionopezia*	*gerardii*	(Cooke) Van Vooren	FOP
		*Mattirolomyces*	*spinosus*	(Harkn.) Kovács, Trappe & Alsheikh	GenBank (MT649183); FOP; Aman et al. (2020)
		*Pachyphlodes*	*conglomerata*	(Berk. & Broome) Doweld	GenBank (HG797006)
		*Paragalactinia*	*michelii*	(Boud.) Dennis	GenBank (JN836749); Ashraf et al. (2012)
			*succosa*	(Berk.) Van Vooren	GenBank (JN588568); Ashraf and Khalid (2012)
			*succosella*	(Le Gal & Romagn.) Van Vooren	GenBank (KM199729); Jabeen et al. (2015a)
		*Plicaria*	*trachycarpa*	(Curr.) Boud.	FOP
		*Peziza*	*badiofusca*	(Boud.) Dennis	FOP
			*cerea*	Bull.	FOP
			*micropus*	Pers.	FOP
			*pakistanica*	(S. Ahmad) S. Ahmad	FOP
			*repanda*	Pers.	FOP
			*vesiculosa*	Pers.	FOP
			*violacea*	Pers.	FOP
	Pyronemataceae	*Terfezia*	*arenaria*	(Moris) Trappe	FOP
		*Aleuria*	*aurantia*	(Pers.) Fuckel	FOP
			*boudieri*	(Höhn.) J. Moravec	FOP
		*Aleuria*	*murreana*	S. Ahmad	FOP
Pezizales	Pyronemataceae	*Byssonectria*	*fusispora*	(Berk.) Rogerson & Korf	FOP
		*Cheilymenia*	*granulata*	(Bull.) J. Moravec	FOP
			*pulcherrima*	(P. Crouan & H. Crouan) Boud.	FOP
			*theleboloides*	(Alb. & Schwein.) Boud.	FOP
		*Geopora*	*ahmadii*	Saba, T. Ashraf, Khalid & Pfister	GenBank (KY805996); Saba et al. (2019a)
			*arenicola*	(Lév.) Kers	FOP
			*arenosa*	(Fuckel) S. Ahmad	FOP
			*cooperi*	Harkn.	Ashraf and Khalid (2012)
			*cooperif.cooperi*		GenBank (JN558642)
			*foliacea*	(Schaeff.) S. Ahmad	FOP
			*pinyonensis*	Flores-Rent. & Gehring	GenBank (MK583663)
			*sumneriana*	(Cooke ex W. Phillips) M. Torre,	GenBank (MN860070)
		*Geopyxis*	*alpina*	Höhn.	Khalid et al. (2000)
			*majalis*	(Fr.) Sacc.	FOP
		*Humaria*	*hemisphaerica*	(F.H. Wigg.) Fuckel	FOP
		*Neottiella*	*hetieri*	Boud.	FOP
		*Octospora*	*humosa*	(Fr.) Dennis	FOP
			*plumbeoatra*	(E.K. Cash) D.C. Pant & V.P. Tewari	FOP
			*umbrina*	(E.K. Cash) S. Ahmad	FOP
		*Otidea*	*alutacea*	(Pers.) Massee	GenBank (MN495937)
			*leporina*	(Batsch) Fuckel	FOP
		*Pyronema*	*omphalodes*	(Bull.) Fuckel	FOP
			*domesticum*	(Sowerby) Sacc.	GenBank (MN957610)
		*Sepultariella*	*semiimmersa*	(P. Karst.) Van Vooren, U. Lindem. & Healy	FOP
		*Scutellinia*	*scutellata*	(L.) Lambotte	FOP
		*Trichophaea*	*gregaria*	(Rehm) Boud.	FOP
			*woolhopeia*	(Cooke & W. Phillips) Boud.	FOP
	Sarcoscyphaceae	*Kompsoscypha*	*waterstonii*	(Seaver) Pfister	FOP
	Sarcosomataceae	*Plectania*	*melastoma*	(Sowerby) Fuckel	FOP
		*Sarcoscypha*	*coccinea*	(Gray) Boud.	FOP
			*occidentalis*	(Schwein.) Sacc.	FOP
	Tuberaceae	*Tuber*	*puberulum*	Berk. & Broome	FOP
	Incertae sedis	*Coprotus*	*albidus*	(Boud.) Kimbr.	FOP
			*dextrinoideus*	Kimbr., Luck-Allen & Cain	FOP
			*granuliformis*	(P. Crouan & H. Crouan) Kimbr.	FOP
			*leucopocillum*	Kimbr., Luck-Allen & Cain	FOP
			*ochraceus*	(P. Crouan & H. Crouan) J. Moravec	FOP
			*niveus*	(Fuckel) Kimbr., Luck-Allen & Cain	FOP
			*sexdecimsporus*	(P. Crouan & H. Crouan) Kimbr. & Korf	FOP
Incertae sedis	Pulvinulaceae	*Pulvinula*	*orichalcea*	(Cooke) Rifai	FOP
	Tarzettaceae	*Tarzetta*	*bronca*	(Peck) Korf & J.K. Rogers	FOP
			*catinus*	(Holmsk.) Korf & J.K. Rogers	FOP
			*cupularis*	(L.) Lambotte	FOP

**Table 2.** Genus wise distribution in different ecoregions of Pakistan.

Genus	Total species	BXW	EAMCF	IRM	IVD	HSTPF	KWTP	NWTSF	NWHASM	ROK	RNPSD	SINSD	SRAM	TD	WHBF	WHSACF
Abortiporus	1	○	○	○	○	○	●	○	○	○	○	○	○	○	○	●
Acanthocystis	1	○	○	○	○	○	○	●	○	○	○	○	○	○	○	○
Acanthofungus	1	○	○	○	○	●	○	○	○	○	○	○	○	○	○	○
Agaricus	31	●	○	○	○	●	●	●	○	○	○	○	○	○	●	●
Agrocybe	6	●	○	○	○	○	○	●	○	○	○	○	○	○	●	○
Ahmadea	1	○	○	○	○	○	○	●	○	○	○	○	○	○	○	○
Albatrellus	1	○	○	○	○	○	○	○	○	○	○	○	○	○	○	●
Aleuria	3	○	○	○	○	●	○	●	○	○	○	○	○	○	○	○
Aleurodiscus	1	○	○	○	○	○	○	○	○	○	○	○	○	○	●	○
Allophylaria	1	○	○	○	○	○	○	○	○	○	○	○	○	○	●	○
Amanita	28	○	○	○	○	●	○	●	●	○	○	○	○	○	●	●
Amaropostia	1	○	○	○	○	○	○	○	○	○	○	○	○	○	○	●
Amphinema	1	○	○	○	○	●	○	○	○	○	○	○	○	○	○	○
Amylosporus	2	○	○	○	○	○	○	●	○	○	○	○	○	○	○	●
Amylostereum	1	○	○	○	○	●	○	○	○	○	○	○	○	○	○	○
Anomoloma	1	○	○	○	○	○	○	●	○	○	○	○	○	○	○	○
Anthracophyllum	1	○	○	○	○	●	○	○	○	○	○	○	○	○	○	○
Antrodia	1	○	○	○	○	○	○	●	○	○	○	○	○	○	○	○
Antrodiella	1	○	○	○	○	○	○	○	○	○	○	○	○	○	●	○
Aphelaria	1	○	○	○	○	●	○	○	○	○	○	○	○	○	○	○
Apioperdon	1	○	○	○	○	●	○	○	○	○	○	○	○	○	○	○
Aporium	1	○	○	○	○	○	○	●	○	○	○	○	○	○	○	○
Armillaria	2	○	○	○	○	○	○	○	○	○	○	○	○	○	●	○
Armillariella	2	○	○	○	○	○	○	○	○	○	○	○	○	○	●	○
Arrhenia	1	○	○	○	○	○	○	○	○	○	○	○	○	○	○	●
Ascobolus	12	○	○	○	○	○	●	●	○	○	○	○	○	○	●	●
Ascodesmis	4	○	○	○	○	○	○	●	○	○	○	○	○	○	○	○
Asterostroma	1	○	○	○	○	○	○	●	○	○	○	○	○	○	○	○
Astraeus	1	●	○	○	○	●	○	○	○	○	○	○	○	○	●	●
Athelia	1	○	○	○	○	○	○	○	○	○	○	○	○	○	○	○
Aureoboletus	1	○	○	○	○	○	●	○	○	○	○	○	○	○	○	○
Aurificaria	1	○	○	○	○	○	○	○	○	○	○	○	○	○	●	○
Auriscalpium	1	○	○	○	○	●	○	○	○	○	○	○	○	○	●	○
Baeospora	1	○	○	○	○	○	○	○	○	○	○	○	○	○	●	○
Battarrea	1	●	○	○	○	○	○	●	○	○	○	○	○	●	○	○
Bjerkandera	1	○	○	○	○	○	●	●	○	○	○	○	○	○	●	●
Bolbitius	1	○	○	○	○	○	○	●	○	○	○	○	○	○	○	○
Boletopsis	1	○	○	○	○	○	○	○	○	○	○	○	○	○	○	●
Boletus	8	○	○	○	○	●	●	○	○	○	○	○	○	○	●	●
Bondarzewia	1	○	○	○	○	○	○	○	○	○	○	○	○	○	●	○
Bovista	9	○	○	○	○	●	○	●	●	○	○	○	○	○	●	●
Bovistella	1	○	○	○	○	○	○	○	○	○	○	○	○	○	●	○
Brevicellicium	1	○	○	○	○	○	○	●	○	○	○	○	○	○	○	○
Britzelmayria	1	○	○	○	○	○	○	●	○	○	○	○	○	○	○	○
Brunneoporus	1	○	○	○	○	○	○	○	○	○	○	○	○	○	○	○
Bryoperdon	1	○	○	○	○	●	○	○	○	○	○	○	○	○	○	○
Butyriboletus	1	○	○	○	○	○	○	○	○	○	○	○	○	○	●	○
Byssomerulius	1	○	○	○	○	●	○	○	○	○	○	○	○	○	○	○
Byssonectria	1	○	○	○	○	○	○	○	○	○	○	○	○	○	●	○
Cabalodontia	1	○	○	○	○	○	○	●	○	○	○	○	○	○	○	○
Callistosporium	1	○	○	○	○	○	○	○	○	○	○	○	○	○	●	○
Calloria	1	○	○	○	○	○	○	○	○	○	○	○	○	○	○	○
Caloboletus	1	○	○	○	○	○	○	○	○	○	○	○	○	○	●	●
Calvatia	5	○	○	○	○	○	○	○	●	○	○	○	○	○	○	●
Calycina	2	○	○	○	○	●	○	○	○	○	○	○	○	○	●	○
Cantharellus	1	○	○	○	○	●	○	○	○	○	○	○	○	○	○	●
Cellulariella	1	○	○	○	○	○	○	○	○	○	○	○	○	○	○	○
Cerioporus	3	○	○	○	○	●	●	○	○	○	○	○	○	○	●	●
Ceriporia	3	○	○	○	○	○	○	●	○	○	○	○	○	○	○	○
Chaetocalathus	1	○	○	○	○	●	○	○	○	○	○	○	○	○	○	○
Chalciporus	1	○	○	○	○	○	●	○	○	○	○	○	○	○	●	○
Chamaemyces	1	○	○	○	○	●	○	○	○	○	○	○	○	○	○	○
Cheilymenia	3	○	○	○	○	●	○	●	○	○	○	○	○	○	●	○
Chlorencoelia	1	○	○	○	○	○	○	○	○	○	○	○	○	○	○	○
Chlorophyllum	4	○	○	○	○	○	●	○	○	○	○	○	○	○	●	○
Chondrostereum	1	○	○	○	○	●	○	○	○	○	○	○	○	○	○	○
Chroogomphus	5	○	○	○	○	○	○	○	●	○	○	○	○	○	●	○
Cistella	1	○	○	○	○	●	○	○	○	○	○	○	○	○	○	○
Clavaria	2	○	○	○	○	●	○	●	○	○	○	○	○	○	●	●
Clavariadelphus	5	○	○	○	○	●	○	○	○	○	○	○	○	○	●	○
Clavulina	4	○	○	○	○	●	○	○	○	○	○	○	○	○	●	●
Clavulinopsis	1	○	○	○	○	●	○	○	○	○	○	○	○	○	●	○
Climacodon	1	○	○	○	○	○	○	○	○	○	○	○	○	○	●	○
Clitocella	2	○	○	○	○	●	○	●	○	○	○	○	○	○	●	○
Clitocybe	7	○	○	○	○	●	○	○	○	○	○	○	○	○	●	○
Clitocybula	1	○	○	○	○	○	○	○	○	○	○	○	○	○	●	○
Clitopilus	5	○	○	○	○	○	○	●	○	○	○	○	○	○	○	○
Collybia	3	○	○	○	○	●	○	●	○	○	○	○	○	○	●	○
Collybiopsis	3	○	○	○	○	○	○	●	○	○	○	○	○	○	○	○
Coltricia	3	○	○	○	○	●	○	●	○	○	○	○	○	○	●	●
Colus	1	○	○	○	○	○	○	●	○	○	○	○	○	○	○	○
Coniophora	2	○	○	○	○	●	○	○	○	○	○	○	○	○	●	○
Conocybe	9	○	○	○	○	○	○	●	○	○	○	○	○	○	○	○
Coprinellus	8	●	○	○	○	○	●	●	○	○	○	○	○	○	○	●
Coprinopsis	6	○	○	○	○	○	●	●	○	○	○	○	○	○	●	○
Coprinus	2	○	○	○	○	○	○	●	○	○	○	○	○	○	●	○
Coprotus	7	●	○	○	○	●	○	●	○	○	○	○	○	○	●	●
Coriolopsis	1	○	○	○	○	●	○	○	○	○	○	○	○	○	○	○
Coriolus	1	○	○	○	○	●	○	●	○	○	○	○	○	○	●	●
Cortinarius	21	○	○	○	○	○	●	○	○	○	○	○	○	○	●	●
Craterellus	1	○	○	○	○	○	○	○	○	○	○	○	○	○	○	○
Crepidotus	4	○	○	○	○	●	○	○	○	○	○	○	○	○	○	○
Crinipellis	2	○	○	○	○	○	○	●	○	○	○	○	○	○	●	○
Crucibulum	1	○	○	○	○	●	○	○	○	○	○	○	○	○	●	○
Cudonia	1	○	○	○	○	○	○	○	○	○	○	○	○	○	○	○
Cyanoboletus	1	○	○	○	○	○	○	○	○	○	○	○	○	○	●	○
Cyathicula	4	○	○	○	○	●	○	○	○	○	○	○	○	○	●	●
Cyathus	3	○	○	○	○	○	○	●	○	○	○	○	○	○	○	○
Cyclocybe	1	○	○	○	○	○	○	○	○	○	○	○	○	○	●	○
Cystoderma	1	○	○	○	○	○	○	○	○	○	○	○	○	○	●	○
Cystodermella	2	○	○	○	○	○	●	○	○	○	○	○	○	○	○	○
Cystolepiota	1	○	○	○	○	○	○	●	○	○	○	○	○	○	○	○
Cystostiptoporus	1	○	○	○	○	○	○	○	○	○	○	○	○	○	○	●
Cytidia	1	○	○	○	○	○	○	○	○	○	○	○	○	○	●	○
Daedalea	3	○	○	○	○	●	○	●	○	○	○	○	○	○	○	○
Daedaleopsis	1	○	○	○	○	○	○	○	○	○	○	○	○	○	○	○
Daldinia	3	○	○	○	○	●	○	●	○	○	○	○	○	○	●	○
Deconica	4	○	○	○	○	○	○	●	○	○	○	○	○	○	●	○
Dendrocorticium	1	○	○	○	○	●	○	○	○	○	○	○	○	○	○	○
Dendrothele	1	○	○	○	○	●	○	○	○	○	○	○	○	○	○	○
Desarmillaria	1	○	○	○	○	○	○	○	○	○	○	○	○	○	●	○
Descolea	2	○	○	○	○	●	○	●	○	○	○	○	○	○	●	●
Dichostereum	2	○	○	○	○	●	○	●	○	○	○	○	○	○	○	○
Diplonaevia	1	○	○	○	○	○	○	○	○	○	○	○	○	○	●	○
Discina	1	○	○	○	○	○	○	○	○	○	○	○	○	○	●	○
Disciseda	1	○	○	○	○	○	○	●	○	○	○	○	○	○	○	●
Duportella	2	○	○	○	○	○	○	●	○	○	○	○	○	○	○	○
Earliella	1	○	○	○	○	○	○	○	○	○	○	○	○	○	○	○
Echinoderma	1	○	○	○	○	○	○	○	○	○	○	○	○	○	●	○
Entoloma	9	○	○	○	●	○	○	●	○	○	○	○	○	○	●	○
Epithele	1	○	○	○	○	○	○	●	○	○	○	○	○	○	○	○
Favolus	2	○	○	○	○	○	○	●	○	○	○	○	○	○	○	●
Fistulina	1	○	○	○	○	●	○	○	○	○	○	○	○	○	●	●
Flammulaster	2	○	○	○	○	○	○	●	○	○	○	○	○	○	○	●
Flammulina	3	○	○	○	○	○	○	○	○	○	○	○	○	○	●	○
Flavodon	1	○	○	○	○	○	○	○	○	○	○	○	○	○	○	○
Fomes	4	○	○	○	○	●	●	●	○	○	○	○	○	○	●	●
Fomitiporia	2	○	○	○	○	●	○	○	○	○	○	○	○	○	●	●
Fomitopsis	3	○	○	○	○	●	○	●	●	○	○	○	○	○	●	○
Funalia	3	○	○	○	○	○	○	●	●	○	○	○	○	○	○	●
Fuscoporia	4	○	○	○	○	●	○	○	○	○	○	○	○	○	●	●
Galerina	1	○	○	○	○	○	○	○	○	○	○	○	○	○	●	○
Ganoderma	8	○	○	○	○	●	●	●	○	○	○	○	○	○	●	●
Gastrosporium	1	○	○	○	○	○	○	●	○	○	○	○	○	○	○	○
Geastrum	16	○	○	○	○	●	●	●	○	○	○	○	○	○	●	●
Geoglossum	1	○	○	○	○	●	○	○	○	○	○	○	○	○	○	○
Geopora	8	○	○	○	○	●	●	●	○	○	○	○	○	○	○	●
Geopyxis	2	○	○	○	○	○	●	○	○	○	○	○	○	○	●	○
Gloeocystidiellum	1	○	○	○	○	●	○	○	○	○	○	○	○	○	○	○
Gloeophyllum	3	○	○	○	○	●	●	○	○	○	○	○	○	○	●	●
Gloeoporus	1	○	○	○	○	●	○	○	○	○	○	○	○	○	○	○
Gomphidius	1	○	○	○	○	○	○	○	○	○	○	○	○	○	●	○
Gomphus	2	○	○	○	○	●	○	○	○	○	○	○	○	○	●	○
Grammothele	1	○	○	○	○	○	○	●	○	○	○	○	○	○	○	○
Gymnopilus	12	○	○	○	○	●	○	●	○	○	○	○	○	●	●	●
Gymnopus	9	○	○	○	○	●	○	●	○	○	○	○	○	○	●	●
Gyrodon	1	○	○	○	○	○	●	○	○	○	○	○	○	○	○	○
Gyrodontium	1	○	○	○	○	○	○	●	○	○	○	○	○	○	○	○
Gyromitra	3	○	○	○	○	●	○	○	○	○	○	○	○	○	●	○
Hebeloma	7	○	○	○	○	○	○	●	●	○	○	○	○	○	●	○
Helvella	13	○	○	○	○	●	●	○	○	○	○	○	○	○	●	●
Henningsomyces	1	○	○	○	○	○	○	●	○	○	○	○	○	○	○	○
Hericium	4	○	○	○	○	●	○	○	○	○	○	○	○	○	●	●
Heterobasidion	4	○	○	○	○	●	○	○	●	○	○	○	○	○	●	●
Hexagonia	1	○	○	○	○	○	○	○	○	○	○	○	○	○	○	●
Hohenbuehelia	4	○	○	○	○	●	○	●	○	○	○	○	○	○	●	○
Homophron	1	●	○	○	○	○	○	○	○	○	○	○	○	○	●	○
Hortiboletus	2	○	○	○	○	●	○	○	○	○	○	○	○	○	●	●
Humaria	1	○	○	○	○	●	○	○	○	○	○	○	○	○	●	○
Hyalorbilia	1	○	○	○	○	○	○	○	○	○	○	○	○	○	○	○
Hyaloscypha	1	○	○	○	○	○	○	●	○	○	○	○	○	○	○	○
Hydnellum	3	○	○	○	○	●	○	●	○	○	○	○	○	○	○	●
Hydnophlebia	1	○	○	○	○	○	○	●	○	○	○	○	○	○	○	○
Hydnoporia	1	○	○	○	○	●	○	○	○	○	○	○	○	○	●	○
Hydnum	2	○	○	○	○	●	○	●	○	○	○	○	○	○	●	○
Hydropisphaera	1	○	○	○	○	○	○	○	○	○	○	○	○	○	○	○
Hygrocybe	7	○	○	○	○	●	○	○	○	○	○	○	○	○	●	○
Hygrophorus	6	○	○	○	○	○	●	○	○	○	○	○	○	○	●	●
Hymenagaricus	1	○	○	○	○	○	○	●	○	○	○	○	○	○	○	●
Hymenochaete	9	○	○	○	○	●	○	○	○	○	○	○	○	○	●	○
Hymenopellis	1	○	○	○	○	●	○	○	○	○	○	○	○	○	●	○
Hymenoscyphus	5	○	○	○	○	●	○	○	○	○	○	○	○	○	●	○
Hyphodermella	1	○	○	○	○	○	○	●	○	○	○	○	○	○	○	○
Hyphodontia	2	○	○	○	○	●	○	○	○	○	○	○	○	○	●	○
Hypholoma	4	○	○	○	○	●	○	○	○	○	○	○	○	○	●	●
Hypochnicium	1	○	○	○	○	●	○	○	○	○	○	○	○	○	○	○
Hypsizygus	1	○	○	○	○	○	○	○	○	○	○	○	○	○	●	○
Incrucipulum	1	○	○	○	○	●	○	○	○	○	○	○	○	○	○	○
Infundibulicybe	3	○	○	○	○	●	○	○	○	○	○	○	○	○	●	○
Inocutis	2	●	○	○	○	○	○	●	○	○	○	○	○	○	○	○
Inocybe	30	○	○	○	○	●	●	○	○	○	○	○	○	○	●	●
Inonotus	7	●	○	○	○	●	○	●	○	○	○	○	○	○	●	○
Inosperma	3	○	○	○	○	○	○	○	○	○	○	○	○	○	●	○
Iodophanus	1	○	○	○	○	○	○	●	○	○	○	○	○	○	○	○
Ionopezia	1	○	○	○	○	●	○	○	○	○	○	○	○	○	○	○
Irpex	2	○	○	○	○	○	○	●	○	○	○	○	○	○	○	○
Irpiciporus	1	○	○	○	○	○	○	●	○	○	○	○	○	○	○	○
Itajahya	1	○	○	○	○	○	○	●	○	○	○	○	○	○	○	○
Jahnoporus	1	○	○	○	○	○	○	○	○	○	○	○	○	○	○	○
Kompsoscypha	1	○	○	○	○	○	○	●	○	○	○	○	○	○	○	○
Kuehneromyces	1	○	○	○	○	○	○	○	○	○	○	○	○	○	○	●
Laccaria	6	○	○	○	○	●	○	○	●	○	○	○	○	○	●	○
Lachnellula	2	○	○	○	○	○	○	○	○	○	○	○	○	○	●	●
Lachnocladium	1	○	○	○	○	○	○	●	○	○	○	○	○	○	○	○
Lachnum	5	○	○	○	○	●	○	○	○	○	○	○	○	○	●	○
Lactarius	20	○	○	○	○	●	●	○	●	○	○	○	○	○	●	○
Lactifluus	7	○	○	○	○	○	●	○	○	○	○	○	○	○	●	●
Lactocollybia	2	○	○	○	○	○	○	●	○	○	○	○	○	○	○	○
Laetiporus	1	○	○	○	○	●	●	○	○	○	○	○	○	○	●	○
Langermannia	1	○	○	○	○	○	○	●	○	○	○	○	○	○	○	○
Lasiobelonium	1	○	○	○	○	○	○	○	○	○	○	○	○	○	●	○
Lasiobolus	2	○	○	○	○	○	○	●	○	○	○	○	○	○	●	○
Laxitextum	1	○	○	○	○	●	○	○	○	○	○	○	○	○	●	○
Leccinellum	2	○	○	○	○	○	●	○	○	○	○	○	○	○	○	○
Leccinum	4	○	○	○	○	○	●	○	○	○	○	○	○	○	●	○
Lentaria	3	○	○	○	○	○	○	○	○	○	○	○	○	○	●	○
Lentinellus	2	○	○	○	○	○	○	●	○	○	○	○	○	○	○	●
Lentinus	7	○	○	○	○	○	○	●	○	○	○	○	○	○	●	●
Lenzites	2	○	○	○	○	●	○	●	○	○	○	○	○	○	●	○
Leotia	1	○	○	○	○	●	○	○	○	○	○	○	○	○	●	○
Lepiota	23	○	○	○	○	○	○	●	○	○	○	○	○	●	●	○
Lepista	4	○	○	○	○	○	○	●	○	○	○	○	○	○	●	○
Leptonia	1	○	○	○	○	○	○	●	○	○	○	○	○	○	○	○
Leptoporus	1	○	○	○	○	●	○	○	○	○	○	○	○	○	●	○
Leucoagaricus	15	○	○	○	○	○	○	●○	○	○	○	○	○	○	●	●
Leucocoprinus	3	○	○	○	○	○	○	●	○	○	○	○	○	○	○	●
Leucocybe	1	○	○	○	○	○	○	○	○	○	○	○	○	○	●	○
Leucogyrophana	2	○	○	○	○	●	○	○	○	○	○	○	○	○	●	○
Leucopaxillus	2	○	○	○	○	○	○	○	○	○	○	○	○	○	●	○
Limacella	1	○	○	○	○	○	○	○	○	○	○	○	○	○	○	○
Limacellopsis	1	○	○	○	○	○	○	○	○	○	○	○	○	○	○	○
Lopharia	2	○	○	○	○	○	○	●	○	○	○	○	○	○	○	○
Lycoperdon	14	○	○	○	○	●	●	●	○	○	○	○	○	○	●	●
Lyophyllum	2	●	○	○	○	●	○	○	○	○	○	○	○	○	●	○
Lysurus	3	○	○	○	○	○	○	●	○	○	○	○	○	○	○	○
Macrocybe	1	○	○	○	○	○	○	●	○	○	○	○	○	○	○	○
Macrocystidia	1	○	○	○	○	○	○	○	○	○	○	○	○	○	○	○
Macrolepiota	4	○	○	○	○	○	○	○	○	○	○	○	○	○	●	●
Mallocybe	2	○	○	○	○	○	●	○	○	○	○	○	○	○	●	○
Marasmiellus	9	○	○	○	○	○	○	●	●	○	○	○	○	○	●	○
Marasmius	13	○	○	○	○	●	○	●	○	○	○	○	○	○	●	○
Mattirolomyces	1	○	○	○	○	○	○	●	○	○	○	○	○	○	○	○
Megacollybia	1	○	○	○	○	○	○	○	○	○	○	○	○	○	●	○
Melanogaster	1	○	○	○	○	○	○	○	●	○	○	○	○	○	○	○
Melanoleuca	5	○	○	○	○	●	○	●	●	○	○	○	○	○	○	●
Melanotus	1	○	○	○	○	○	○	●	○	○	○	○	○	○	○	○
Merismodes	1	○	○	○	○	○	○	○	○	○	○	○	○	○	●	○
Micropsalliota	3	○	○	○	○	○	○	●	○	○	○	○	○	○	○	○
Moellerodiscus	1	○	○	○	○	○	○	●	○	○	○	○	○	○	○	○
Montagnea	1	●	○	○	○	○	○	●	○	○	○	○	○	●	○	○
Morchella	7	○	○	○	○	●	○	○	○	○	○	○	○	○	●	●
Multiclavula	1	○	○	○	○	○	○	○	○	○	○	○	○	○	●	○
Mycena	7	○	○	○	○	●	●	○	○	○	○	○	○	○	●	○
Mycenastrum	1	●	○	○	○	○	○	●	○	○	○	○	○	○	○	○
Mycetinis	2	○	○	○	○	○	○	○	○	○	○	○	○	○	●	○
Mycorrhaphium	1	○	○	○	○	●	○	●	○	○	○	○	○	○	○	○
Myriostoma	1	○	○	○	○	○	○	○	○	○	○	○	○	○	●	●
Mythicomyces	1	○	○	○	○	○	○	○	○	○	○	○	○	○	○	○
Naucoria	3	○	○	○	○	○	○	●	●	○	○	○	○	○	○	○
Neoalbatrellus	1	○	○	○	○	○	○	○	○	○	○	○	○	○	○	○
Neoboletus	1	○	○	○	○	○	○	○	○	○	○	○	○	○	○	●
Neolentinus	1	○	○	○	○	○	○	○	○	○	○	○	○	○	●	●
Neottiella	1	○	○	○	○	○	○	○	○	○	○	○	○	○	●	○
Nothopanus	1	○	○	○	○	○	○	●	○	○	○	○	○	○	○	○
Octospora	3	○	○	○	○	○	○	●	○	○	○	○	○	○	○	○
Odontia	2	○	○	○	○	●	○	○	○	○	○	○	○	○	●	○
Omphalotus	1	○	○	○	○	○	●	○	○	○	○	○	○	○	●	○
Onnia	2	○	○	○	○	●	○	○	○	○	○	○	○	○	●	○
Orbilia	3	○	○	○	○	●	○	○	○	○	○	○	○	○	●	○
Otidea	2	○	○	○	○	●	○	○	○	○	○	○	○	○	●	○
Oxyporus	2	○	○	○	○	●	○	○	○	○	○	○	○	○	●	○
Pachyphlodes	1	○	○	○	○	○	○	○	○	○	○	○	○	○	○	○
Panaeolus	6	○	○	○	○	○	○	●	○	○	○	○	○	○	●	○
Panellus	1	○	○	○	○	●	○	○	○	○	○	○	○	○	○	○
Panus	1	○	○	○	○	○	○	○	○	○	○	○	○	○	●	●
Paragalactinia	3	○	○	○	○	○	○	○	○	○	○	○	○	○	●	○
Paralepista	1	○	○	○	○	○	●	○	○	○	○	○	○	○	●	○
Parasola	9	●	○	○	○	○	○	●	○	○	○	○	○	○	●	●
Paxina	1	○	○	○	○	○	○	○	○	○	○	○	○	○	●	○
Peniophora	2	○	○	○	○	●	○	○	○	○	○	○	○	○	●	●
Peniophorella	1	○	○	○	○	●	○	○	○	○	○	○	○	○	○	○
Perenniporia	1	○	○	○	○	●	○	○	○	○	○	○	○	○	○	○
Perrotia	1	○	○	○	○	○	○	○	○	○	○	○	○	○	●	○
Peziza	7	○	○	○	○	●	○	○	○	○	○	○	○	○	●	●
Phaeoclavulina	2	○	○	○	○	●	○	○	○	○	○	○	○	○	○	●
Phaeocollybia	1	○	○	○	○	●	○	○	○	○	○	○	○	○	○	○
Phaeodaedalea	1	○	○	○	○	●	○	○	○	○	○	○	○	○	○	○
Phaeolepiota	1	○	○	○	○	○	●	○	○	○	○	○	○	○	●	○
Phaeolus	2	○	○	○	○	●	●	○	○	○	○	○	○	○	○	●
Phaeophlebiopsis	1	○	○	○	○	○	○	○	○	○	○	○	○	○	●	○
Phallus	6	○	○	○	○	●	●	●	○	○	○	○	○	○	●	●
Phanerodontia	1	○	○	○	○	○	○	○	○	○	○	○	○	○	○	○
Phellinopsis	1	○	○	○	○	●	○	●	○	○	○	○	○	○	○	○
Phellinus	18	●	○	○	○	●	○	●	●	○	○	○	○	○	●	●
Phellodon	1	○	○	○	○	○	○	○	○	○	○	○	○	○	○	●
Phellorinia	1	○	○	○	○	○	○	●	○	○	○	○	○	○	○	○
Phlebia	3	○	○	○	○	●	○	●	○	○	○	○	○	○	●	○
Phlebiopsis	2	○	○	○	○	●	○	○	○	○	○	○	○	○	●	○
Phloeomana	1	○	○	○	○	○	○	○	○	○	○	○	○	○	●	○
Pholiota	8	○	○	○	○	●	○	●	○	○	○	○	○	○	●	●
Phylloporia	3	○	○	○	○	●	○	●	○	○	○	○	○	○	○	●
Phylloporus	3	○	○	○	○	○	○	●	○	○	○	○	○	○	●	●
Picipes	2	○	○	○	○	○	●	○	○	○	○	○	○	○	○	○
Pilatoporus	1	○	○	○	○	○	○	●	○	○	○	○	○	○	○	○
Pisolithus	2	○	○	○	○	○	○	●	○	○	○	○	○	○	○	○
Pistillaria	1	○	○	○	○	○	○	●	○	○	○	○	○	○	○	○
Plectania	1	○	○	○	○	○	○	○	○	○	○	○	○	○	●	○
Pleurotus	8	●	○	○	○	○	○	●	●	○	○	○	○	○	●	●
Plicaria	1	○	○	○	○	○	○	○	○	○	○	○	○	○	●	○
Plicatura	1	○	○	○	○	○	○	○	○	○	○	○	○	○	●	○
Pluteus	11	○	○	○	○	●	○	●	○	○	○	○	○	○	●	○
Podaxis	1	○	○	○	○	○	○	●	○	○	○	○	○	○	○	○
Podoscypha	5	○	○	○	○	○	○	●	○	○	○	○	○	○	○	●
Podosordaria	4	○	○	○	○	○	○	●	○	○	○	○	○	○	○	●
Polyporellus	1	○	○	○	○	○	○	○	○	○	○	○	○	○	●	○
Polyporus	3	○	○	○	○	○	●	●	○	○	○	○	○	○	●	●
Poria	2	○	○	○	○	○	○	●	○	○	○	○	○	○	○	○
Porodaedalea	2	○	○	○	○	○	○	○	○	○	○	○	○	○	○	○
Poronia	2	○	○	○	○	○	○	●	○	○	○	○	○	○	○	○
Porostereum	1	○	○	○	○	●	○	●	○	○	○	○	○	○	●	●
Porphyrellus	1	○	○	○	○	○	●	○	○	○	○	○	○	○	●	○
Postia	1	○	○	○	○	○	○	○	○	○	○	○	○	○	●	●
Protostropharia	1	○	○	○	○	○	○	○	○	○	○	○	○	○	●	○
Protubera	2	○	○	○	○	○	○	●	○	○	○	○	○	○	○	○
Psathyrella	13	○	○	○	○	○	○	●	○	○	○	○	○	○	●	●
Pseudoboletus	1	○	○	○	○	○	○	○	○	○	○	○	○	○	○	○
Pseudoinonotus	1	○	○	○	○	○	○	○	○	○	○	○	○	○	●	○
Pseudolaccaria	1	○	○	○	○	○	○	○	○	○	○	○	○	○	○	○
Pseudomerulius	1	○	○	○	○	○	○	●	○	○	○	○	○	○	●	○
Pseudosperma	7	○	○	○	○	○	○	○	○	○	○	○	○	○	●	●
Psilocybe	2	○	○	○	○	○	○	●	○	○	○	○	○	○	●	○
Pulvinula	1	○	○	○	○	○	○	○	○	○	○	○	○	○	●	○
Punjabia	1	○	○	○	○	○	○	●	○	○	○	○	○	○	○	○
Pycnoporellus	1	○	○	○	○	●	○	○	○	○	○	○	○	○	●	○
Pycnoporus	2	○	○	○	○	○	○	○	○	○	○	○	○	○	○	●
Pyrenopeziza	1	○	○	○	○	○	○	○	○	○	○	○	○	○	●	○
Pyrofomes	2	●	○	○	○	○	○	○	○	○	○	○	●	○	○	○
Pyronema	2	○	○	○	○	○	○	●	○	○	○	○	○	○	○	○
Ramaria	12	○	○	○	○	●	●	○	○	○	○	○	○	○	●	●
Ranadivia	1	○	○	○	○	○	○	○	○	○	○	○	○	○	●	○
Resinoporia	1	○	○	○	○	○	○	●	○	○	○	○	○	○	○	○
Resupinatus	2	○	○	○	○	○	○	●	○	○	○	○	○	○	●	○
Rhizochaete	1	○	○	○	○	●	○	○	○	○	○	○	○	○	●	○
Rhizopogon	1	○	○	○	○	○	○	○	●	○	○	○	○	○	●	●
Rhodocollybia	4	○	○	○	○	○	○	○	●	○	○	○	○	○	●	○
Rhodocybe	2	○	○	○	○	○	○	●	○	○	○	○	○	○	●	○
Rhodofomes	1	○	○	○	○	○	○	○	○	○	○	○	○	○	●	●
Rigidoporus	5	○	○	○	○	●	●	●	○	○	○	○	○	○	○	●
Rubroboletus	1	○	○	○	○	○	○	○	○	○	○	○	○	○	●	○
Russula	52	○	○	○	○	●	●	●	●	○	○	○	○	○	●	●
Rutrstroemia	2	○	○	○	○	○	○	○	○	○	○	○	○	○	●	○
Saccobolus	6	○	○	○	○	○	○	●	○	○	○	○	○	○	○	○
Sagaranella	1	○	○	○	○	○	○	○	○	○	○	○	○	○	●	○
Saproamanita	1	○	○	○	○	○	○	●	○	○	○	○	○	○	○	○
Sarcodon	1	○	○	○	○	○	○	○	○	○	○	○	○	○	○	●
Sarcodontia	1	○	○	○	○	○	○	○	○	○	○	○	○	○	●	○
Sarcoscypha	2	○	○	○	○	●	○	○	○	○	○	○	○	○	●	●
Schizophyllum	2	○	○	○	○	○	○	●	○	○	○	○	○	○	○	○
Schizopora	1	○	○	○	○	○	○	●	○	○	○	○	○	○	○	○
Schizostoma	2	○	○	○	○	○	○	●	○	○	○	○	○	○	○	○
Scleroderma	10	○	○	○	○	●	○	○	○	○	○	○	○	○	●	●
Scopuloides	2	○	○	○	○	○	○	●	○	○	○	○	○	○	○	○
Scutellinia	1	○	○	○	○	●	○	○	○	○	○	○	○	○	●	○
Scytinostroma	2	○	○	○	○	●	○	●	○	○	○	○	○	○	○	○
Secotium	1	○	○	○	○	○	○	○	○	○	○	○	○	○	●	●
Sepultariella	1	○	○	○	○	○	○	●	○	○	○	○	○	○	○	○
Serpula	1	○	○	○	○	○	○	○	○	○	○	○	○	○	●	●
Simocybe	1	○	○	○	○	○	●	○	○	○	○	○	○	○	○	○
Sparassis	3	○	○	○	○	○	○	○	○	○	○	○	○	○	●	●
Sphaerobolus	2	○	○	○	○	○	○	○	○	○	○	○	○	○	○	○
Steccherinum	1	○	○	○	○	●	○	○	○	○	○	○	○	○	○	○
Stereum	8	○	○	○	○	●	○	●	○	○	○	○	○	○	●	○
Stramatoscypha	1	○	○	○	○	○	○	●	○	○	○	○	○	○	○	○
Strobilomyces	2	○	○	○	○	○	○	○	○	○	○	○	○	○	●	●
Strobilurus	2	○	○	○	○	○	○	○	○	○	○	○	○	○	●	○
Stropharia	3	○	○	○	○	●	○	○	○	○	○	○	○	○	●	○
Suillellus	2	○	○	○	○	●	○	○	○	○	○	○	○	○	●	○
Suillus	14	○	○	○	○	●	●	○	●	○	○	○	○	○	●	○
Tapesia	2	○	○	○	○	○	○	○	○	○	○	○	○	○	●	●
Tapinella	2	○	○	○	○	●	○	●	○	○	○	○	○	○	●	○
Tarzetta	3	○	○	○	○	●	○	○	○	○	○	○	○	○	●	○
Tatraea	1	○	○	○	○	●	○	○	○	○	○	○	○	○	○	○
Tephrocybe	2	○	○	○	○	○	○	○	○	○	○	○	○	○	●	○
Terfezia	1	○	○	○	○	○	○	●	○	○	○	○	○	○	●	○
Termitomyces	10	●	○	○	○	○	○	●	○	○	○	○	○	○	●	●
Thelebolus	1	○	○	○	○	○	○	●	○	○	○	○	○	○	○	○
Thelephora	9	○	○	○	○	●	○	○	○	○	○	○	○	○	●	●
Tomentella	4	○	○	○	○	●	○	○	○	○	○	○	○	○	○	○
Tomophagus	1	○	○	○	○	○	○	●	○	○	○	○	○	○	○	○
Trametes	15	●	○	○	○	●	●	●	○	○	○	○	○	○	●	●
Trametopsis	1	○	○	○	○	●	○	○	○	○	○	○	○	○	○	○
Trichaptum	2	●	○	○	○	●	○	○	○	○	○	○	○	○	●	●
Trichoderma	1	○	○	○	○	●	○	○	○	○	○	○	○	○	○	○
Trichoglossum	3	○	○	○	○	●	○	●	○	○	○	○	○	○	●	○
Tricholoma	8	○	○	○	○	○	●	○	○	○	○	○	○	○	●	○
Tricholomopsis	5	○	○	○	○	○	●	○	●	○	○	○	○	○	●	○
Trichophaea	2	○	○	○	○	●	○	○	○	○	○	○	○	○	●	●
Trogia	1	○	○	○	○	○	○	●	○	○	○	○	○	○	○	○
Tropicoporus	1	○	○	○	○	●	○	●	○	○	○	○	○	○	●	○
Truncospora	2	○	○	○	○	○	○	●	○	○	○	○	○	○	○	○
Tubaria	2	○	○	○	○	○	○	●	○	○	○	○	○	○	○	●
Tuber	1	○	○	○	○	○	○	○	○	○	○	○	○	○	●	●
Tulostoma	25	●	○	○	○	●	●	●	○	○	○	○	○	○	○	●
Turbinellus	1	○	○	○	○	○	○	○	○	○	○	○	○	○	●	○
Tylopilus	3	○	○	○	○	○	●	○	○	○	○	○	○	○	●	●
Tyromyces	2	○	○	○	○	●	○	○	○	○	○	○	○	○	●	○
Velutarina	1	○	○	○	○	○	○	○	○	○	○	○	○	○	●	○
Verpa	1	○	○	○	○	●	○	○	○	○	○	○	○	○	○	○
Volvariella	6	○	○	○	○	○	○	●	○	○	○	○	○	○	○	●
Volvopluteus	2	●	○	○	○	○	○	●	○	○	○	○	○	○	●	○
Xanthagaricus	3	●	○	○	○	○	○	●	○	○	○	○	○	○	○	●
Xanthoconium	3	○	○	○	○	●	●	○	●	○	○	○	○	○	○	○
Xanthoporia	1	○	○	○	○	○	●	○	○	○	○	○	○	○	○	○
Xerocomus	4	○	○	○	○	○	●	●	○	○	○	○	○	○	●	○
Xeromphalina	1	○	○	○	○	●	○	○	○	○	○	○	○	○	○	○
Xerula	2	○	○	○	○	○	○	○	○	○	○	○	○	○	●	○
Xylaria	5	○	○	○	○	●	○	●	○	○	○	○	○	○	○	○
Xylobolus	1	○	○	○	○	●	○	○	○	○	○	○	○	○	●	●
Xylodon	1	○	○	○	○	○	○	○	○	○	○	○	○	○	●	○
Xylosphaera	1	○	○	○	●	○	○	○	○	○	○	○	○	○	○	○
Zhuliangomyces	2	○	○	○	○	○	○	●	○	○	○	○	○	○	○	○

Where, **BXW** = Baluchistan xeric woodlands, **EAMCF** = East Afghan montane conifer forests, **IRM** = Indus River Delta Arabian sea mangroves, **IVD** = Indus valley desert, **HSTPF** = Himalayan subtropical pine forests, **KWTP** = Karakorem west Tibetan plateau alpine steppe, **NWTSF** = North-western thorn scrub forest, **NWHASM** = North-western Himalayan alpine scrub & meadows, **RNPSD** = Registan north Pakistan sandy desert, **ROK** = Rann of Kutch seasonal marsh, **SINSD** = South Iran Nubo-Sindian desert & semi-desert, **SRAM** = Sulaiman range Alpine meadows, **WHBF** = Western Himalayan broadleaf forests, **WHSACF** = Western Himalayan subalpine conifer forests, **TD** = Thar Desert.

## ﻿Discussion

The compendium presented in Table 1 gives an overview of the macrofungal diversity of Pakistan known to date. It largely reposes on the checklist by Ahmad et al. (1997; 866 entries) published over two decades ago and its recent update (Khalid, *in press*), as well as taxa recently described with the use of molecular data, for example, *Russulafoetenoides* (Razaq et al. 2014), *Leucoagaricuslahorensis* (Qasim et al. 2015a), *Tulsotomaahmadii* (Hussain et al. 2015b), *Phaeocollybiapakistanica* (Khan et al. 2016a), *Descoleaquercina* (Khan et al. 2017a), *Amanitagriseofusca* (Kiran et al. 2018), *Leucoagaricusbrunneus* (Ullah et al. 2019), *Ahmadeadalanensis* (Aman et al. 2020) etc. Numerous edible mushrooms naturally occur in Pakistan including *Agaricusbisporus*, *Boletusedulis*, *Termitomycesumkowaan*, *Macrocybegigantea*, *Morchellaesculenta* (morels), *Ahmadeadalanensis* (truffles), *Pleurotuscystidiosus*, *Marasmiusoreades*, *Phelloriniaherculeana*, *Cantharelluscinereus* (chanterelle), *Coprinuscomatus* and more. Siddiqui et al. (2020) worked on the cultivation potential of two wild indigenous species of *Agaricus*, i.e. *A.bisporus* and *A.subrufescens* and obtained promising results for spawn production locally. In the future, more edible mushrooms can be worked on for their possible cultivation and commercialisation prospects.

We recorded 1,293 species belonging to 411 genera, 115 families and 24 orders. For comparison, Vaco-Palacios and Franco-Molano (2013) listed 1,239 macrofungal species from Colombia. Flores et al. (2012) reported 315 taxa, 163 genera and 20 orders from Guatemala. Kinge et al. (2020) recently presented an elaborate checklist of macrofungi in South Africa listing 1,008 species, 251 genera and 72 families. For comparison with a well-studied area, 3,173 species have been reported from Quebec (mycoquébec.org). Approximately 20,000 species of macrofungi are known worldwide (Hawksworth 2001). Unsurprisingly, since Agaricales is the largest order of macrofungi (Money 2016), it is by far the most commonly represented order with 47% species in the present taxonomic list followed by Polyporales (11%), Russulales (9%) and Pezizales (8%).

The highest number of taxa was recorded in the western Himalayan broadleaf forests ecoregion, which belongs to the temperate broadleaf and mixed forest biome and has been reported to be the richest in central China and eastern North America (Zhao et al. 1990; Martin et al. 1993). The second highest diversity was found in the north-western thorn scrub forests, which are categorised under deserts and xeric shrublands. This thorn scrub is considered as a degraded form of tropical dry forests (e.g. Champion and Seth 1968; Puri et al. 1989). This ecoregion includes semi-arid to arid climatic zones and a mean annual rainfall of less than 750 mm and a temperature range of 45 degrees or more in summers to temperatures dropping below freezing point in winters. Furthermore, ecoregions in tropical and subtropical coniferous forests and temperate coniferous forest biomes also show good macrofungal taxa representation. The ecoregion of western Himalayan subalpine conifer forests plays an important ecological role to vanguard the alpine meadows to the north. For instance, many Himalayan birds and mammals migrate seasonally between the steep mountain slopes, relying on adjacent habitats when the original ones are disturbed. Likewise, large-scale collection of morel mushrooms (*Morchella spp.*) from this ecoregion by local people for export overlaps with the breeding season of many pheasants and some mammals. Therefore, maintaining the biodiversity composition and ecological processes within this geologically young, highest mountain range on Earth requires particular conservation policies for this unique ecoregion (Wikramanayake et al. 2002). Finally, the ecoregions of flooded grasslands and savannahs, mangroves and montane grasslands and shrublands have either less than five percent or no representation. So, clearly there are missing data due to very limited exploration in several regions.

Although the data presented here will be useful to taxonomists, ecologists and conservation biologists, conclusive trends cannot be drawn as there are gaps in data due to extensive sampling in a few ecoregions, whereas other areas have been either neglected or unexplored. Therefore, the unexplored ecoregions of Pakistan need to be sampled extensively to give a full picture of the fungal diversity and endemism therein. Many countries and regions around the world have identified and listed endemic species, including the United States (Stein 2002), Russia (Griffin 1999), the Tuscan Region in Italy (Foggi et al. 2014) and New Caledonia (Wulff et al. 2013). The International Union for Conservation of Nature (IUCN) recently published a report on endemic threatened species on the Red List for each country (IUCN 2019). Redhead (1997) listed rare macrofungi of British Columbia, Canada, for each ecoregion. More recently, Enns et al. (2020) generated a list of endemic species, including a few fungal species as well, highlighting the status of target species for conservation.

In conclusion, this study provides a comprehensive list of macrofungi recorded in Pakistan as of the year 2020 and their known distribution by ecoregions. The otherwise scattered data have now been arranged and are available to be utilised by mycologists and other scientists as well as by amateur citizens. Most importantly, it can serve as a baseline information for further conservation studies and policy-making. Furthermore, these data also highlight the need for more sampling from less sampled areas like Sindh and Baluchistan Provinces. Our next step is to develop an online portal for fungi of Pakistan, where revisions of the current compendium can be done and new reports can be continuously added.

## Supplementary materials

Supplementary material 1 Macrofungi list by biome and ecoregion of Pakistan Data type Base data for biomes and ecoregions of Pakistan Explanation note WWF description data for biomes and ecoregion of Pakistan is compiled in one document for readers ease of understanding of ecoregions present in Pakistan. The source of data is WWF official site, wikipedia and Dopa explorer. However, author added macrofungal genera recorded from each ecoregion. https://binary.pensoft.net/file/681755This dataset is made available under the Open Database License (http://opendatacommons.org/licenses/odbl/1.0/). The Open Database License (ODbL) is a license agreement intended to allow users to freely share, modify, and use this Dataset while maintaining this same freedom for others, provided that the original source and author(s) are credited.Nourin Aman

Supplementary material 2 Detailed taxonomic checklist of macrofungi of Pakistan Data type Checklist with detailed references, localities, biome and ecoregion Explanation note The detailed references and localities of each taxon is given and allocated biome and ecoregion according to localities mentioned in references consulted. https://binary.pensoft.net/file/681756This dataset is made available under the Open Database License (http://opendatacommons.org/licenses/odbl/1.0/). The Open Database License (ODbL) is a license agreement intended to allow users to freely share, modify, and use this Dataset while maintaining this same freedom for others, provided that the original source and author(s) are credited.Nourin Aman, Abdul Nasir Khalid & Jean-Marc Moncalvo
