# Supplementary material for: ﻿A compendium of macrofungi of Pakistan by ecoregions
Source: MycoKeys. 2022 May 9;89:171–233. doi: 10.3897/mycokeys.89.81148 (PMC9849087; doi:10.3897/mycokeys.89.81148)
Supplement: Supplementary material 1 — Macrofungi list by biome and ecoregion of Pakistan [file mycokeys-89-171-s001.docx]

**Table: Summary of WWF Biomes and Ecoregions of Pakistan**

| Sr. no. | **Biome** | **Ecoregion** |
| --- | --- | --- |
| 1 | Tropical and subtropical coniferous forests (TSPF) | Himalayan subtropical pine forests (HSTPF) |
| 2 | Temperate broadleaf and mixed forests (TBLMF) | Western Himalayan broadleaf forests (WHBF) |
| 3 | Temperate coniferous forests (TCF) | Western Himalayan subalpine conifer forests (WHSACF) |
|  |  | East Afghan Montane conifer forests (EAMCF) |
| 4 | Deserts and xeric shrublands (DXS) | North western thorn scrub forest (NWTSF) |
|  |  | Indus valley desert (IVD) |
|  |  | Baluchistan xeric woodlands (BXW) |
|  |  | Thar desert (TD) |
|  |  | South Iran Nubo-Sindian desert and semi desert (SINSD) |
|  |  | Registan North-Pakistan sandy desert (RNPSD) |
| 5 | Flooded grasslands and savannas (FGS) | Indus River delta Arabian sea mangroves (IRM) |
|  |  | Rann of Kutch seasonal marsh (ROK) |
| 6 | Montane grasslands and shrublands (MGS) | Karakorem West Tibetan Plateau alpine steppe (KWTP) |
|  |  | Northwestern Himalayan alpine scrub & meadows (NWHASM) |
|  |  | Sulaiman Range Alpine meadows (SRAM) |

**Table 1:** Terrestrial ecoregions of Pakistan (Source: https://dopa-explorer.jrc.ec.europa.eu/country/PK)

**Detailed overview of WWF Biomes and Ecoregions of Pakistan**

1. **Biome: Tropical and subtropical coniferous forests**

They are characterized by the lesser rainfalls and modest flexibility in temperature change. These forests have various species of conifers, which utilizes needles to deal with the climate. These coniferous forests in Indochina are divided into dry and moist categories.


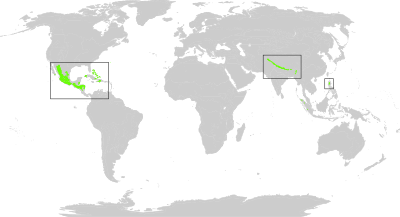


**Fig 1.** Worldwide distribution of Tropical and subtropical coniferous forests

(Source: https://en.wikipedia.org/wiki/Tropical_and_subtropical_coniferous_forests)

1. ***Himalayan subtropical pine forests (HSTPF)***

**Status:** Vulnerable

This ecoregion includes the portions of Nepal, Bhutan, India and Pakistan in the west. This large coniferous forest extends across the lower elevations covering area of 3000 km.


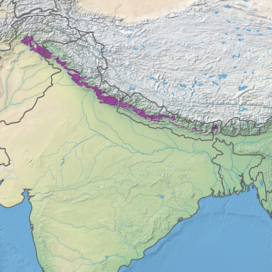


**Fig 2.** Distribution of Himalayan subtropical pine forests ecoregion

(Source: https://en.wikipedia.org/wiki/Himalayan_subtropical_pine_forests)

When the northwards drifting Deccan Plateau hit the northern Eurasia around 50 million years ago, Himalayan Mountain range was formed. The Himalayas currently comprises of three parallel zones: the outer Himalayas or Siwaliks on the southmost side neighbouring the Indo Gangetic Plain. The middle Himalayas, which consist of a series of ridges and valleys rising to roughly 5,000 metres and the Inner Himalayas, which include highest peaks like Mount Everest, K2, Makalu and Dhaulagiri.

The southwestern monsoon, which originates in the Bay of Bengal, is responsible for the majority of the rainfall. The eastern Himalayas which are closer to the Bay of Bengal receives most of the monsoon rains, resulting in less precipitation in the western Himalayas. The vegetation in the Himalayas is influenced by the climate gradient. The treeline in the western Himalayas, for example, is more than 50 metres lower than in the east (Kendrick 1989).

**Protected areas:**

Ayubia, Margalla hills, Mori Said Ali, Killan

**Dominant Plants**

The prominent flora in this ecoregion of subtropical pine forest is Chir pine. The understory is usually covered with thick grass, because the usual fires won’t let shrubs to grow and thrive. However, frequently burnt slopes support a rich growth of grasses including *Arundinella setosa*, *Imperata cylindrica, Themeda anathera*, and *Cymbopogon distans* and a number of shrubs such as species of *Berberis, Rubus*, and other thorny bushes (Shrestha and Joshi 1997).

**Macrofungi recorded**

*Acanthofungus, Agaricus, Aleuria, Amanita, Amphinema, Amylostereum, Anthracophyllum, Aphelaria, Apioperdon, Astraeus, Auriscalpium, Boletus, Bovista, Bryoperdon, Byssomerulius, Calycina, Cantharellus, Cerioporus, Chaetocalathus, Chamaemyces, Cheilymenia, Chondrostereum, Cistella, Clavaria, Clavariadelphus, Clavulina, Clavulinopsis, Clitocella, Clitocybe, Collybia, Coltricia, Coniophora, Coprotus, Coriolopsis, Coriolus, Crepidotus, Crucibulum, Cyathicula, Daedalea, Daldinia, Dendrocorticium, Dendrothele, Descolea, Dichostereum, Fistulina, Fomes, Fomitiporia, Fomitopsis, Fuscoporia, Ganoderma, Geastrum, Geoglossum, Geopora, Gloeocystidiellum, Gloeophyllum, Gloeoporus, Gomphus, Gymnopilus, Gymnopus, Gyromitra, Helvella, Hericium, Heterobasidion, Hohenbuehelia, Hortiboletus, Humaria, Hydnellum, Hydnoporia, Hydnum, Hygrocybe, Hymenochaete, Hymenopellis, Hymenoscyphus, Hyphodontia, Hypholoma, Hypochnicium, Incrucipulum, Infundibulicybe, Inocybe, Inonotus, Ionopezia, Laccaria, Lachnum, Lactarius, Laetiporus, Laxitextum, Lenzites, Leotia, Leptoporus, Leucogyrophana, Lycoperdon, Lyophyllum, Marasmius, Melanoleuca, Morchella, Mycena, Mycorrhaphium, Odontia, Onnia, Orbilia, Otidea, Oxyporus, Panellus, Peniophora, Peniophorella, Perenniporia, Peziza, Phaeoclavulina, Phaeocollybia, Phaeodaedalea, Phaeolus, Phallus, Phellinopsis, Phellinus, Phlebia, Phlebiopsis, Pholiota, Phylloporia, Pluteus, Porostereum, Pycnoporellus, Rhizochaete, Rigidoporus, Russula, Sarcoscypha, Scleroderma, Scutellinia, Scytinostroma, Steccherinum, Stereum, Stropharia, Suillellus, Suillus, Tapinella, Tarzetta, Tatraea, Thelephora, Tomentella, Trametes, Trametopsis, Trichaptum, Trichoderma, Trichoglossum, Trichophaea, Trichophaea, Tulostoma, Tyromyces, Verpa, Xanthoconium, Xeromphalina, Xylaria and Xylobolus.*

**Justification of Ecoregion Delineation by WWF (2001)**

The limits of the subtropical pine forests that traverse the length of the Himalayan Mountain range were delineated using MacKinnon’s (1997) computerised map of the distribution of the original vegetation.

1. **Biome: Temperate broadleaf and mixed forests**

The extent of these forests is in in central China and eastern North America in which these are richest and most distinct with other ecoregions in the Himalayas, The Caucasus, southern Europe, Southwestern South America and Russian far east.


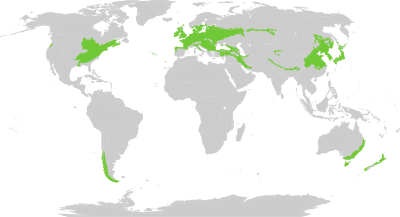


**Fig 3.** Worlwide distribution of Temperate broadleaf and mixed forests

(https://en.wikipedia.org/wiki/Temperate_broadleaf_and_mixed_forests)

1. ***Western Himalayan broadleaf forests (WHBF)***

**Status:** Critical/Endangered

The temperate broadleaf and mixed forest ecoregion found in the mid altitudes of the western Himalayas, covering parts of the Nepal, India and Pakistan, is known as Western Himalayan broadleaf forests.


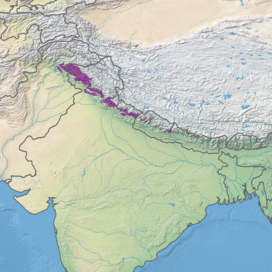


**Fig 4:** Distribution of Western Himalayan broadleaf forests ecoregion

(Source:https://en.wikipedia.org/wiki/Western_Himalayan_broadleaf_forests)

**Protected areas**

Manshi, Salkhala, Ghamot, Macharaya,

**Dominant Plants**

This ecoregion has two unique forest types: evergreen broad-leaved forests and deciduous broad-leaved forests. The former is frequently found on the wetter southern slopes, which are more influenced by the monsoon, and is dominated by *Quercus semecarpifolia*, *Quercus dilatata*, *Quercus lamellosa* and *Quercus incana* (Puri et al. 1989). Lauraceae species are frequently seen in these woodlands (e.g., *Machilus odoratissima*, *Litsea umbrosa*, *Litsea lanuginosa*, *Phoebe* *pulcherrima*). They also contain a rich understory, which includes mosses, ferns and various epiphytes on the trees, which are typical of damp forests at lower elevations. *Quercus ilex*, occasionally mixed with conifers such as *Abies*, *Picea*, *Cedrus* and Pinus species, dominates the drier forests, especially on the north-facing gradients and at higher altitudes with an understory dominated by *Arundinaria* (Puri et al. 1989).

**Macrofungi Recorded**

*Agaricus, Agrocybe, Aleurodiscus, Allophylaria, Amanita, Antrodiella, Armillaria, Armillariella, Ascobolus, Astraeus, Aurificaria, Auriscalpium, Baeospora, Bjerkandera, Boletus, Bondarzewia, Bovista, Bovistella, Butyriboletus, Byssonectria, Callistosporium, Caloboletus, Calycina, Cerioporus, Chalciporus, Cheilymenia, Chlorophyllum, Chroogomphus, Clavaria, Clavariadelphus, Clavulina, Clavulinopsis, Climacodon, Clitocella, Clitocybe, Clitocybula, Collybia, Coltricia, Coniophora, Coprinopsis, Coprinus, Coprotus, Coriolus, Cortinarius, Crinipellis, Crucibulum, Cyanoboletus, Cyathicula, Cyclocybe, Cystoderma, Cytidia, Daldinia, Deconica, Desarmillaria, Descolea, Diplonaevia, Discina, Echinoderma, Entoloma, Fistulina, Flammulina, Fomes, Fomitiporia, Fomitopsis, Fuscoporia, Galerina, Ganoderma, Geastrum, Geopyxis, Gloeophyllum, Gomphidius, Gomphus, Gymnopilus, Gymnopus, Gyromitra, Hebeloma, Helvella, Hericium, Heterobasidion, Hohenbuehelia, Homophron, Hortiboletus, Humaria, Hydnoporia, Hydnum, Hygrocybe, Hygrophorus, Hymenochaete, Hymenopellis, Hymenoscyphus, Hyphodontia, Hypholoma, Hypsizygus, Infundibulicybe, Inocybe, Inonotus, Inosperma, Laccaria, Lachnellula, Lachnum, Lactarius, Lactifluus, Laetiporus, Lasiobelonium, Lasiobolus, Laxitextum, Leccinum, Lentaria, Lentinus, Lenzites, Leotia, Lepiota, Lepista, Leptoporus, Leucoagaricus, Leucocybe, Leucogyrophana, Leucopaxillus, Lycoperdon, Lyophyllum, Macrolepiota, Mallocybe, Marasmiellus, Marasmius, Megacollybia, Merismodes, Morchella, Multiclavula, Mycena, Mycetinis, Myriostoma, Neolentinus, Neottiella, Odontia, Omphalotus, Onnia, Orbilia, Otidea, Oxyporus, Panaeolus, Panus, Paragalactinia, Paralepista, Parasola, Paxina, Peniophora, Perrotia, Peziza, Phaeolepiota, Phaeophlebiopsis, Phallus, Phellinus, Phlebia, Phlebiopsis, Phloeomana, Pholiota, Phylloporus, Plectania, Pleurotus, Plicaria, Plicatura, Pluteus, Polyporellus, Polyporus, Porostereum, Porphyrellus, Postia, Protostropharia, Psathyrella, Pseudoinonotus, Pseudomerulius, Pseudosperma, Psilocybe, Pulvinula, Pycnoporellus, Pyrenopeziza, Ramaria, Ranadivia, Resupinatus, Rhizochaete, Rhizopogon, Rhodocollybia, Rhodocybe, Rhodofomes, Rubroboletus, Russula, Rutrstroemia, Sagaranella, Sarcodontia, Sarcoscypha, Scleroderma, Scutellinia, Secotium, Serpula, Sparassis, Stereum, Strobilomyces, Strobilurus, Stropharia, Suillellus, Suillus, Tapesia, Tapinella, Tarzetta, Tephrocybe, Terfezia, Termitomyces, Thelephora, Trametes, Trichaptum, Trichoglossum, Tricholoma, Tricholomopsis, Trichophaea, Tropicoporus, Tuber, Turbinellus, Tylopilus, Tyromyces, Velutarina, Volvopluteus, Xerocomus, Xerula, Xylobolus, Xylodon.*

**Justification of Ecoregion Delineation by WWF (2001)**

MacKinnon's (1997) computerised map showing the distribution of original floristic composition was used to delimit the temperate forests from the broadleaf subtropical forests to the south and the sub-alpine conifer forests to the north.

1. **Biome: Temperate coniferous forests (TCF)**

This biome is mostly found in regions with cool winters and warm summers. The plant types differ in various areas. Pinus are dominant at certain places or other places have broadleaf evergreen trees or mixture of both of these tree types. Distinct type of these forests, tropical coniferous forests occur in tropical climates. They can also be found in coastal regions with mild winters and heavy rainfall or drier climate in inland or montane areas. trees common in these areas include cedar, fir, pine or redwood. Temperate forests are biomes with highest levels of biomass especially temperate rainforest regions. Structure wise, these forests mostly have 2 layers.


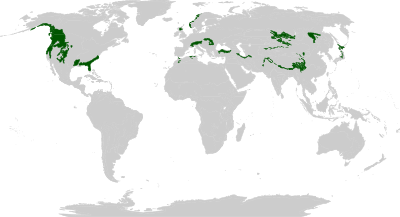


**Fig 5.** Worldwide distribution of temperate coniferous forests

(https://en.wikipedia.org/wiki/Temperate_coniferous_forest

1. ***Western Himalayan subalpine conifer forests (WHSACF)***

**Status:** Vulnerable

This ecoregion lies in Western Himalayan region against the treeless alpine meadows to the north.


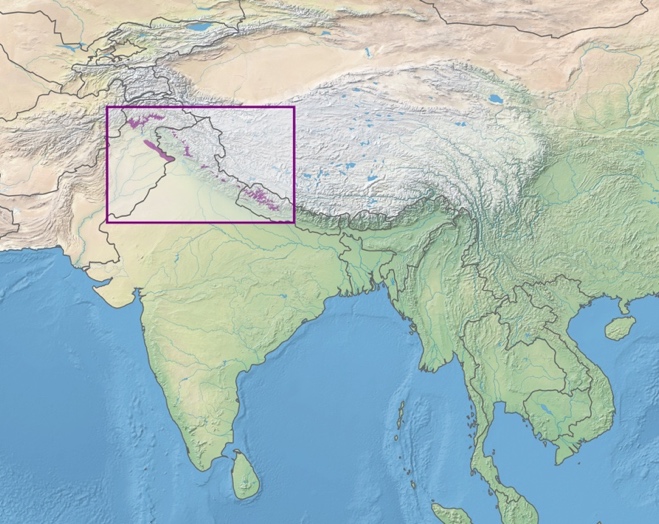


**Fig 6.** Distribution of Western Himalayan subalpine conifer forests ecoregion (Source: https://en.wikipedia.org/wiki/Western_Himalayan_subalpine_conifer_forests)

**Protected areas in Pakistan**

Himalaya National Park, Ayub, Tangir

**Dominant plants**

Based on floral affinities, this ecoregion features multiple distinct forest types including mixed oak-fir forest (*Quercus semecarpifolia* and *Abies spectabilis*), mixed coniferous forest (*Abies spectabilis*, *Pinus wallichiana*, and *Picea smithiana*) and mixed rhododendron, fir, and birch forest (*Rhododendron campanulatum*, *Abies spectabilis*, and *Betula utilis*) (Shrestha and Joshi 1997).

**Macrofungi recorded**

*Abortiporus, Agaricus, Albatrellus, Amanita, Amaropostia, Amylosporus, Arrhenia, Ascobolus, Astraeus, Bjerkandera, Boletopsis, Boletus, Bovista, Caloboletus, Calvatia, Cantharellus, Cerioporus, Clavaria, Clavulina, Coltricia, Coprinellus, Coprotus, Coriolus, Cortinarius, Cyathicula, Cystostiptoporus, Descolea, Disciseda, Favolus, Fistulina, Flammulaster, Fomes, Fomitiporia, Funalia, Fuscoporia, Ganoderma, Geastrum, Geopora, Gloeophyllum, Gymnopilus, Gymnopus, Helvella, Hericium, Heterobasidion, Hexagonia, Hortiboletus, Hydnellum, Hygrophorus, Hymenagaricus, Hypholoma, Inocybe, Kuehneromyces, Lachnellula, Lactifluus, Lentinellus, Lentinus, Leucoagaricus, Leucocoprinus, Lycoperdon, Macrolepiota, Melanoleuca, Morchella, Myriostoma, Neoboletus, Neolentinus, Panus, Parasola, Peniophora, Peziza, Phaeoclavulina, Phaeolus, Phallus, Phellinus, Phellodon, Pholiota, Phylloporia, Phylloporus, Pleurotus, Podoscypha, Podosordaria, Polyporus, Porostereum, Postia, Psathyrella, Pseudosperma, Pycnoporus, Ramaria, Rhizopogon, Rhodofomes, Rigidoporus, Russula, Sarcodon, Sarcoscypha, Scleroderma, Secotium, Serpula, Sparassis, Strobilomyces, Tapesia, Termitomyces, Thelephora, Trametes, Trichaptum, Trichophaea, Tubaria, Tuber, Tulostoma, Tylopilus, Volvariella, Xanthagaricus and Xylobolus.*

**Justification of Ecoregion Delineation by WWF (2001)**

Digital forest cover maps from MacKinnon (1997) were used to delineate the Himlaayan temperate conifer forests to the west of the kali Gandaki river, which are bounded to the north and south by alpine meadows and broadleaf forests, respectively. The western Himalayan sub-alpine conifer forests were then created from this belt of subalpine-coniferous forest.

1. ***East Afghan Montane conifer forests (EAMCF)***

**Status:** Vulnerable

The eastern Hindu Kush, Jalabad valley, Kunhar range and Ghazni province in the north make up the east Afghan montane coniferous forest. In the south, it reaches the Indus valley’s lower Kohistan, the Safed Koh, Takht-i-Suleiman and Koh-i-Maran ranges as well as western Pakistan’s Quetta Pass.


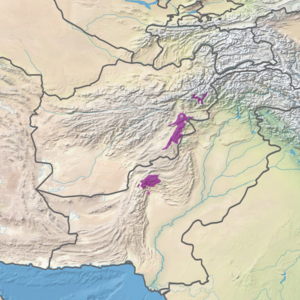


**Fig 7.** East Afghan Montane conifer forests ecoregion distribution

(Source: https://en.wikipedia.org/wiki/East_Afghan_montane_conifer_forests)

**Protected areas in Pakistan**

Hazarganji-Chiltan National Park, Sasnamana, Gogi, Zawarkhan

**Macrofungi recorded**

No records

**Dominant Plants**

Because of the monsoon’s effect, this ecoregion contains two types of forests. Between 2100 and 2500 metres, there is less monsoon rain and dry coniferous vegetation. Between the Fagaceae and Cedrus communities, a Pinaceae forest forms a belt. *Pinus gerardiana* and *Quercus baloot* are two examples. *Indigofera gerardiana*, *Sambucus ebulus* and *Plecanthrus rugosus* are among the understory plants found at this elevation. A temperate deciduous species combines with the conifers when one ascends to the higher elevations (2500-3100 m) where the monsoon rains continuously. At this elevation, Picea smithiana, Pinus wallichiana, Quercus semecarpifolia and Cedrus deodara form extensive forest cover with trees which are 15-30 m tall. The Cedrus deodara is one of Pakistan’s and Afghanistan’s most important timber species. The cedar forest is replaced by junipers at around 3100-3300 m, where the precipitation declines and the cedar forest is substituted by junipers. At higher elevations, hemicryptophytes including *Polygonum* *amplexicaule*, *Rumex nepalense*, *Fragaria nubicola* and *Berberis* species as well as geophytes like *Polygonatum geminiflorum*, *Lilium polyphyllum*, and *Habenaria aitchisonii*, dominate the herbaceous ground vegetation (Hassinger 1968; Freitag 1971).

1. **Biome: Deserts and xeric shrublands:**

There is great variation in temperature and rainfall amount globally. But overall evaporation rate is more than that of precipitation usually less than 10 inches annually. Temperature is also variable in various parts of world such as Gobi Desert of Asia while the Sahara and many other deserts are hot throughout the year.

1. ***North western thorn scrub forest (NWTSF)***

**Status:** Critical/Endangered

The ecoregion represents the thorn scrub forests of the Indian subcontinent’s northern region. This thorn scrub is considered by many ecologists to represent a deteriorated stage of tropical dry forests (e.g., Champion and Seth 1968; Puri et al. 1989). The ecoregion spans the India-Pakistan border and includes sections of India’s Gujarat, Rajasthan, Haryana and Punjab provinces as well as the lower regions of Jammu and Kahmir. In this ecoregion, the average annual rainfall is less than 750 mm. During the summer, temperature can reach 45 ° C, while in the winter, temperatures can dip to below freezing.


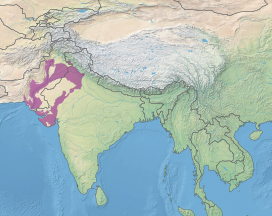


**Fig 8.** North western thorn scrub forest ecoregion distribution

(Source:https://en.wikipedia.org/wiki/Northwestern_thorn_scrub_forests)

**Protected areas**

Dhrun, Kirthar national park, Mahal Kohistan, Hab dam, Keenjhar lake, Hadero lake, Haleji lake, Bijoro Chach, Deh Jangisar, Goleen Gol, Khipro, Indus river, Dhong Block, Dosu Forest, Drigh Lake, Mando Dero, Sheikh Badin, Kalabagh game reserve, Thandarwala, Namal lake, Chasma lake, Taunsa Barrage, Kot Zabzai, Bhon Fazil, Gatwala, Kharar lake, Kamalia Plantation, Head Islam/Chak Kotora, Daulana, Daphar, Head Qadirabad, Chichawatini Plantation, Bahawalpur Plantation

**Dominant Plants**

The vegetation is stunted and open, dominated by *Acacia* species such as *A. senegal* and *A. leucophloea* that rarely exceed 6 m in height. Other characteristic species that make up the vegetation are *Prosopis spicigera*, *Capparis zeylanica*, *Salvadora* spp., *Carissa* spp., *Gymnosporia* spp., *Grewia* spp., and *Gardenia* spp. and xerophytic climbers such as species of *Tragia*, *Rivea*, *Tinospora*, *Vitis*, and *Peristrophe* (Champion and Seth 1968; Puri et al. 1989). In drier areas, the thorn forest transitions into xerophytic shrubland and semiarid vegetation, usually dominated by *Euphorbia* species. Intermingled with the *Euphorbia* scrub is a *Zizyphus* scrub (Champion and Seth 1968) that is characterized by *Zizyphus nummularia* with *Acacia leucocephala*, *Acacia senegal*, *Anogeissus pendula*, and *Dicrostachys cinerea*. The poor soils along rocky tracts promote a *Cassia-Butea* community. Closer to the coast, where the soils are more saline, the community includes *Salvadora and Tamarix* (Puri et al. 1989).

Acacia species such as *A*. *senegal* and *A*. *leucopholea*, which rarely reach 6 m in height, dominate the vegetation, which overall is stunted and open. *Prosopis spicigera*, *Capparis* *zeylanica*, *Salvadora* spp., *Carissa* spp., *Gymnosporia* spp., *Grewia* spp., *Gardenia* spp. and xerophytic climbers such as species of *Tragia*, *Rivea*, *Tinospora*, *Vitis*, and *Peristrophe* (Champion and Seth 1968; Puri et al. 1989). The thorn forest changes to xerophytic shrubland and semiarid vegetation in drier places, which is mainly dominated by *Euphorbia* species. A *Zizyphus* scrub (Champion and Seth 1968) coexists with the *Euphorbia* scrub, containing *Zizyphus nummularia*, *Acacia leucocephala*, *Acacia Senegal*, *Anogeissus pendula* and *Dicrostachys cinerea*. The *Cassia-Butea* community thrives on low soils along rocky stretches. *Salvadora* and *Tamarix* are located closer to the coast, where the soils are more saline (Puri et al. 1989).

**Macrofungi recorded**

*Acanthocystis, Agaricus, Agrocybe, Ahmadea, Aleuria, Amanita, Amylosporus, Anomoloma, Antrodia, Aporium, Ascobolus, Asterostroma, Battarrea, Bjerkandera, Bolbitius, Bovista, Brevicellicium, Britzelmayria, Cabalodontia, Ceriporia, Cheilymenia, Clavaria, Clitocella, Clitopilus, Collybia, Collybiopsis, Coltricia, Colus, Conocybe, Coprinellus, Coprinopsis, Coprinus, Coprotus, Coriolus, Crinipellis, Cyathus, Cystolepiota, Daedalea, Daldinia, Deconica, Descolea, Dichostereum, Disciseda, Duportella, Entoloma, Epithele, Favolus, Flammulaster, Fomes, Fomitopsis, Funalia, Ganoderma, Gastrosporium, Geastrum, Geopora, Grammothele, Gymnopilus, Gymnopus, Gyrodontium, Hebeloma, Henningsomyces, Hohenbuehelia, Hyaloscypha, Hydnellum, Hydnophlebia, Hydnum, Hymenagaricus, Hyphodermella, Inocutis, Inonotus, Iodophanus, Irpex, Irpiciporus, Itajahya, Kompsoscypha, Lachnocladium, Lactocollybia, Langermannia, Lasiobolus, Lentinellus, Lentinus, Lenzites, Lepiota, Lepista, Leptonia, Leucoagaricus, Leucocoprinus, Lopharia, Lycoperdon, Lysurus, Macrocybe, Marasmiellus, Marasmius, Mattirolomyces, Melanoleuca, Melanotus, Micropsalliota, Moellerodiscus, Montagnea, Mycenastrum, Mycorrhaphium, Naucoria, Nothopanus, Octospora, Panaeolus, Parasola, Phallus, Phellinopsis, Phellorinia, Phlebia, Pholiota, Phylloporia, Phylloporus, Pilatoporus, Pisolithus, Pistillaria, Pleurotus, Pluteus, Podaxis, Podoscypha, Podosordaria, Polyporus, Poria, Poronia, Porostereum, Protubera, Psathyrella, Pseudomerulius, Psilocybe, Punjabia, Pyronema, Resinoporia, Resupinatus, Rhodocybe, Russula, Saproamanita, Schizophyllum, Schizopora, Schizostoma, Scopuloides, Scytinostroma, Sepultariella, Stereum, Stramatoscypha, Tapinella, Terfezia, Termitomyces, Thelebolus, Tomophagus, Trametes, Trichoglossum, Trogia, Tropicoporus, Truncospora, Tubaria, Tulostoma, Volvariella, Volvopluteus, Xanthagaricus, Xerocomus, Xylaria and Zhuliangomyces.*

**Justification of Ecoregion Delineation by WWF (2001)**

To demarcate and differentiate the thorn scrub forests from the desert and dry deciduous forests, we used MacKinnon’s (1997) computerised map of the original vegetation. As a result, the thorn scrub that surrounds the thar desert was assigned to the Northwestern thorn scrub forests.

1. ***Indus valley desert***

**Status:** Vulnerable

The Indus valley in Pakistan is home to this dry ecoregion. Its western and eastern boundries are defined by the Glaiman Range’s foothills and the Chenab river, respectively. The dramatic annual temperature changes can vary from near-freezing in the winter to temperatures exceeding 45 °C in the summer. Grewal (1992) estimates that annual rainfall ranges from 640 to 760 mm, somewhat higher than the Thar desert.

**Protected area**

Thal

**Dominant plants**

The harsh climate regime has a significant impact on the plants. Isolated clumps of *Prosopis* spp., *Salvadora oleoides* and *Caparis* spp. as well as higher thorn-scrub forests of *Acacia* spp., *Tamarix* spp., *Albizzia lebbek* and *Morus alba*, characterise the desert thorn scrub vegetation (Grewal 1992).

**Macrofungi recorded**

No records

**Justification of Ecoregion Delineation by WWF (2001)**

The deserts of northeastern India and Pakistan were divided into four subunits by MacKinnon (1997). Based on the presence of distinctive habitat at regional geographic scales, WWF regrouped these subunits into eight ecoregions. The desert habitat in one of subunit was named as Indus valley desert using MacKinnon’s biounit framework and his digital map of original habitat.

1. ***Baluchistan Xeric woodlands (BXW)***

**Status:** Critical/Endangered


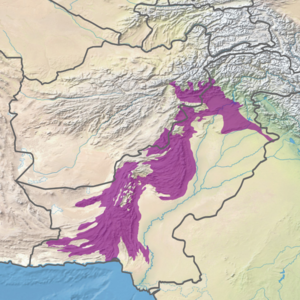


**Fig 9.** Baluchistan Xeric woodlands ecoregion distribution

(Source: https://en.wikipedia.org/wiki/Baluchistan_xeric_woodlands)

The Baluchistan xeric woodlands ecoregion stretches from the Las Bela valley to the high plateau of Baluchistan. It stretches to the north, passing through the Khyber Pakhtunkhwa’s trans-Indus Plains, Peshawar, Kohat and Bannu and extending till the Himalayan mountains’ eastern Hindu kush boundary. This ecoregion is surrounded by and has a tangle of ranges, hills and mountains. The Sulaiman, Kirthar, Safed Koh, and Pub Mountain ranges, the Torghar and Kaliphat Waziristan hills and the Tobakakar, Takhatu and Zarghun mountains, all of which have altitutes ranging from 1000 to 3000 metres, are among them. These ranges are pierced through by large passes like Quetta and Khyber.

**Protected Areas**

Raghai Rakhshan, Chorani, Shashan, Bund Khush Dil Khan, Maslakh, Ziarat Juniper, Dhrun, Surjan, Sumbak, Eri, and Hothiano, Dureji, Bilyamin, Manglot, Nizampur, Shina-Wari Chapri, Kala Chitta, Borraka, Khari Murat, Islamabad, Chinji, Diljabba-Domeli, Chumbi Surla, Rasool Barrage, Bajwat

**Dominant Plants**

Tropical steppe flora (below 1500 m) and open xeric forests (1500 to 2000 m) are found in the area (Hassinger 1968). Pistachio (*Pistachia atlantica, P. khinjuk*), almond (*Prunus rosaceae, P. eburnea)*, barberry (*Berberis*), honeysuckle bush (*Lonicera caprifoliaceae*, *L. hypoleuca*), *lycium* (Solanaceae), sage or wormwood (*Artemesia* spp.), and juniper (Juniperus macropoda, *J. semiglobosa*, and *J. seravschanica*) encompasses sections of the Baluchistan and Kurram valleys. Perennial grasses, tropical shrubs and acacia make up the ground layer that dominates the forests south of the ecoregion. *Amygdalus communis, Amygdalus kuramica* and *Fraxinus xanthoxyloides* are among them. Because of the excessive logging and overgrazing, the Indus Plain east of the Indus River and north of the ecoregion has been severly damaged. It maintains the communities of Tamaricaceae, Gramineae, Leguminosae and Rhamnaceae. *Tamarix* spp., *Saccharum spontaneum*, *Acacia arabica*, *Salvadora oleoides*, *S. persica* and *Zizyphus mauritiana* are among them. On these slopes, small thorny trees and acacia can be found, e.g., *Olea ferruginea, Acacia modesta, and Artemisia maritima*. Perennial grasses like *Poa* and *Bromus* spp., and bulbous plants like *Iris*, *Tulipa* and *Allium* spp. are also found in this ecoregion.

**Macrofungi recorded**

*Agaricus, Agrocybe, Astraeus, Battarrea, Coprinellus, Coprotus, Homophron, Inocutis, Inonotus, Lyophyllum, Montagnea, Mycenastrum, Parasola, Phellinus, Pleurotus, Pyrofomes, Termitomyces, Trametes, Trichaptum, Tulostoma, Volvopluteus and Xanthagaricus.*

**Justification of Ecoregion Delineation by WWF (2001)**

From MacKinnon's Baluchistan component, five ecoregions were found: Suleiman Range Alpine Meadows, South Iran Nubo-Sindian Desert and Semi-Desert, Baluchistan Xeric Woodlands, Rajasthan-North Pakistan Sandy Desert, and East Afghan Montane Coniferous Forests. To the north, there are the Hindu Kush mountains, and to the south and west, there are the Anatolian-Iranian Desert, Iranian Desert, and Caucaso-Iranian highlands.

***d. Thar desert (TD)***

**Status: Vulnerable**

Thar Desert is the seventh largest desert in the world and no doubt, the Indo-Pacific region's most inhabitable ecoregion. The Mohenjo Daro and Harappa civilizations, which are thought to be among the world's earliest civilizations, flourished in this region between 4,000 and 5,000 years ago (Chaudhry et al. 1997). The deserts that cover parts of the Indian states of Gujarat, Rajasthan, and Punjab, as well as Pakistan's Punjab and Sind, are part of this huge ecoregion to the west of the Aravalli Mountain Range in northern India.


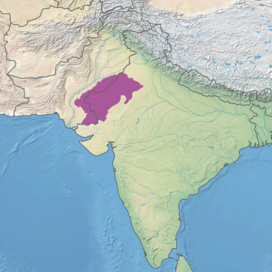


**Fig 10.** Thar desert ecoregion distribution

(Source: https://en.wikipedia.org/wiki/Thar_Desert)

**Dominant plants**

The habitat is greatly influenced by the extreme climate. The sparse vegetation consists of xerophilious grasslands of *Eragrostis* spp. *Aristida adscensionis*, *Cenchrus biflorus*, *Cympogon* spp., *Cyperus* spp., *Eleusine* spp., *Panicum* spp., *Lasiurus scindicus*, *Aeluropus lagopoides*, and *Sporobolus* spp. (Mares 1999). Scrub vegetation consists of low trees such as *Acacia nilotica*, *Prosopis cineraria*, *P. juliflora*, *Tamrix aphylla*, *Zizyphus mauritiana*, *Capparis decidua*, and shrubs such as *Calligonum polygonoides*, *Calotropis* spp., *Aerva* spp., *Crotalaria* spp., and *Haloxylon salicornicum*. *Haloxylon recurvum* is also present (Puri et al. 1989; Mares 1999).

**Macrofungi recorded:**

*Battarrea, Gymnopilus, Lepiota, Montagnea.*

**Justification of Ecoregion Delineation by WWF (2001)**

MacKinnon (1997) divided the deserts of northwestern India and Pakistan into four components in a prior study of conservation units. Based on the extent of distinctive habitat at regional spatial scales, these biounits were reclassified into eight ecoregions using MacKinnon's (1997) digital map of original habitat. The Thar deserts were given to their own ecoregion, the Thar Desert using this schema.

***e. South Iran Nubo-Sindian desert and semi desert (SINSD)***

**Status:** Critical/Endangered

It includes the Persian Gulf's northern coastal plain and the inland desert hills south of Iran's Zagros Mountains. Extensions of the region can also be found in southern Iraq and southwestern Pakistan. Inland, there are scorching sand deserts, shrubland, and open thorn forests. Along the coast, mangrove forests and marshes can be found.

The ecoregion runs 1,600 kilometres (990 miles) along the northern coast of the Persian Gulf, from the Gulf's head at Abadan to the Hingol River's estuary on Pakistan's southern coast, 150 kilometres west of Karachi. The region reaches 120 kilometres inland for the majority of its length, but it widens significantly east of the Strait of Hormuz. The ecoregion is located on the Makran Coastal Range of mountains as it enters Pakistan. With a mean elevation of 559 metres (1,834 ft), elevations range from sea level to 3,283 metres (10,771 feet).


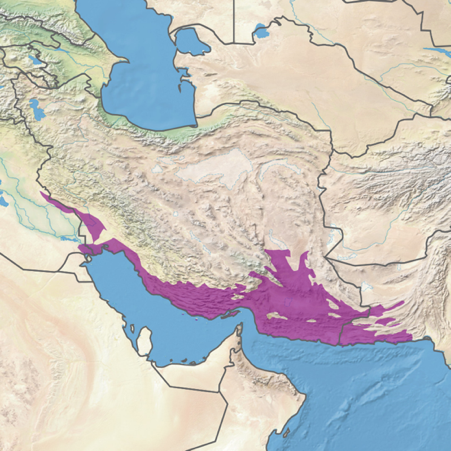


**Fig 11.** South Iran Nubo-Sindian desert and semi desert ecoregion distribution

(Source: https://en.wikipedia.org/wiki/South_Iran_Nubo–Sindian_desert_and_semi-desert)

**Dominant Plants**

Shadegan Ponds, on the Karun River's delta, are located at the Gulf's northernmost point. Bulrush (*Scirpus*) dominates brackish sedge marshes in the better-drained parts, and *Tamarix* dominates the mudflats in this internationally significant wetland. A representative mangrove population can be found at the Rud-e-Gaz and Rud-e-Hara River deltas in Hormuz, further south along the coast. There are tidal creeks, mudflats, and a mangrove swamp in this area. Large stands of Grey mangrove (*Avicennia marina*), often known as the Hara tree, can be found at the mouths of the rivers.

**Macrofungi recorded**

No records

**Justification of Ecoregion Delineation by WWF (2001)**

The hills north of the Persian Gulf and south of Iran's mountain ranges are home to this ecoregion. The geobotanical map of the Middle East by Zohary (1973) was used to set the boundaries. In the Sudanian and Sub-Sudanian vegetation zones, he classifies this region as Acacietea flavae iranica and Nubo-Sindian vegetation. The boundary in Pakistan corresponds to the thorn scrub forest in Mackinnon's (1997) original vegetation map of the Indo-Malayan biogeographic realm, which is part of the Thar-Indus bioregion.

***f. The Registan–North Pakistan sandy desert (RNPSD)***

The Central Mountain ranges of Afghanistan, which are the western extensions of the Hindu Kush Mountains, are to the north. The Sulaiman Mountains in Pakistan are to the east, the Central Makran Range in Pakistan is to the south, and the Central Persian desert basins region is to the west. The granite Chagai Hills, which stretch along the Afghan-Pakistan border, run across the middle.


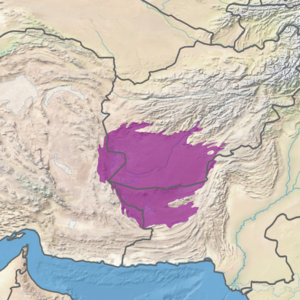


**Fig 12.** The Registan–North Pakistan sandy desert ecoregion distribution

(Source: https://en.wikipedia.org/wiki/Registan–North_Pakistan_sandy_desert)

**Protected areas**

Hamun Lake

**Macrofungi recorded**

No records

1. **Biome: Flooded grasslands and savannas**

Large expanses or complexes of flooded grasslands make up the WWF biogeographical system's terrestrial biome of flooded grasslands and savannas. This biome can be found on four of the world's continents. The Everglades, Pantanal, Lake Chad flooded savanna, Zambezian flooded grasslands, and Sudd all have globally significant flooded savannas and grasslands.


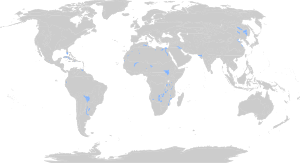


**Fig 13.** Worldwide distribution of Flooded grasslands and savannas

(Source: https://en.wikipedia.org/wiki/Flooded_grasslands_and_savannas)

1. ***Indus River delta Arabian sea mangroves (IRM)***

**Status:** Critical/ Endangered

These are the Gulfs of Kutch and Khambhat in Gujarat, India, and a huge mangrove ecoregion on the Arabian Sea coast of Sindh Province, Pakistan. These mangroves are the world’s seventh largest mangrove forest. The Indus River Delta is a massive alluvial fan made up of mud flats interlaced with channels and surrounded by mangrove forests. The delta has shrunk dramatically in the twentieth century, as industrialization and coastal management policies have weakened the delta's resiliency. In 1990, mangroves covered 16 percent of the delta, but by 2017 that number had dropped to 10%. Much of the forested area has been lost, and the remaining areas are under threat, owing to activities such as dykes that make the soil unsuitable for mangroves, as well as encroachment.


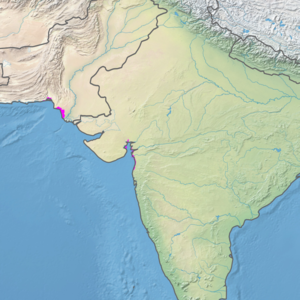


**Fig 14.** Indus River delta Arabian sea mangroves ecoregion distribution

(Source: https://en.wikipedia.org/wiki/Indus_River_Delta–Arabian_Sea_mangroves)

**Protected Areas**

Marho Kotri, Cut Munarki Chach, Mirpur Sakro

**Dominant Plants**

Mangroves are an essential habitat where salt and fresh water mingle, supporting a variety of plants that have evolved to thrive in this salty climate, as well as enormous numbers of fish and crustaceans that feed, shelter, and breathe in the waters underneath the tree’s roots.

**Macrofungi recorded**

No records

**Justification of Ecoregion Delineation by WWF (2001)**

To determine the area of the mangroves represented in this ecoregion, MacKinnon's (1997) computerised map of original vegetation was used.

1. ***Rann of Kutch seasonal marsh***

**Status: Relatively stable/Intact**

The Rann of Kutch Seasonal Salt Marsh is located at the mouth of the Luni River, which runs southward from the Aravalli Hills before dissipating in the dry, arid salt flats that make up this ecoregion. Geographically, the ecoregion spans the northwestern Indian state of Gujarat and the Sind Desert region of southern Pakistan, all of which lie along the Tropic of Cancer. The Little and Great Ranns were extensions of the shallow Arabian Sea since the Mesozoic until geological uplift severed the link, forming a large lake that was still navigable throughout Alexander the Great's reign. However, centuries of silting have resulted in a large saline mudflat. The mudflat becomes inundated during the brief wet season. After then, it becomes parched as a result of the extended dry season's persistent, scorching heat; the ecoregion has one of the highest yearly evaporation rates in the region. The average summer temperature is 44°C, but it can reach 50°C, and the minimum winter temperatures are near or even below that.


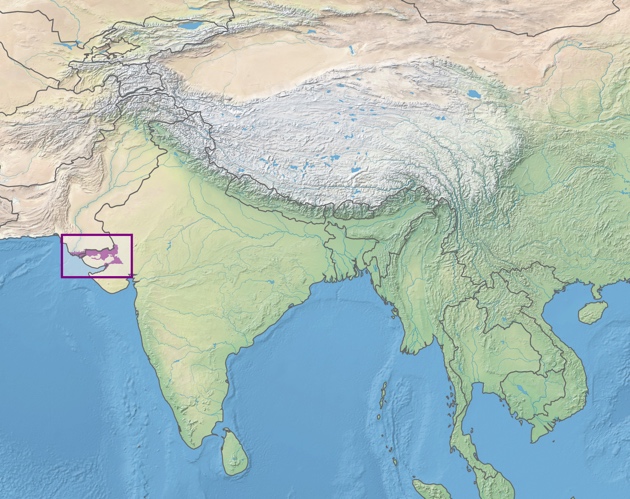


**Fig 15.** Distribution of Rann of Kutch ecoregion

(Source: https://en.wikipedia.org/wiki/Rann_of_Kutch)

**Dominant Plants**

Grass and dry thorn scrub such as *Apluda aristata*, *Cenchrus* spp., *Pennisetum* spp., *Cymbopogon* spp., *Eragrostis* spp., and *Elionurus* spp. make up the vegetation (Puri et al. 1989). Except in the bets, where the exotic *Prosopis juliflora* has begun to overrun, there are few huge trees. The wild asses eat seedpods from the Prosopis all year long. The vegetation of the Little Rann is divided into two types: *Salvadora* *scrub* and tropical *Euphorbia* scrub (Champion and Seth 1968).

**Macrofungi recorded**

No records

**Justification of Ecoregion Delineation by WWF (2001)**

To identify the limits of this ecoregion, MacKinnon's (1997) digitised map of original habitat was used, which illustrates the extent of the Rann of Kutch salt marshes. The Thar Desert biogeographic province includes the Rann of Kutch Seasonal Salt Marsh and the Indus Valley Desert.

1. **Biome: Montane grasslands and shrublands**

High elevation (montane and alpine) grasslands and shrublands, such as the puna and paramo in South America, subalpine heath in New Guinea and East Africa, Tibetan plateau steppes, and other comparable subalpine habitats around the world, make up this significant habitat category.


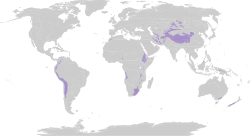


**Fig 16.** Worldwide distribution of Montane grasslands and shrublands

(Source: https://en.wikipedia.org/wiki/Montane_grasslands_and_shrublands)

1. ***Karakorem West Tibetan Plateau alpine steppe (KWTP):***

**Status:** Vulnerable

In Tibet, the alpine zone encompasses those locations where the average temperature in July, the hottest month of the year, is less than 10 degrees Celsius. Almost the whole plateau falls inside this "high cold" alpine zone, with the exception of the southern river valleys of the Indus, Sutlej, and Tsangpo (including the Lhasa Valley). Moisture determines whether a place supports meadow, steppe, or alpine desert vegetation within the alpine zone. Vegetation varies from dense scrub to meadow to steppe to desert as precipitation decreases northwestward. Forests are only found in valleys in Tibet. Because of the cold, continental temperature and the plateau's location above the 10^o^ isotherm, forests never grow on the plateau.

To the north of the Central Himalayan Crest is the vast Yarlung Tsangpo (Zambo) River Valley, which runs parallel to the confluence of the Asian and Indian continental plates. It is bounded on one side by the Himalaya and on the other by Tibetan fringe ranges. The Nyenching Tangula and Gangdise Mountain Ranges, which run parallel to the Himalaya around 150 to 200 kilometres north, are part of the latter.


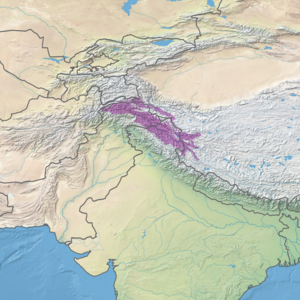


**Fig 17.** Karakorem West Tibetan Plateau alpine steppe ecoregion distribution

(Source: <https://en.wikipedia.org/wiki/Karakoram–West_Tibetan_Plateau_alpine_steppe>)

**Protected Areas**

Karakoram, Khunjerab, Naltar, K2 National Park, Nar Nullah, Astore, Baltistan

**Dominant Plants**

The vegetation on the valley bottom along the river's middle reaches consists of grasses, scattered forbs, and shrubs that may be scattered or clumped depending on water availability. Steppe and shrublands are typical vegetation on both the north and south slopes of the valley. *Stipa bungeana*, *Pennisetum flaccidum*, *Aristida triesta*, *Orinus thoroldii*, *Artemisia webbiana*, and *Trikeraia hookeri* are some of the steppe's most common grasses and forbs. The first two are found across Central Asia, whereas the last four are found only on the Tibetan Plateau. *Sophora moorcroftiana*, *Leptodermis sauranja*, *Ceratostigma griffithii*, and others are shrubs of Tibetan Plateau most of which are endemic.

Cold steppe vegetation replaces the temperate steppe shrublands on the lower slopes at elevations exceeding 4,400 m on the valley's sides. The grass *Stipa purpurea* and the bushes *Potentilla fruticosa* and *Lonicera tibetica* are prominent in the east, while *Caragana versicolor* is dominating in the west. *Kobresia pygmaea* and cushion plants like *Arenaria*, *Androsace*, and *Oxytropis* dominate stable slopes over 5,000 metres.

**Macrofungi recorded**

*Abortiporus, Agaricus, Ascobolus, Aureoboletus, Bjerkandera, Boletus, Cerioporus, Chalciporus, Chlorophyllum, Coprinellus, Coprinopsis, Cortinarius, Cystodermella, Fomes, Ganoderma, Geastrum, Geopora, Geopyxis, Gloeophyllum, Gyrodon, Helvella, Hygrophorus, Inocybe, Lactarius, Lactifluus, Laetiporus, Leccinellum, Leccinum, Lycoperdon, Mallocybe, Mycena, Omphalotus, Paralepista, Phaeolepiota, Phaeolus, Phallus, Picipes, Polyporus, Porphyrellus, Ramaria, Rigidoporus, Russula, Simocybe, Suillus, Trametes, Tricholoma, Tricholomopsis, Tulostoma, Tylopilus, Xanthoconium, Xanthoporia, Xerocomus.*

**Justification of Ecoregion Delineation by WWF (2001)**

Ecoregion lines were created from a Tibetan rangeland’s computer map (Commission on Integrated Survey of Natural Resources 1992). Warm and cool semi-arid montane rangeland, warm and semi-arid montane scrub grassland, and warm, cool, and damp subalpine sparse woodland and scrub meadow and grassland are all found in this region.

1. ***Northwestern Himalayan alpine scrub & meadows (NWHASM)***

**Status:** Relatively Stable/Intact

This ecoregion includes alpine scrub and meadows between 3,300 and 3,600 metres in the Himalayan Range's northeastern slopes. The ecoregion is found largely in the Vale of Kashmir and its surrounding mountains in northwestern India and northern Pakistan.


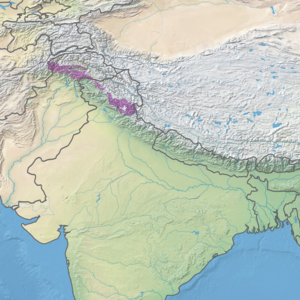


**Fig 18.** Northwestern Himalayan alpine scrub & meadows ecoregion distribution

(Source: https://en.wikipedia.org/wiki/Northwestern_Himalayan_alpine_shrub_and_meadows)

**Dominant Plants**

This ecoregion has a diverse range of vegetation types. The vegetation consists of a stunted birch-rhododendron community in low near-evergreen scrub. Only five rhododendron species have been identified in this zone (Rao 1994), compared to over sixty in the eastern Himalaya. The alpine meadow, which rises above the scrub, is home to a varied herb community that includes *Doronicum*, *Delphinium*, *Gentiana*, *Meconopsis*, *Pedicularis*, *Anemone*, *Aster*, *Polygonum*, *Primula*, and *Mertensia*, among others. Cushion-forming plants including Caragana, Saxifraga, Draba, and Gypsophila can be found in the Scree habitat. In the Fumariaceae, Primulaceae, Saxifragaceae, and Scrophulariaceae families, a large number of alpine and subalpine herbs are endemic, and many are endangered (WWF and IUCN 1995).

**Protected Area**

Chitral Gol National Park

**Macrofungi recorded:**

*Amanita, Bovista, Calvatia, Chroogomphus, Fomitopsis, Funalia, Hebeloma, Heterobasidion, Laccaria, Lactarius, Marasmiellus, Melanogaster, Melanoleuca, Naucoria, Phellinus, Pleurotus, Rhizopogon, Rhodocollybia, Russula, Suillus, Tricholomopsis, Xanthoconium.*

**Justification of Ecoregion Delineation by WWF (2001)**

MacKinnon (1997) classified the Himalayan Range into four subunits along the longitudinal axis in a prior study of conservation units in the Indo-Malayan region: the Northwest Himalayas, West Nepal, Central Himalayas, and Eastern Himalayas. According to our principles for creating ecoregions, MacKinnon's (1997) vegetation map was utilised to divide the alpine scrub and meadows into a new ecoregion, Northwestern Himalayan Alpine Shrub and Meadows (i.e., represent ecosystems of regional extent within separate ecoregions).

1. ***Sulaiman Range Alpine meadows (SRAM)***

This ecoregion runs through the Chitral and Swat side valleys in the north, as well as parts of the Safed Koh and Waziristan ranges. It then descends to the high-elevation portions of southwestern Pakistan's Chialtan, Toba Kakar, and Takht-I-Suleiman ranges, as well as northeastern Afghanistan's Takht-I-Suleiman range.

Most of the land is located between 1,600 and 3,500 metres above sea level. This ecoregion is characterised by high aridity and large temperature extremes. The annual precipitation averages less than 225 millimetres. The temperature reaches a maximum of 40°C and a minimum of -12°C during the winter. The majority of precipitation comes as rain or snow during the winter, with snow persisting on the higher peaks from December to March. The monsoon's effect causes the foothills of Safed Koh and Waziristan hills, as well as the majority of the south, to get equal quantities of rainfall. Winter accounts for nearly a quarter of the yearly rainfall, however rain falls throughout the year.


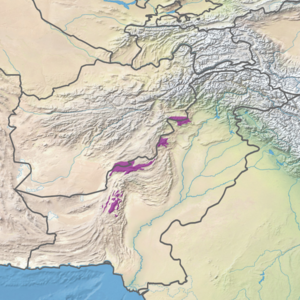


**Fig 19.** Sulaiman Range Alpine meadows ecoregion distribution

(Source: https://en.wikipedia.org/wiki/Sulaiman_Range_alpine_meadows)

**Protected Area**

Koh-e-Geish

**Dominant Plants**

Gravel and scree slopes with isolated tufts of bunch grasses and thorny hassock-shaped groupings of plants like *Onobrychis* and *Acantholimon* spp. make up this ecoregion. In gullies, the forest cover is sparse and dense. Western Himalayan evergreen sclerophyllous forests and woodlands that mimic the flora of the Mediterranean region are prevalent in the lower parts of the Chitral, Safed Koh, Nuristan, and Waziristan Hills, where the moisture level is higher due to the monsoon's effect. Fagaceae species, such as *Quercus ilex* (only found in the Northern Waziristan, Koh-i-Safed, and Chitral foothills), east oleander (*Nerium*), tropical *Adhatoda*, and *Fraxinus xanthoxyloides*, are among them. Between 2,000 and 3,300 metres, the lower slopes of Takhatu, Zarghun, Wam-Pilghar, and Toba Kakar contain dry alpine steppe with Irano-Turanian affinities. Cupressaceae, Anacardiaceae, Pinaceae, Oleaceae, and Fagaceae are the families that make up this group. Typical species at this height include *Juniperus macropoda*, *Juniperus polycarpos*, *Pinus gerardiana*, and *Pinus wallichiana*. The understory shrubs and perennial grasses of this ecoregion are Ephedraceae, Labiatae, Gramineae, Compositae, and Leguminosae. *Artemisia* spp., *Astragalu* spp., *Cotoneaster persica*, *Berberis baluchistani*, and *Ephedra intermedia* are a few examples. Flowering plant species include *Ferrula oopoda*, *Eremurus stenophylla*, and *Saliva* spp.

**Macrofungi recorded:** *Pyrofomes*

**Justification of Ecoregion Delineation by WWF (2001)**

From MacKinnon's Baluchistan subunit, five ecoregions were identified: Suleiman Range Alpine Meadows South Iran Nubo-Sindian Desert and Semi-Desert, Baluchistan Xeric Woodlands , Rajasthan-North Pakistan Sandy Desert , and East Afghan Montane Coniferous Forests. All five ecoregions extend westward, with a component of each ecoregion extending outside the scope of this study. To the north, there are the Hindu Kush mountains, and to the south and west, there are the Anatolian-Iranian Desert, Iranian Desert, and Caucaso-Iranian highlands.

**References**

The document is prepared by consulting Wikipedia for maps, WWF official website for description of biome and ecoregions and authors’s own contribution for enlisting macrofungi of each ecoregion.
